# GNG5 is a novel regulator of Aβ42 production in Alzheimer’s disease

**DOI:** 10.1038/s41419-024-07218-z

**Published:** 2024-11-11

**Authors:** Chunyuan Li, Yan Yang, Shiqi Luo, Wenying Qiu, Xia Wang, Wei Ge

**Affiliations:** 1 Key Laboratory of Immune Microenvironment and Disease (Ministry of Education), Tianjin Institute of Immunology, Department of Immunology, School of Basic Medical Sciences, Tianjin Medical University, Tianjin, China; 2 The State Key Laboratory for Complex, Severe, and Rare Diseases, Department of Immunology, Institute of Basic Medical Sciences Chinese Academy of Medical Sciences, School of Basic Medicine Peking Union Medical College, Beijing, China; 3 Institute of Basic Medical Sciences, Neuroscience Center, National Human Brain Bank for Development and Function, Chinese Academy of Medical Sciences, Department of Human Anatomy, Histology and Embryology, School of Basic Medicine, Peking Union Medical College, Beijing, China

**Keywords:** Neurological disorders, Cognitive neuroscience

## Abstract

The therapeutic options for Alzheimer’s disease (AD) are limited, underscoring the critical need for finding an effective regulator of Aβ42 production. In this study, with 489 human postmortem brains, we revealed that homotrimer G protein subunit gamma 5 (GNG5) expression is upregulated in the hippocampal–entorhinal region of pathological AD compared with normal controls, and is positively correlated with Aβ pathology. In vivo and in vitro experiments confirm that increased GNG5 significantly promotes Aβ pathology and Aβ42 production. Mechanically, GNG5 regulates the cleavage preference of γ-secretase towards Aβ42 by directly interacting with the γ-secretase catalytic subunit presenilin 1 (PS1). Moreover, excessive GNG5 increases the protein levels and the activation of Rab5, leading to the increased number of early endosomes, the major cellular organelle for production of Aβ42. Furthermore, immunoprecipitation and immunofluorescence revealed co-interaction of Aβ42 with GPCR family CXCR2, which is known as the receptor for IL-8, thus facilitating the dissociation of G-proteins βγ from α subunits. Treatment of Aβ42 in neurons combined with structure prediction indicated Aβ42 oligomers as a new ligand of CXCR2, upregulating γ subunit GNG5 protein levels. The co-localizations of GNG5 and PS1, CXCR2 and Aβ42 were verified in eight human brain regions. Besides, GNG5 is significantly reduced in extracellular vesicles (EVs) derived from cerebral cortex or serum of AD patients compared with healthy cognition controls. In brief, GNG5 is a novel regulator of Aβ42 production, suggesting its clinical potential as a diagnosis biomarker and the therapeutic target for AD.

**Figure:**
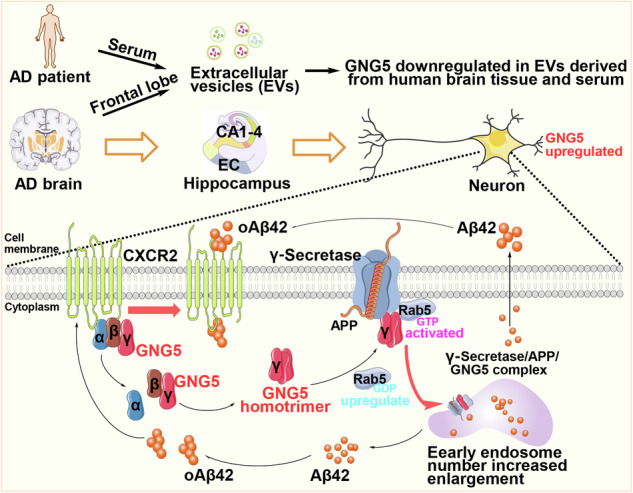
The GNG5 content in EVs derived from serum and brain tissue of patients with AD significantly reduced. The GNG5 expression in the hippocampal-entorhinal neurons of donors with pathological AD significantly increased, and can exist in homotrimer subtypes. GNG5 expression positively correlates with Aβ pathology and Aβ42 production. Homotrimer-GNG5 binds to the γ-secretase catalytic subunit PS1 and preferentially generates Aβ42 in early endosome. GNG5 leads to enhanced Rab5 protein and activation levels, increased number of early endosome, promoting Aβ42 production. Further, Aβ42 binds to CXCR2 to upregulate GNG5 levels in a feedback loop.

The GNG5 content in EVs derived from serum and brain tissue of patients with AD significantly reduced. The GNG5 expression in the hippocampal-entorhinal neurons of donors with pathological AD significantly increased, and can exist in homotrimer subtypes. GNG5 expression positively correlates with Aβ pathology and Aβ42 production. Homotrimer-GNG5 binds to the γ-secretase catalytic subunit PS1 and preferentially generates Aβ42 in early endosome. GNG5 leads to enhanced Rab5 protein and activation levels, increased number of early endosome, promoting Aβ42 production. Further, Aβ42 binds to CXCR2 to upregulate GNG5 levels in a feedback loop.

## Introduction

Alzheimer’s disease (AD) is a prevalent neurodegenerative condition. The main pathological manifestation of AD identified during autopsy is the deposition of amyloid beta peptide (Aβ) plaques, which is an important component of A and C evaluations in the ABC scoring system [1]. Aβ plaques are mainly composed of peptides, 40 or 42 amino acids in length, and early endosomes are a major site for the cleavage of amyloid precursor protein (APP) into Aβ [2]. Empirical evidence from in vivo and in vitro experiments associates high neurotoxicity with the oligomeric forms of the 42 amino acid peptides. γ-secretase is the only reported enzyme that cleaves APP to Aβ40 or Aβ42. Although structural biology analysis does not demonstrate a clear preference of γ-secretase for generating Aβ40 or Aβ42, individuals with higher pathological aggregation of Aβ42 exhibit considerable cognitive impairment, except for those with mutations in APP and gamma-secreting enzymes. Consequently, unidentified genes regulating the preference of APP cleavage during the progression of AD remain an area of interest.

The postmortem analysis of 489 brains from the National Human Brain Bank for Development and Function, the largest brain repository in China, revealed that the A score is critical in evaluating pathological grade, indicating the continued significance of Aβ as a crucial therapeutic target for AD. Notwithstanding, monoclonal antibody drugs designed to clear Aβ plaques, though curatively efficacious in the early stages of AD, demonstrate reduced efficacy in the symptomatic stages [3]. Currently, the Food and Drug Administration (FDA) has not approved any drugs targeting γ-secretase. Clinical trials of Eli Lilly’s semagacestat, a small-molecule γ-secretase inhibitor, failed at Phase 3, with observed adverse events such as goblet cell hyperplasia and thymus atrophy, potentially linked to Notch cleavage inhibition [4]. Furthermore, gosuranemab, a monoclonal antibody to N-terminal tau-in patients with early AD, did not yield notable effects in cognitive and functional assessments [5]. Therefore, identifying genes that regulate early Aβ peptides, particularly Aβ42 formation, and restraining the oligomerization of Aβ42 is a promising approach for early diagnosis and intervention in AD, potentially benefiting patients with AD.

This study involved the analysis of 401 human brain tissues obtained from the National Human Brain Bank for Development and Function. We examined five subregions within the hippocampus–entorhinal system known for their significance in cognition. Our findings reveal that G protein subunit gamma 5 (GNG5) regulated Aβ42 production by directly binding to γ-secretase; the knockdown of GNG5 ameliorated cognition in AD model mice. Additionally, the significant decrease in serum GNG5 levels within extracellular vesicles (EVs) of patients in the early stages of AD indicates its potential as an early diagnostic marker for AD.

## Results

### GNG5 is localized in early endosomes and is upregulated in the brain tissues of donors with pathological AD

Based on the analysis of 489 postmortem brains from the National Human Brain Bank for Development and Function, we used multiple linear regression to analyze the relationship between AD neuropathological changes and “ABC” scores (A: Amyloid, B: Braak, C: CERAD). The A score is of the most significance in evaluating the pathological grade, indicating that Aβ is still an important therapeutic target for AD.

$$\hat{Y}={\rm{a}}A+{\rm{b}}B+{\rm{c}}C+{\rm{d}}$$

$${\rm{a}}=0.519,{\rm{b}}=0.165,{\rm{c}}=0.347$$

*where [Y* *^ stands for AD neuropathologic changes; A, B, C stands for the “ABC” score; a, b, c stands for coefficients of A, B, C, respectively]*

**Fig. 1:**
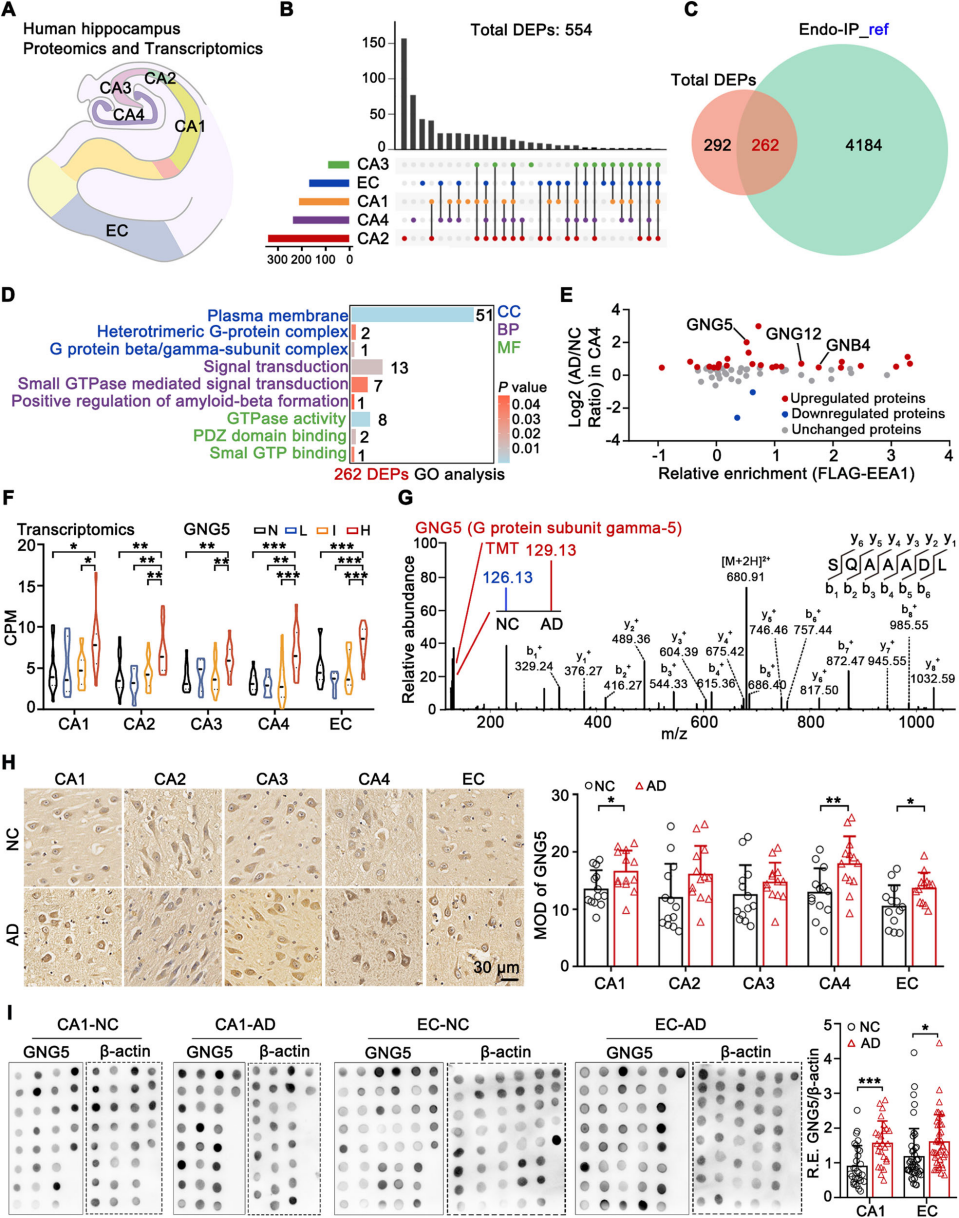
G protein subunit gamma 5 (GNG5) is localized in early endosomes and is upregulated in the brain tissues of donors with pathological Alzheimer’s disease (AD). **A** Schematic diagram of hippocampal–entorhinal subregions for proteomic and transcriptomic analysis. **B** UpSet plot illustrating intersections of differentially expressed proteins (DEPs) among hippocampal subregions (CA1, CA2, CA3, CA4) and entorhinal cortex (EC). The upper bars show the number of identified proteins between the sample groups marked below. **C** Venn diagram of the 554 DEPs and 4446 endosomal proteins enriched with FLAG-EEA1 (Endo-IP_ref). Endo-IP_ref data can be found in Supplementary Table 1 of Professor J. Wade Harper’s research. research doi: 10.1038/s41467-022-33881-x [10]. **D** Gene Ontology (GO) term enrichment analysis for 262 DEPs, performed using the Wu Kong platform (https://www.omicsolution.com/wkomics/main/). CC: cellular component, BP: biological process, MF: molecular function. **E** The distribution of DEPs enriched in these GO terms in CA4 subregion and in the Endo-IP_ref data. **F** Counts per million (CPM) of *GNG5* in transcriptomic analysis of CA1, CA2, CA3, CA4, and EC subregions from donors with neuropathological assessment N, L, I, or H. N, *n* = 13–15; L, *n* = 5–6; I, *n* = 17; H, *n* = 14. **G** Representative MS/MS spectra of the identified peptide of GNG5. Intensities of the tandem mass tag (TMT) precursor ions represent the relative level of peptide in CA1 subregion. **H** Representative immunohistochemical images and of GNG5 labeled with anti-GNG5 antibody (Abcam, ab238835) in the hippocampal–entorhinal five subregions. Mean optical density (MOD) was determined. Scale bar, 30 µm. NC (*n* = 13, 84.8 ± 9.1 yr), AD (*n* = 13, 89.8 ± 5.4 yr). **I** Dot blot and densitometry analyses of GNG5 levels (Bioworld Technology, BS61200) in CA1 and EC from NC and pathological AD donors. β-actin was used as the reference control. For CA1, NC (*n* = 29, 85.9 ± 8.4 yr), AD (*n* = 26, 84.2 ± 6.4 yr). For EC, NC (*n* = 45, 88.6 ± 7.8 yr), AD (*n* = 41, 87.4 ± 6.8 yr). Data are presented as mean value ± SD. Two-tailed unpaired Student’s *t*-test was used for two groups and one-way ANOVA with Turkey post hoc test for multiple groups. *p < 0.05, **p < 0.01, ***p < 0.001.

Crucial factors controlling AD development by regulating Aβ were screened using tandem mass tag (TMT)-labeled quantitative proteomics and transcriptomics analyses in the hippocampal–entorhinal system from postmortem cognitively normal controls (NC, *n* = 4) and donors with pathological AD (*n* = 4) (Figs. 1A and S1, Table S1). This system plays an essential role in learning and memory formation, including hippocampal subregions [cornu ammonis (CA) 1, CA2, CA3, CA4] and the entorhinal cortex (EC) [6–8]. Based on the false discovery rate <0.01 and unique peptides ≥ 2, we identified 5735 and 5743 confident proteins in CA1 subregion in proteomic SET1 and SET2, respectively; a total of 5552 proteins overlapped in both the SETs (Table S2, Fig. S2A). Correlation analysis illustrated overall reproducibility of protein expression profiles between SET1 and SET2 (Fig. S2B), indicating the robustness and repeatability of the mass spectrometry (MS) method. An identical MS method was employed for the subregions; 5552, 5735, 5736, 5744, and 5744 confident proteins were successfully identified in CA1, CA2, CA3, CA4, and EC, respectively (Table S2). With the differential expression threshold (between pathological AD and NC) defined as the 90% prediction interval value (Fig. S2C), 210, 339, 88, 235, and 167 differentially expressed proteins (DEPs) were identified in CA1, CA2, CA3, CA4, and EC, respectively (Fig. S2D), with a total of 554 DEPs identified from the union set of five subregions (Fig. 1B).

Considering that the early endosome is the major site of APP processing, we matched the 554 DEPs with endosomal proteins identified by endosome IP/MS [9, 10] to identify crucial endosomal-related factors regulating Aβ production; the findings suggested 262 shared DEPs (Fig. 1C). Gene Ontology (GO) annotation for the 262 shared DEPs was enriched in the G protein beta/gamma-subunit complex, positive regulation of amyloid beta formation, and GTPase activity (Fig. 1D), implying that G protein beta and gamma subunits may have a significant impact on Aβ formation. We then analyzed the distribution of these shared DEPs that enriched in these GO terms, and identified three upregulated G protein beta or gamma subunits (GNG5, GNG12, and GNB4) (Fig. 1E) [11]. Transcriptomic analysis showed that *GNG5* mRNA levels were significantly higher in all the five subregions of donors with AD in the H-pathology stage compared with those in the non (N)-, low (L)-, and immediate (I)-pathology stages (Fig. 1F, Table S3). *GNG12* was upregulated exclusively in the EC of donors with pathological AD in the high (H)-pathology stage (Fig. S2E), and *GNB4* was downregulated in the CA2, CA3, and EC subregions of donors with pathological AD (Fig. S2F). GNG5 presence in the hippocampal–entorhinal region of donors in the NC and AD groups was verified using MS/MS identification (Fig. 1G). Moreover, immunohistochemistry (IHC) findings in postmortem hippocampal sections from 13 NCs and 13 donors with pathological AD showed that among the subregions GNG5 levels were significantly increased in donors with pathological AD in the CA1, CA4, and EC regions, while they tended to increase non-significantly in CA2 and CA3 (Figs. 1H and S3A). In addition, we evaluated the expression levels of GNG5 in CA1 and EC tissues. Due to the limitations associated with the application of GNG5 antibodies, the recombinant GNG5 protein (purity >95%) was utilized to assess the efficiency and specificity of the anti-GNG5 antibodies. Interestingly, recombinant GNG5 can exist as monomer and oligomer, and appears to be predominantly homotrimer (Fig. S3B). The dot blot data from 85 postmortem human brain tissues demonstrated significantly higher GNG5 protein levels in the CA1 and EC regions of donors with pathological AD compared to those of the respective NCs (Fig. 1I and S3C). Dot blot with β-actin or total amount of loaded proteins stained with ponceau as reference controls demonstrated the same conclusion. Thus, GNG5 is upregulated in hippocampal-entorhinal system of AD with potential involvement in regulating Aβ production.

### Increased GNG5 level promotes Aβ42 production and causes neuronal dysfunction

**Fig. 2:**
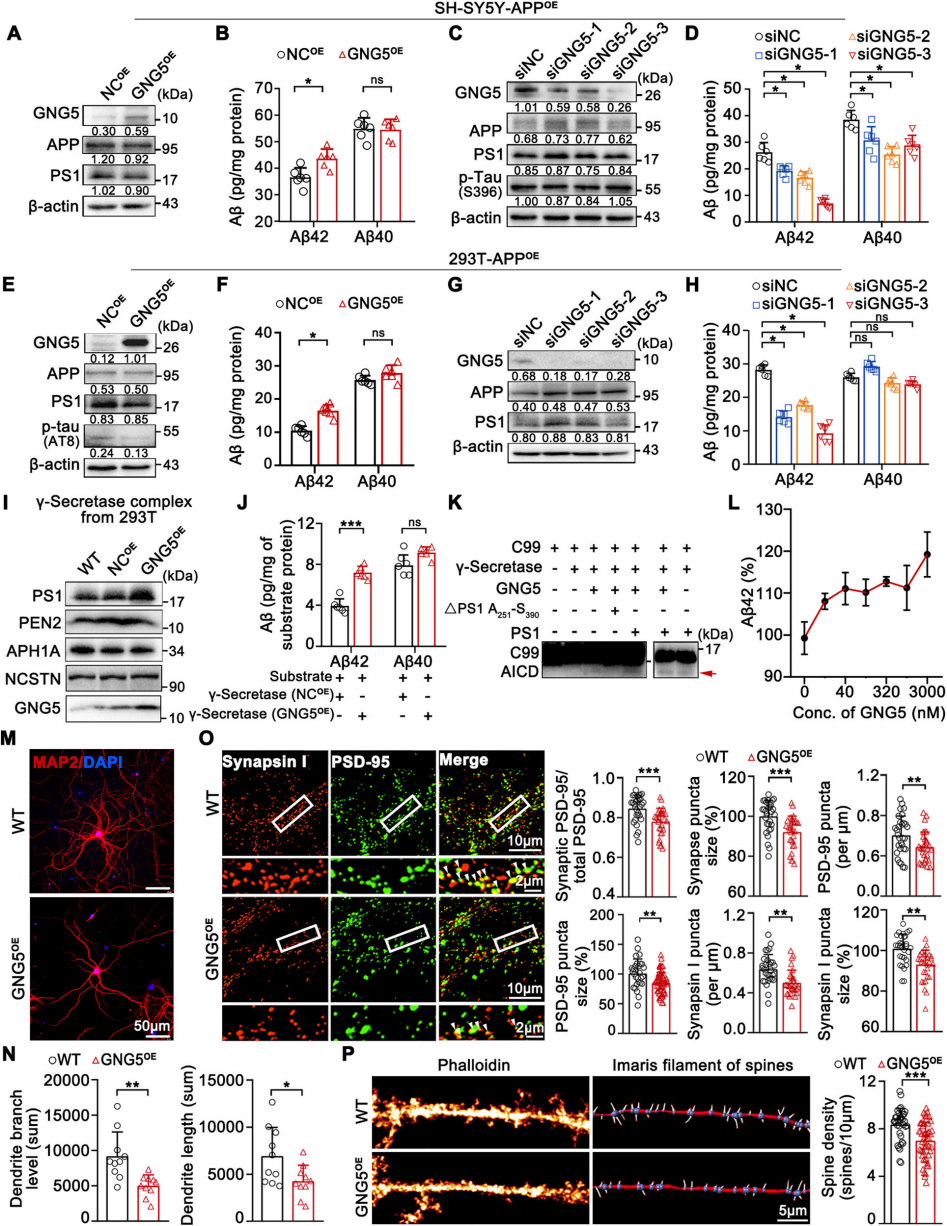
Increased GNG5 promotes Aβ42 production and causes neuronal dysfunction. **A**, **B** Establish of GNG5 overexpressed cell model SH-SY5Y-APP^OE^-GNG5^OE^. **A** Western blot detection of GNG5, APP, and PS1 levels in cells. **B** Enzyme-linked immunosorbent assay (ELISA) measurement of Aβ42 and Aβ40 levels in cellular supernatant. Normalized (target protein blot/reference blot) quantitative results (densitometry) calculated using Image J software are shown under each blot. **C**, **D** Knockdown of GNG5 in SH-SY5Y-APP^OE^ cells. **C** Western blot detection of GNG5, APP, PS1, and p-tau (S396) in cells. **D** ELISA measurement of Aβ42 and Aβ40 levels in cellular supernatant. **E**, **F** Establish of GNG5 overexpressed cell model 293T-APP^OE^-GNG5^OE^. **E** Western blot detection of GNG5, APP, PS1, and p-tau (AT8) protein levels in cells. **F** ELISA measurement of Aβ42 and Aβ40 levels in cellular supernatant. **G**, **H** Knockdown of GNG5 in 293T-APP^OE^ cells. **G** Western blot detection of GNG5, APP, and PS1 in cells. **H** ELISA measurement of Aβ42 and Aβ40 levels in cellular supernatant. **I**–**L** In vitro γ-secretase cleavage assay. **I** γ-secretase complex extracted from wild type 293T, 293T-NC^OE^, and 293T-GNG5^OE^ cells. Western blot verification of PS1, PEN2, NCSTN, APH1A and GNG5 levels in the complex. **J** Levels of Aβ42 and Aβ40 generated in the γ-secretase cleavage assay using ELISA. Cytoplasmic proteins extracted from Neuro2a-APP^OE^ cells were used as substrates. **K** Comparation of cleavage activity of γ-secretase using recombinant C99 as substrate and γ-secretase complex extracted from WT 293T cells. Recombinant GNG5, full length PS1, and PS1 Ala_251_–Ser_390_ fragment were added to this system. Quantification of AICD (red arrow) was monitored using a monoclonal antibody against the *C*-terminal 20 amino acids (C1/6.1, BioLegend, 802801). AICD, APP Intracellular Domain, the left domain after cleavage of Aβ42 or Aβ40 from C99. **L** ELISA measurement of Aβ42 levels at different concentrations of recombinant GNG5 in the in vitro cleavage assay with C99 as substrate and WT γ-secretase complex. Representative confocal images depicting dendritic staining of MAP2 (**M**), and the statistics of total dendrite branch number and total dendrite length (**N**) in WT and GNG5^OE^ rat primary hippocampal neurons. Scale bar, 50 μm. **O** Representative confocal images depicting synaptic staining of presynaptic marker Synapsin I (red) and postsynaptic marker PSD-95 (green) in WT and GNG5^OE^ rat primary hippocampal neurons. Single synaptic number was quantified as colocalized pre- and postsynaptic puncta. The boxed areas are enlarged below the original images. Histograms depicting the relative density or size level of single synapse, PSD-95 and Synapsin I puncta in WT and GNG5^OE^ rat primary hippocampal neurons. *n* = 30 fields/group. Scale bar, 10 μm; inset, 2 μm. **P** Representative deconvolved images showing spine densities labeled with phalloidin in WT and GNG5^OE^ rat primary hippocampal neurons (left), and corresponding detailed 3D-rendered views of spines (right). Statistical results showing changes in spine density. Data are presented as the mean ± SD. *p* values were determined using unpaired two-tailed Student’s *t*-test for two groups and one-way ANOVA with Turkey post hoc test for multiple groups. *p < 0.05, **p < 0.01, ***p < 0.001, ns: not significant.

To explore whether GNG5 regulates the production of Aβ42 and Aβ40, we established stable cell models human neuroblastoma SH-SY5Y-APP^OE^-GNG5^OE^ and 293T-APP^OE^-GNG5^OE^ as well as relative controls. qRT-PCR and western blot verified overexpression of APP and GNG5 (Figs. 2A, E and S4A–C). Commercial ELISA kits (R&D, DAB142, DAB140B) were used to detect the content of secreted Aβ42 and Aβ40 peptides in cellular supernatant. To rule out potential nonspecific binding, synthetic human (aa1-42) and synthetic human Aβ (aa1-40) (purity >95%) were assayed for cross-reactivity of ELISA kit, and no significant cross-reactivity or interference was observed (data not shown). The results showed that overexpressed GNG5 significantly increased Aβ42 levels in SH-SY5Y-APP^OE^ (Fig. 2B) and 293T-APP^OE^ (Fig. 2F) cellular supernatants, without obvious change of Aβ40 levels. Knockdown of GNG5 significantly reduced Aβ42 levels in SH-SY5Y-APP^OE^ (Figs. 2C, D and S4C) and 293T-APP^OE^ (Figs. 2G, H and S4D) supernatants, with no alteration of Aβ40 levels except that in SH-SY5Y-APP^OE^, possibly due to unexpected compensatory mechanisms. No obvious alteration was induced by GNG5 overexpression or knockdown for APP (the Aβ producing substrate) and γ-secretase subunit presenilin 1 (PS1, the cleavage enzyme) [12] at transcriptional and expression levels in these indicated cells, except the siGNG3-3 which unexpectedly increased APP and PS1 mRNA levels in SH-SY5Y-APP^OE^ and 293T-APP^OE^ (Figs. 2A, C, E, G and S4B–E). Also, alteration of GNG5 did not affect p-Tau protein levels in SH-SY5Y-APP^OE^, 293T-APP^OE^, and Neuro-2a (Figs. 2C, E and S4F, G). In conclude, these results indicated that GNG5 could promote Aβ42 production without modulating the expression levels of APP and PS1.

Aβ peptides are generated from the sequential cleavage of C99, the *C*-terminal fragment of APP, by γ-secretase. To verify the enhancement of Aβ42 generation by GNG5, we performed in vitro γ-secretase cleavage assay (Fig. 2I–L) as reported by Shi group [13], using extracted γ-secretase complex and substrates from Neuro-2a-APP^OE^ cell (Fig. 2J) or recombinant C99 (Fig. 2K, L). We first extracted γ-secretase complex from wild type (WT) 293T, 293T-NC^OE^, and 293T-GNG5^OE^ cells. Western blot detected co-localization of four γ-secretase subunits (PS1, PEN2, NCSTN, and APH1A) and GNG5 (Figs. 2I and S4H) [13]. Using cytoplasmic extracts from Neuro-2a-APP^OE^ cells as the substrate, we found significantly enhanced Aβ42 production using γ-secretase extracted from 293T-GNG5^OE^ cells compared with that extracted from 293T-NC^OE^ cells, while the production of Aβ40 remained unchanged (Fig. 2J). Then, we used recombinant C99 as substrate with γ-secretase in the cleavage system. Recombinant GNG5 with full length PS1 or PS1 Ala_251_–Ser_390_ fragment (Fig. S4I) were added to this system. The results showed substantially increased generation of APP Intracellular Domain (AICD) peptides after GNG5 incubation (Fig. 2K), demonstrating enhanced γ-secretase activity and Aβ production. In addition, the Aβ42 production increased relative to the GNG5 concentration (Fig. 2L). Notably, the catalytic activity of PS1 requires cooperation of other three partners, we did observe that AICD peptide generation significantly increased when GNG5 and PS1 recombinant proteins were combined, compared to that with GNG5 alone, but not when GNG5 and a PS1 Ala_251_–Ser_390_ fragment were combined (Fig. 2K). These data suggested that GNG5 may contribute to Aβ42 production by interacting with PS1, but not specifically its active site residues (Asp_257_ and Asp_385_) [14].

Next, we investigated the impact of GNG5 overexpression on neuronal network function. We used MAP2 to characterize dendrites in newborn rat-derived primary neurons and found that GNG5 overexpression damages neuronal dendritic structures with less dendrites (Fig. 2M, N) [15]. Co-immunostaining of presynaptic marker Synapsin I and postsynaptic marker PSD-95 revealed significant decrease of Synapsin I- and PSD-95-positive puncta size and density, as well as the size and density of single synapses, in GNG5-overexpressing primary neurons compared with those in the WT group (Fig. 2O). Also, GNG5 overexpression decreased dendritic spine density in primary neurons (Fig. 2P). According to the literature, the toxic oligomers of Aβ42 (oAβ42) can lead to synaptic loss [16], which may be one of the key reasons for the synaptic loss caused by GNG5 overexpression. In this part, we found GNG5-induced synaptic loss in wild-type primary neurons, which generate only small amounts of Aβ42. This suggests that other signaling pathways, such as glial-mediated synaptic phagocytosis [17], may involve in this process. The exact mechanisms remain unclear and warrant further investigation. Totally, the above results demonstrated that GNG5 promoted Aβ42 generation and caused impaired synaptic and dendritic spine function.

### Increased GNG5 aggravates amyloid pathology in 5×FAD and FAD^4T^ mice

**Fig. 3:**
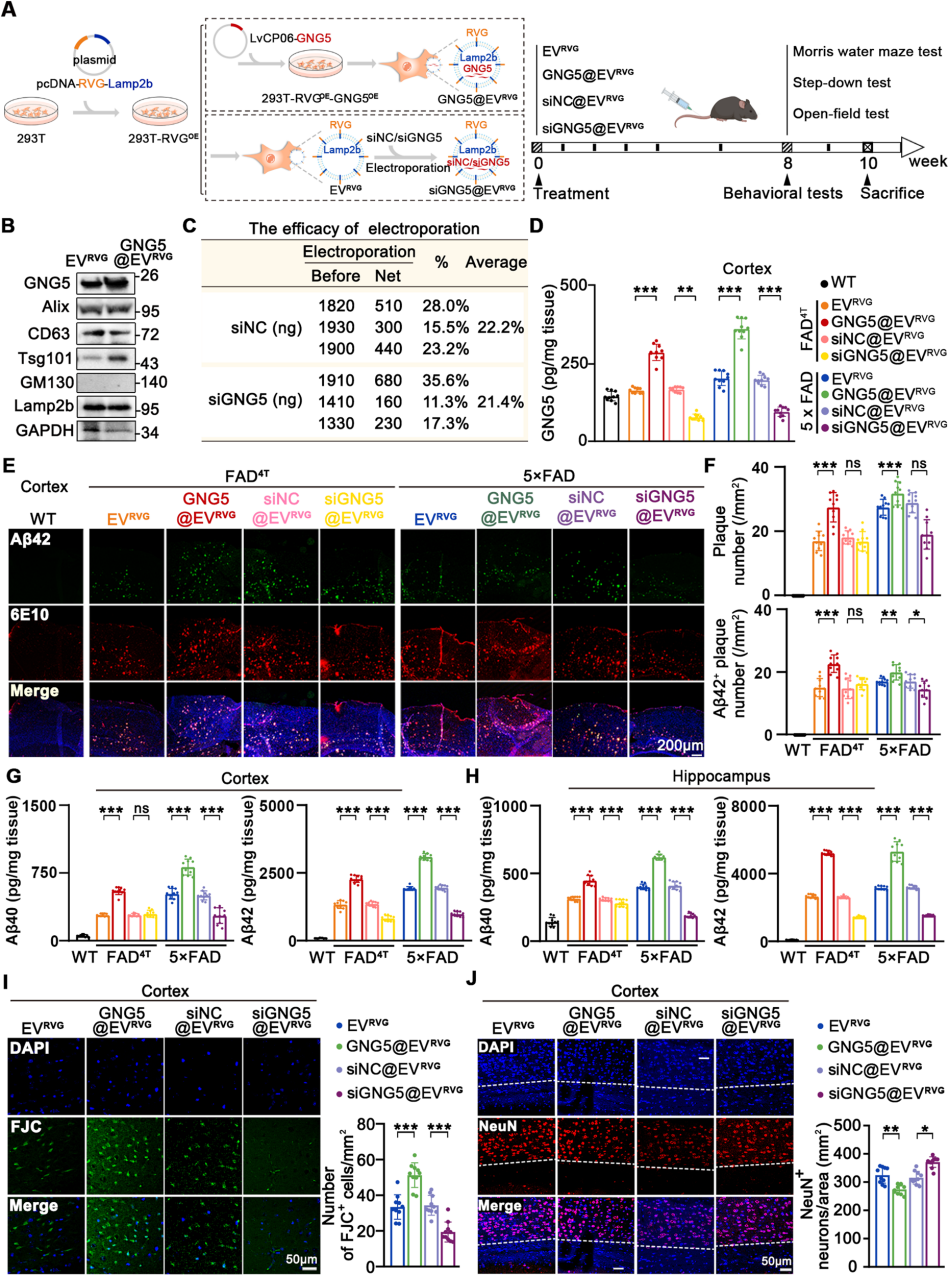
Elevated GNG5 levels aggravate amyloid pathology in 5×FAD and FAD^4T^ mice. **A** Scheme of preparing EV^RVG^, GNG5@EV^RVG^, and siGNG5@EV^RVG^ (left); schematic diagram of GNG5 intervention and subsequent behavioral examination in 5×FAD and FAD^4T^ mice (right). **B** Western blot detection of GNG5, EVs markers Lamp2b, CD63, Alix, Tsg101 and negative marker GM130 in EV^RVG^ and GNG5@EV^RVG^. **C** Encapsulate efficacy of siNC and siGNG5 into EV^RVG^. **D** Verification of GNG5 overexpression and knockdown in the cortex of 5×FAD and FAD^4T^ mice by ELISA. *n* = 10 mice per group. **E**–**J** Engineered EVs including EV^RVG^, GNG5@EV^RVG^, siNC@EV^RVG^, siGNG5@EV^RVG^ were injected into mice via intravenous tail vein. WT (female, 5-month-old, *n* = 10), 5×FAD (female, 5-month-old, *n* = 10 per group), FAD^4T^ (female, 4-month-old, *n* = 10 per group). **E**, **F** Representative fluorescence micrographs and quantification of amyloid plaques (anti-6E10) and Aβ42^+^ plaques (anti-Aβ42) in cortex of indicated mice. ELISA quantification of soluble Aβ42 and Aβ40 levels in cortex (**G**) and hippocampus (**H**). **I** Representative fluorescence micrographs of Fluoro-Jade C staining of cortical slices. **J** Representative confocal images of neuronal marker NeuN^+^ labeling neurons (red) in cortex. Data are presented as the mean ± SD. *p* values were determined using one-way ANOVA with Turkey post hoc test for multiple groups. *p < 0.05, **p < 0.01, ***p < 0.001, ns: not significant.

To explore the effect of GNG5 in vivo, we designed to establish neuronal targeted GNG5 overexpressing and GNG5 knockdown AD model mice by engineered EV deliver of exogenous GNG5 or siGNG5 [18], and to investigate Aβ pathology in brains and the cognition-related behavioral changes of mice (Fig. 3A). Two strains of AD model mice, FAD^4T^ and 5×FAD, were used to verify the results. According to previous reports [19, 20], the neuron-specific rabies virus glycoprotein (RVG) (YTIWMPENPRPGTPCDIFTNSRGKRASNG) peptide was fused to *N* terminus of exosomal protein Lamp2b and the decorated EVs specifically binds to the acetylcholine receptor.

GNG5@EV^RVG^ was secreted and enriched from supernatant of 293T-RVG^OE^-GNG5^OE^ simultaneously overexpressing RVG-Lamp2b and GNG5, the cell was constructed by sequentially stabilizing transfection of pcDNA GNSTM-3-RVG-10-Lamp2b-HA vector and LvCP06-GNG5 vector (Fig. 3A). Overexpressed RVG and GNG5, and EV markers (positive markers: ALIX, CD63, and TSG101; negative marker: GM130) were confirmed by qRT-PCR and western blot in transfected 293T-RVG^OE^ cell (Fig. S5A), 293T-RVG^OE^-GNG5^OE^ cell (Fig. S5B) and GNG5@EV^RVG^ (Figs. 3B and S5C). We also purified EV^RVG^ from 293T-RVG^OE^ cell supernatant followed by loading of siNC or siGNG5 by electroporation, thus generated siNC@EV^RVG^ and siGNG5@EV^RVG^ (Fig. 3A, C). These modifications do not appear to affect the morphology and size of the modified EVs based on electron microscopy (Fig. S5C), biological markers including Lamp2b, CD63, Alix, Tsg101 (Fig. 3B), and particle diameters (Fig. S5D) of engineered EVs.

To evaluate the efficacy of delivering GNG5 or siGNG5 to brain tissues, lipophilic long-chain carbocyanine dye PKH26-labeled EV^RVG^, GNG5@EV^RVG^, siNC@EV^RVG^, or siGNG5@EV^RVG^ were intravenously injected to 5×FAD mice. Clear PKH-26 signals were obtained in the brain tissues of mice injected with EV^RVG^, GNG5@EV^RVG^ or siGNG5@EV^RVG^, and weaker signals were observed in the liver, spleen, and myocardium (Fig. S5E), suggesting that RVG-Lamp2b modification could guide more EVs into the brain tissue. In addition, ELISA results demonstrated significant elevation of GNG5 in GNG5@EV^RVG^-injected brains and significant reduction GNG5 in siGNG5@EV^RVG^-injected brains, compared to relative controls, both in the cortex (Fig. 3D) and hippocampus (Fig. S5F), indicating effective delivery of GNG5 and siGNG5 to cortical and hippocampal tissues.

Immunostaining with anti-6E10 and anti-Aβ42 antibodies demonstrated more Aβ plaques and Aβ42^+^ plaques found in 5×FAD and FAD^4T^ brains than in WT C57BL6 brains. And excessive GNG5 significantly aggravated deposition of Aβ plaques and Aβ42^+^ plaques in cortex (Fig. 3E, F) and hippocampus (Fig. S5G, H) of 5×FAD mice. Reducing GNG5 attenuated the Aβ plaques and Aβ42^+^ plaques deposition in cortex but not in hippocampus of 5×FAD mice. Results from FAD^4T^ brains confirmed the aforementioned findings (Figs. 3E, F and S5G, H). Quantitative detection revealed higher accumulation of Aβ42 and Aβ40 peptides in GNG5@EV^RVG^-treated cortex and hippocampus, and lower levels in siGNG5@EV^RVG^-treated cortex and hippocampus (Fig. 3G, H). The results were found in both 5×FAD and FAD^4T^ mice.

Considering the neurotoxic of Aβ peptides, then degeneration and neuron loss were determined in GNG5@EV^RVG^ and siGNG5@EV^RVG^-injected mice. Excessive GNG5 induced markedly higher neurodegeneration (FJC^+^) and neuronal loss (NeuN^+^) in the cortex than that in EV^RVG^-injected mice, whereas knockdown of GNG5 showed the opposite trend. (Fig. 3I, J).

In summary, these results indicate that excessive GNG5 exacerbates Aβ42 burden and causes neuronal damage in vivo.

### GNG5 leads to impaired spatial learning and increased anxiety-like behavior in 5×FAD and FAD^4T^ mice

**Fig. 4:**
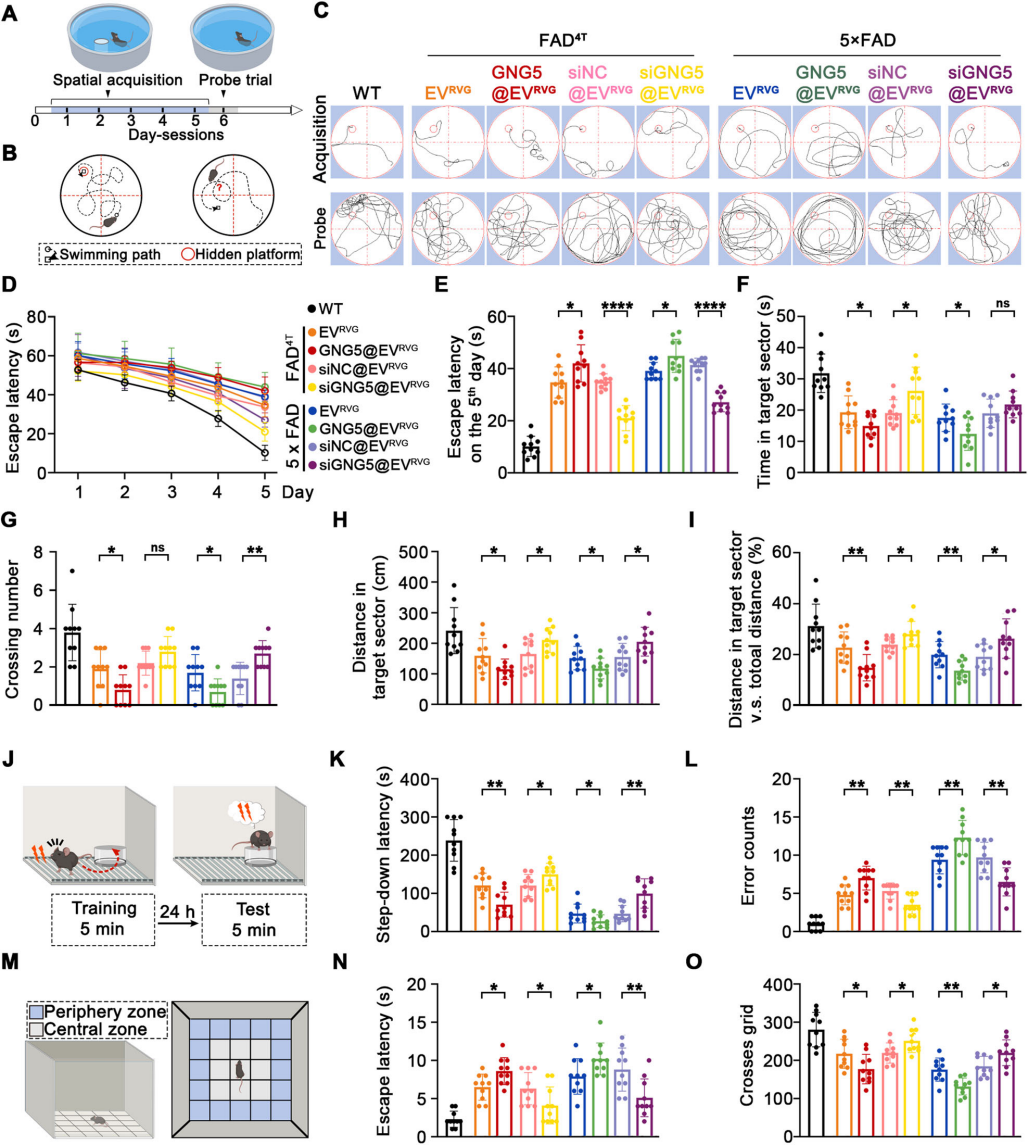
GNG5 aggravates learning and memory deficit in 5×FAD and FAD^4T^ mice. **A**, **B** Schematic diagram and behavioral outcomes of mice in the Morris water maze test. WT (female, 5-month-old, *n* = 10), 5×FAD (female, 5-month-old, *n* = 10 per group), FAD^4T^ (female, 4-month-old, *n* = 10 per group). **C** Representative swimming paths of acquisition on the fifth day and probe on the sixth day. Data from 5-day spatial reference memory training to reach the hidden platform (**D**) and on the fifth day of the spatial acquisition session (**E**). **F**, **G** Graphs showing time spent by mice in the target sector and the number of platform crossings in the probe test of the Morris water maze test. **H**, **I** Graphs showing the distance in the target sector and the proportion of distance in the target quadrant (%) during the probe trial. **J** Schematic diagram of the step-down passive avoidance test behavioral outcomes and the timeline of the training and the test session. WT (female, 5-month-old, *n* = 10), 5×FAD (female, 5-month-old, *n* = 10), FAD^4T^ (female, 4-month-old, *n* = 10). The time taken by the mice to step down (**K**) and the number of times the mice stepped down (**L**) from the platform onto the grid during the test session. **M** Schematic diagram of the open-field test behavioral outcomes. WT (female, 5-month-old, *n* = 10), 5×FAD (female, 5-month-old, *n* = 10), FAD^4T^ (female, 4-month-old, *n* = 10). Time spent mobile by the test mice from the central zone to the periphery zone (**N**) and the total number of grids traversed in 5 min (**O**). Data are presented as the mean ± SD. *p* values were determined using one-way ANOVA with Turkey post hoc test for multiple groups. * *p* < 0.05, ** *p* < 0.01, *** *p* < 0.001, ns: not significant.

Along with memory, the thinking ability and behavior of patients with AD are impaired because of the prolonged progressive degeneration of neurons [21]. Hence, a series of behavioral tests were conducted on 5×FAD and FAD^4T^ mice after a two-month intervention with GNG5@EV^RVG^ or siGNG5@EV^RVG^ to determine the impact of GNG5 on behavior (Fig. 3A). The Morris water maze (MWM) test showed that, on the sixth day, the swimming path of WT mice showed a pattern of target scanning, which focused on regions surrounding the platform. However, the trajectory pattern of 5×FAD and FAD^4T^ mice, particularly GNG5@EV^RVG^-injected 5×FAD and FAD^4T^, were characterized by thigmotaxis and random search (Fig. 4A, B, C). Latency to find the hidden platform was longer in GNG5@EV^RVG^-injected 5×FAD and FAD^4T^ mice than that in their respective control groups, while the siGNG5@EV^RVG^-injected 5×FAD and FAD^4T^ mice spent less time (Fig. 4C–E). In addition, compared with control groups, GNG5 overexpression led to markedly reduced values for time in the target quadrant, crossing number over the platform-site, distance in the target quadrant as well as the percentage of distance in target quadrant; siGNG5 downregulated these indicators (Fig. 4F–I). These data suggested that GNG5 exacerbated AD-related learning and memory defects.

In the step-down passive avoidance test, the step-down latency and error counts were used as measurements of memory retention in this study (Fig. 4J). Compared with the WT mice, the 5×FAD and FAD^4T^ mice exhibited a poor performance, represented by shorter latency and more errors (Fig. 4K, L). The GNG5@EV^RVG^-injected 5×FAD and FAD^4T^ mice showed an evidently shorter latency than that of the respective control group, while the siGNG5@EV^RVG^-injected 5×FAD and FAD^4T^ group showed contrasting results (Fig. 4K, L). Thus, GNG5 overexpression led to significantly elevated error counts, and siGNG5 restored the error counts to marginally lower levels than those of their respective control groups.

An open-field assay (Fig. 4M) demonstrated that the anxiety-like behaviors increased in 5×FAD and FAD^4T^ mice compared to those in the WT group, which reflected in longer latency and less crosses grid (Fig. 4N, O). GNG5@EV^RVG^-injected 5×FAD and FAD^4T^ mice showed higher anxiety-like behavior relative to their respective control groups, and siGNG5 partially rescued anxiety-like behavior in 5×FAD and FAD^4T^ mice (Fig. 4N, O).

Therefore, findings of the three independent memory-related behavioral tests provided evidence that increased GNG5 levels exacerbated performance in both short-term and long-term memory, as well as cognition, and reduced GNG5 levels ameliorated the behavioral deficiency of AD mice.

### GNG5 interacted with PS1 and promoted Aβ42 production

To elucidate the potential regulatory mechanism of GNG5 in Aβ42 production, GNG5 interacting proteins were then identified by IP-MS proteomics (Fig. S6A). Briefly, we isolated membrane proteins of 293T-GNG5^OE^ cells which stably overexpress human recombinant protein GNG5-FLAG, and enriched GNG5 interacting complexes with FLAG antibody (Fig. S6B). Six fractions were separated after sodium dodecyl sulfate-polyacrylamide gel electrophoresis (SDS-PAGE) (Fig. S6C). After in-gel digestion with trypsin/Lys-C respectively, LC-MS/MS detection was performed for six components. Numerous molecules, including essential elements for Aβ peptides production (APP, PS1, PS2, NCSTN), early endosome marker Rab5 isoforms, and GPCR-related proteins (GRK6, GNAI1, et al.) were found to coprecipitate with GNG5 (Fig. S6D). GO and WikiPathways analyses for these proteins unveiled the enrichment of proteins involved in the γ-secretase complex, β-amyloid formation, regulation of endocytosis, and G protein signaling pathways (Fig. S6E).

**Fig. 5:**
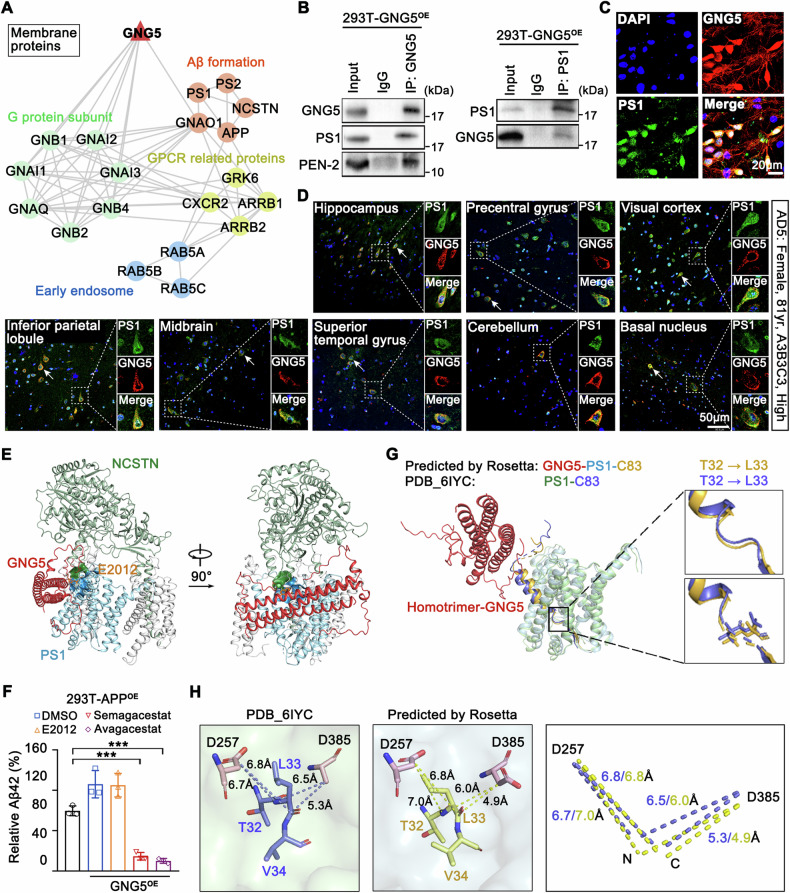
GNG5 interacted with PS1 and promoted Aβ42 production. **A** Protein–protein interaction network for the potential membrane proteins interacting with GNG5 identified in the IP/MS proteomics. The red triangle represents the hub GNG5. **B** Western blot detection of GNG5, PS1 and PEN2 (two γ-secretase subunits) in immunoprecipitation extracts from membrane proteins of 293T-GNG5^OE^ cells. Immunoprecipitation was performed with anti-GNG5 antibody or with anti-PS1 antibody. Immunofluorescence staining with anti-GNG5 (red) and anti-PS1 (green) antibodies in (**C**) primary hippocampal neurons from newborn rat (day 2) and in (**D**) eight brain regions related to A score (hippocampus, precentral gyrus, visual cortex, inferior parietal lobule, midbrain, superior temporal gyrus, cerebellum, and basal nucleus) from AD5 (Female, 81y, A3B3C3). Scale bar, 20 µm (**C**); scale bar, 50 µm (**D**). **E** Molecular docking models of γ-secretase-E2012 (PDB: 7D8X) and trimeric GNG5 predicted using ZDOCK. **F** Quantification of Aβ42 in cellular supernatant obtained from 293T-APP^OE^-GNG5^OE^ cells, pretreated with the γ-secretase inhibitors E2012, Semagacestat, or Avagacestat. **G**, **H** Structural models of γ-secretase-C83 complex with homotrimer-GNG5 constructed using Rosetta. GNG5-free PS1–C83 was derived from Cryo-EM structure (PDB: 6IYC). **G** Enlarged view depicts comparison of positional shift of residues Thr32 and Leu33 between GNG5-bound and GNG5-free PS1–C83. **H** A close view of the predicted distance between the carboxylate side-chain of Asp257 or Asp385 of PS1 and the C=O group or NH group of Leu33 of C83 in GNG5-free PS1–C83 and GNG5-bound PS1–C83 structures. Residues Thr32, Leu33, Val34 in C83 correspond to Thr48, Leu49, Val50 in C99. Data are presented as the mean ± SD. *p* values were determined using one-way ANOVA with Turkey post hoc test for multiple groups. **p < 0.01.

Protein-protein interaction reveals core protein of GNG5 with close interaction with γ-secretase subunits, early endosomal markers, and GPCR related proteins (Fig. 5A). Considering that GNG5 can bind to PS1 (Fig. 2), we performed immunoprecipitation and validated the interaction of GNG5 with PS1 at the cell membrane (Fig. 5B). Immunofluorescence staining in primary hippocampal neurons supported this finding (Fig. 5C). To ascertain the interaction between GNG5 and PS1 in pathophysiological conditions, we stained paraffin sections of eight brain regions from two postmortem brains diagnosed as AD with “H” pathological level (A3B3C3) (Figs. 5D and S7A), and hippocampal–entorhinal paraffin sections from six NC and two pathological AD donors (Fig. S7B). The results confirmed the co-localization of GNG5 and PS1, suggesting their interaction in pathophysiological conditions.

Furthermore, we constructed a three-dimensional (3D) structural model of γ-secretase (PDB: 7D8X) in complex with homotrimer-GNG5 using ZDOCK, aiming to predict the involvement of GNG5 in γ-secretase activity. From the structure perspective, it suggested the possible direct binging of GNG5 at the interface between PS1 and NCSTN (Fig. 5E), which may hinder E2012 inserting into the hydrophobic pocket formed by PS1 and NCSTN residues [14]. The prediction was evidenced by incubation of γ-secretase inhibitors (Fig. S8) with 293T-GNG5^OE^. GNG5 overexpression elevated Aβ42 generation. Semagacestat and Avagacestat [14], which binding to PS1 active site, remained strong inhibit ability with sharply reduce of Aβ42 production. While E2012 lost inhibitory ability with GNG5 overexpression (Fig. 5F). These results were consistent with structure prediction and that in Fig. 2K, suggesting that GNG5 does not directly interact with the cleavage pocket of PS1.

A 3D structural model of γ-secretase–C83 complex (PDB: 6IYC) and GNG5 was constructed using Rosetta. The overall structure of GNG5-bound PS1–C83 was superimposed on that of GNG5-free PS1–C83 and compared. As reported, Aβ42 and Aβ40 are derived from the cleavage of Aβ48 and Aβ49, respectively, by γ-secretase with its *C*-terminal peptidase activity [22, 23]. A close view of the structure indicated positional shifts in residues Thr32 and Leu33 (that are Thr48 and Leu49 in C99) in GNG5-bound PS1–C83 (Fig. 5G, H).

### The interaction between GNG5 and PS1 to promote Aβ42 production can occur in the early endosome

**Fig. 6:**
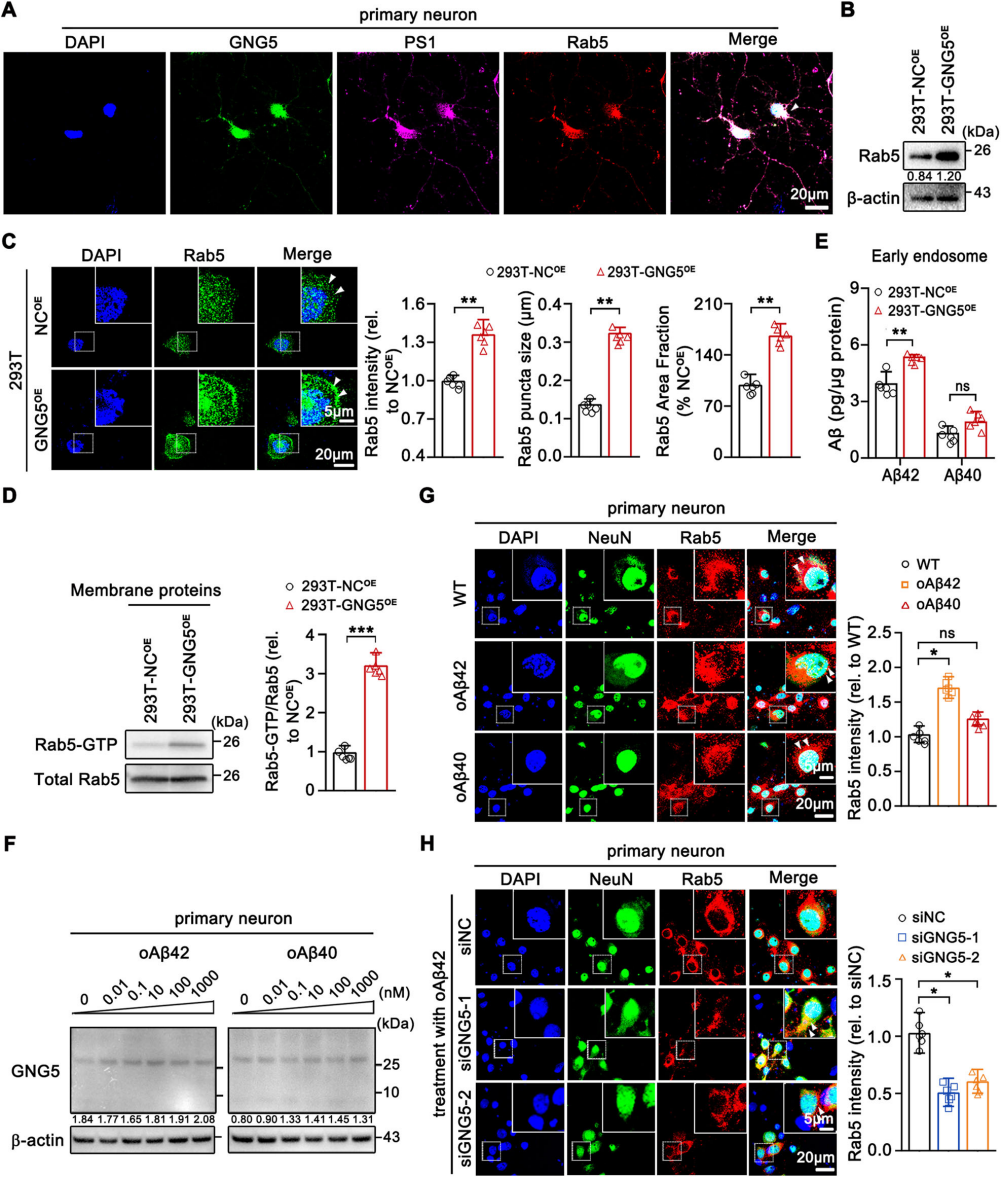
The interaction between GNG5 and PS1 promoting Aβ42 production can occur in early endosomes. **A** Representative confocal images for the co-localization of Rab5 with GNG5 and PS1 in primary hippocampal neurons. Scale bar, 20 µm. **B** Western blot analysis of Rab5 in 293T-NC^OE^ and 293T-GNG5^OE^. **C** Confocal microscopic staining of Rab5 in 293T-NC^OE^ and 293T-GNG5^OE^ cells. Statistical analyses for Rab5 intensity, puncta size, and area fraction are shown. Scale bar, 20 µm; enlarged, 5 µm. **D** Representative blots of Rab5 activation in membrane protein derived from 293T-NC^OE^ and 293T-GNG5^OE^ cells by GTP-agarose pull-down and detection using a Rab5 antibody. Rab5 activation is expressed as Rab5–GTP/total Rab5. **E** Extraction of early endosomes from 293T-NC^OE^ and 293T-GNG5^OE^ cells using commercial kits, followed by the ELISA quantification of Aβ42 and Aβ40. **F**, **G** Primary hippocampal neurons was treated with oAβ42 or oAβ40 at varied concentration (0.01, 0.1, 10, 100, or 1000 nM). Western blot detection of GNG5 (**F**) and immunofluorescence imaging of Rab5 (**G**) in treated cells. Scale bar, 20 µm; enlarged, 5 µm. **H** Immunofluorescence imaging of Rab5 in oAβ42-stimulated primary hippocampal neuron model transfected with non-targeting negative control siNC or siGNG5. Scale bar, 20 µm; enlarged, 5 µm. Data are presented as the mean ± SD. *p* values were determined using unpaired two-tailed Student’s *t*-test for two groups and one-way ANOVA with Turkey post hoc test for multiple groups. *p < 0.05, **p < 0.01, ***p < 0.001, ns: not significant.

Previous researches have demonstrated relationship of early endosome and Aβ production [24]. Small GTPase Ras-related protein Rab5 is necessary for the biogenesis of endocytic pathway [9]. Rab5 transfected cells exhibited abnormally large endosomes and increased Aβ production [24]. Our prior combined analysis of hippocampal-entorhinal proteomics with IP-endosomal proteomics (Fig. 1E) and IP/MS analysis (Fig. 5A) suggested involvement of GNG5 in early endosome. Immunostaining on primary hippocampal neurons (Fig. 6A) and 293T cells (Fig. S9) demonstrated the co-localization of PS1 and GNG5 with Rab5, suggesting possibility of affected γ-secretase cleavage activity by GNG5 in early endosome.

To investigate this propose, we firstly confirmed total Rab5 levels and western blot revealed that overexpressed GNG5 elevated Rab5 protein level and vice versa (Figs. 6B and S10G). Immunofluorescence verified this finding, with markedly higher Rab5 intensities in GNG5 overexpressed 293T cells (Fig. 6C), primary hippocampal neurons (Fig. S10B, C), and SH-SY5Y cells (Fig. S10D) than those in their respective controls. *Rab5* mRNA levels were not significantly affected by GNG5 (Fig. S10A, F), indicating Rab5 was regulated by GNG5 post-translationally.

From the immunofluorescence images, GNG5 overexpression significantly increased Rab5 localization to the plasma membrane in 293T (Fig. 6C). Downregulation of GNG5 alleviated Rab5 accumulation on plasma membrane (Fig. S10H). This effect may be because Rab5 switches between an inactive GDP-bound (Rab-GDP) state in the cytosol and an active GTP-bound (Rab-GTP) state which is recruited to the cell membrane [25, 26]. Thus, membrane proteins from 293T-GNG5^OE^ were extracted, and the levels of activated, GTP-bound Rab5 were determined by a Rab5-GTP-agarose pull-down assay followed by detection with anti-Rab5 antibody. There was an approximately threefold increase of Rab5-GTP in 293T-GNG5^OE^ compared with 293T-NC^OE^ (Fig. 6D).

As reported, pathological Rab5 overactivation mediated endosome enlargement accompanying accelerated endocytosis and fusion [10, 11], and aberrant signaling by endosomes, and is a unifying cytopathological hallmark of AD [12, 13]. We next determined morphological alteration of Rab5 endosomes by IF labeling in 293T-GNG5^OE^, SH-SY5Y-GNG5^OE^, and rat primary hippocampal neurons overexpressing GNG5 with various strategies, including GNG5 lentivirus transfection, human brain-derived EV incubation, engineered GNG5@EV^RVG^ incubation, using anti-Rab5 antibody. The results showed that GNG5 overexpression led to an approximately twofold increase in the size of Rab5^+^ puncta and in the proportion of the cell area covered (Figs. 6C and S10C, D). Knockdown of GNG5 in 293T-GNG5^OE^ cells largely reversed the upregulation of Rab5 protein and attenuated Rab5^+^ puncta size and area fraction (Fig. S10H). These results were in accordance with expected Rab5-GTP-mediated enhancement of homotypic Rab5-endosome fusion by GNG5 and resemble the previously reported pattern of enlarged early endosomal labeling in AD brains [8].

Subsequently, we isolated the early endosome fractions using commercial kit (Invent ED-028) from 293T cells. Enhanced γ-secretase activity was verified with 1.4-fold more production of Aβ42 peptide in early endosome, and no influence on Aβ40 production (Fig. 6E). This effect was not detected in Golgi apparatus fractions, another site for Aβ production (Fig. S10E).

We further used Aβ to stimulate primary hippocampal neurons, 293T, and SH-SY5Y cells. Intriguingly, Aβ42 oligomers (oAβ42) stimulation (0.01–1000 nM) increased the levels of homotrimer-GNG5 in the primary hippocampal neurons (Fig. 6F) but did not affect *GNG5* mRNA levels (Fig. S11A). Aβ40 oligomers (oAβ40) at the same concentrations as oAβ42 affected neither the protein nor the transcription level of GNG5 (Figs. 6F and S11A). In SH-SY5Y cells, oAβ42 treatment or higher concentrations of oAβ40 induced an increase in the homotrimer-GNG5 levels without affecting the *GNG5* mRNA level (Fig. S11B, C). In 293T cells, not the oAβ42 but the Aβ42 monomer (mAβ42) at high concentrations (5 or 10 μM) and oAβ40 induced an upward trend of homotrimer-GNG5 without affecting its transcription levels (Fig. S11D, E, F). We subsequently determined Rab5 expression changes in Aβ-stimulated cell models. Consistently, Rab5 protein levels were significantly increased in Aβ-stimulated cells (Figs. 6G and S12A, B). To confirm that elevated Rab5 protein levels were induced by increased GNG5 expression, we knocked-down GNG5 in the Aβ-stimulated cell models and observed a significant reduction in Rab5 protein levels in primary hippocampal neurons (Fig. 6H), 293T (Fig. S12C) cells, and SH-SY5Y (Fig. S12D) cells.

These results collectively suggested that overexpressed GNG5 recruited more activated Rab5-GTP to plasma membrane, promoted endocytosis and fission of early endosomes, and greatly enhanced Aβ42 production in early endosomes. Besides, the results presented above suggested that GNG5 can enhance Aβ42 generation through Rab5 activation, in turn, Aβ42 increases GNG5 protein levels, thereby creating a feedback loop that exacerbates Aβ pathology.

### Excessive Aβ42 can upregulate GNG5 protein levels via CXCR2

**Fig. 7:**
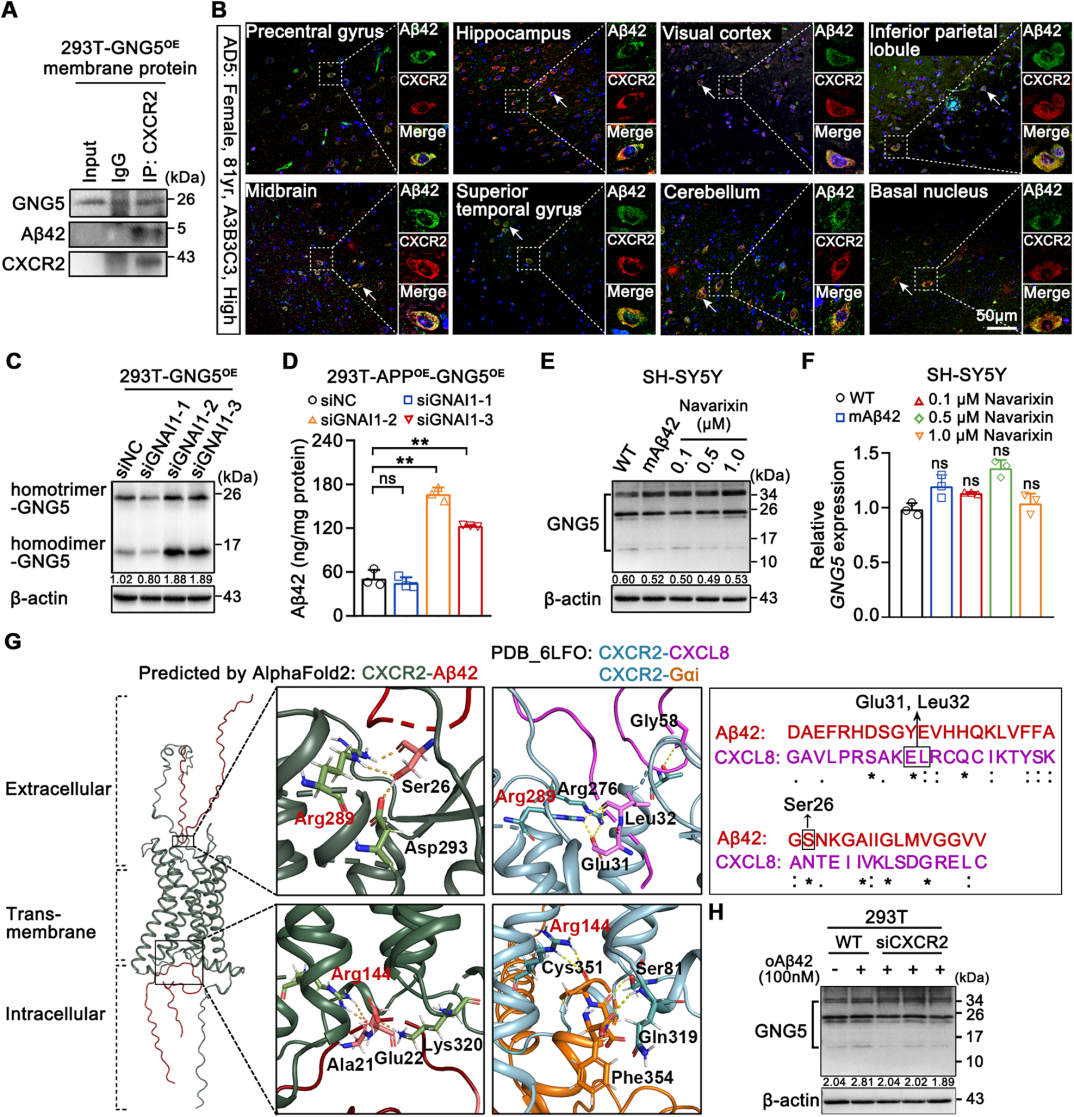
Aβ42 regulates GNG5 protein by interacting with CXCR2. **A** Immunoprecipitation with anti-CXCR2 from 293T-GNG5^OE^ membrane proteins. Western blot detected the presence of GNG5, Aβ42 and CXCR2. **B** Double staining with anti-CXCR2 (red; Proteintech, cat #20634-1-AP) and anti-β amyloid 1-42 (green; Cell Signaling Technology, cat #14974) in eight brain regions (precentral gyrus, hippocampus, superior temporal gyrus, inferior parietal lobule, visual cortex, basal nucleus, cerebellum, and midbrain) from donor AD5. Apparent co-localization between CXCR2 and Aβ42 in yellow. Scale bar, 50 μm. **C** Western blot detection of GNG5 in 293T-GNG5^OE^ cells with GNAI1 knockdown. **D** ELISA quantification of Aβ42 levels in 293T-APP^OE^-GNG5^OE^ cells with GNAI1 knockdown. **E**, **F** Western blot and qRT-PCR detection of GNG5 in SH-SY5Y cells treated with mAβ42 or different concentrations (0.1, 0.5, 1.0 µM) of the CXCR2 antagonist Navarixin. **G** Molecular docking of CXCR2 with two Aβ42 molecules using AlphaFold2, and the enlarged views predicting extracellular and intracellular binding sites for Aβ42 and CXCR2. The resolved binding sites of extracellular CXCL8 and intracellular Gαi with CXCR2 are also shown. “*,” “**:**,” and “.” indicate amino acid residues with full identity, strong similarity, and weak similarity, respectively. **H** Western blot detection of GNG5 in oAβ42-stimulated 293T with CXCR2 knockdown. Data are presented as the mean ± SD. *p* values were determined using one-way ANOVA with Turkey post hoc test for multiple groups. **p < 0.01, ns: not significant.

Next, we elucidated the molecular mechanisms underlying the GNG5 elevation by Aβ42. As indicated in Fig. 5A, IP-MS results identified co-interaction of G-protein coupled receptor (GPCR) related proteins with GNG5, including C-X-C chemokine receptor type 2 (CXCR2), G-protein-coupled receptor kinase 6 (GRK6), and β-arrestin (ARRB1 and ARRB2). As previous observations, GRK6 is a kinase of CXCR2, one of the GPCRs, and phosphorylates serines and threonines in receptor *C* tail. Phosphorylated GPCR activation enhanced binding with β-arrestin (ARRB1 and ARRB2) and initiates β-arrestin-mediated signaling pathways, thus leading to GPCR desensitivity, which reduced or slack off coupling with G proteins. Thus, we proposed that whether Aβ42 upregulated GNG5 protein level through CXCR2, a member of the most widely used GPCR family of drug targets. To verify this propose, immunoprecipitation with anti-CXCR2 antibody provided evidence for the interaction between GNG5 and CXCR2 (Fig. 7A). Besides, Aβ42 was found co-precipitated with GNG5 and CXCR2. Immunostaining on paraffin sections of eight brain regions from two postmortem diagnosed “H” pathological level (A3B3C3) brains (Figs. 7B and S13A), and on hippocampal–entorhinal paraffin sections from six NC and two AD donors (Fig. S13B), confirmed the co-localization of Aβ42 and CXCR2. Therefore, these results provided molecular basis of interactions of these components.

Then, we explored the relationship between GNG5 and CXCR2. In 2020, Liu and colleagues resolved three-dimensional (3D) structures (PDB code: 6LFO) of interleukin-8 (CXCL8)-activated human CXCR2 in complex with trimeric G proteins (GNAI1-GNB1-GNG2, Gα_i_-Gβ-Gγ). Considering high sequence similarity (48% pairwise sequence identity) between GNG2 and GNG5 calculated by JALVIEW (Fig. S14A), we predicted the 3D structure of CXCL8-activated human CXCR2 with trimeric GNAI1-GNB1-GNG5 using computational approach by AlphaFold2 (Fig. S14B). Through superposition of the 3D structures, we found almost coincidence of tertiary structure features between GNG2 and GNG5. Therefore, GNG5 could assemble trimeric G proteins and couple to activated CXCR2.

Ligand binding results in CXCR2 activation and coupling to Gαi, with concomitant dissociation of Gβγ from Gαi [27, 28]. Consistent with our hypothesis, the knockdown of GNAI1 from 293T-GNG5^OE^ cells (Fig. S15A) significantly increased the homodimer- and homotrimer-GNG5 levels (Fig. 7C), while the mRNA levels of GNG5 remained unchanged (Fig. S15B). Significantly increased Aβ42 levels was found in GNAI1 knockdown 293T-APP^OE^-GNG5^OE^ cells (Fig. 7D). That is, without uncoupling to CXCR2, GNG5 stably exists as oligomers. While the knockdown of GNAO1, another Gα subfamily member, did not markedly alter GNG5 protein or mRNA levels (Fig. S15C, D), indicating GNG5 protein levels being Gαi dependent. Navarixin is a representative CXCR2 antagonist that binds to its orthosteric pocket and hinders coupling of Gα_i_ with CXCR2 [25]. In SH-SY5Y cells, treatment with navarixin at varied concentrations (0.1, 0.5, and 1.0 μM) promoted increase of oligomeric GNG5 expression levels (Fig. 7E). Low concentrations of mAβ42 did not increase GNG5 protein levels, and neither Navarixin nor mAβ42 perturb the transcription of GNG5 (Fig. 7F). These observations suggested that GNG5 assembly to heterotrimeric G proteins mainly with Gαi and Gβ, little with Gαo.

Next, we predicted the binding between CXCR2 and Aβ42 peptide with amino acids sequence using AlphaFold2, and subsequently performed the molecular docking of CXCR2 with one, two, or three Aβ42 molecules. A single Aβ42 molecule bound at the extracellular *C*-terminus of CXCR2 (Fig. S14C), while two Aβ42 molecules bound at both the termini, the extracellular and intracellular (Fig. 7G). The third Aβ42 molecule did not bind (Fig. S14C).

CXCR2 is the receptor for interleukin 8 (CXCL8). From the structures of CXCL8-activated human CXCR2 in complex with trimeric G proteins (PDB code: 6LFO), we showed the resolved binding sites of extracellular CXCL8 and found that CXCL8 binds to the residue Arg289 of CXCR2 through residue Glu31 and Leu32. Sequence alignment of Aβ42 and CXCL8 protein revealed two highly similar sequences between Aβ42 and CXCL8, which included the above CXCR2-binding amino acid residues (Fig. 7G, upper). Hence, we infer that Aβ42 could bind to the extracellular region of CXCR2, leading to CXCR2 activation and coupling to Gα [27, 28], with GNG5 release from Gα.

In addition, detailed structural comparison between CXCR2-Aβ42 and CXCR2-Gαi revealed overlapping of Aβ42 with Gαi at the interface with CXCR2. The resolved binding sites of intracellular Gαi (PDB: 6LFO) showed that Gαi binds to Arg144 of CXCR2 through Cys351, and Aβ42 binds to Arg144 of CXCR2 through Ala21, indicating that Aβ42 competitively binds to the intracellular region of CXCR2 with Gαi (Fig. 7G, lower) [26]. Thus, we hypothesized that Aβ42 could also bind to the intracellular region of CXCR2 and cause GNG5 to dissociate from CXCR2.

Totally, the computational study provided structure basis for relationships of Aβ42, CXCR2, and G proteins. Consistent with our structure study, knockdown of CXCR2 (Fig. S15E) in oAβ42-stimulated 293T cells restored the enhancement of GNG5 proteins to the level in WT group (Fig. 7H) without altering the mRNA levels (Fig. S15F). In addition, IF with Aβ42 demonstrated that GNG5 overexpression significantly increased Aβ42 production relative to the 293T-APP^OE^ group. While knockdown of CXCR2 in 293T-APP^OE^-GNG5^OE^ cells did not significantly affect Aβ42 production (Fig. S15G), indicating that GNG5 did not reversely regulate CXCR2 for Aβ42 production.

In addition, we found no significant differences in CXCR2 levels in the brain tissue EVs between NC and pathological AD donors, which dismisses the possibility that the variations observed in our results are attributable to the differential expression of CXCR2 under pathophysiological conditions (Fig. S15H).

In summary, Aβ42 increased GNG5 protein levels may by binding to the extracellular or the intracellular pocket of CXCR2, thus leading to the dissociation of GNG5 from Gαi and the formation of oligomeric GNG5 forms.

### GNG5 positively correlates with AD neuropathologic changes and is a potential biomarker in serum EVs

**Fig. 8:**
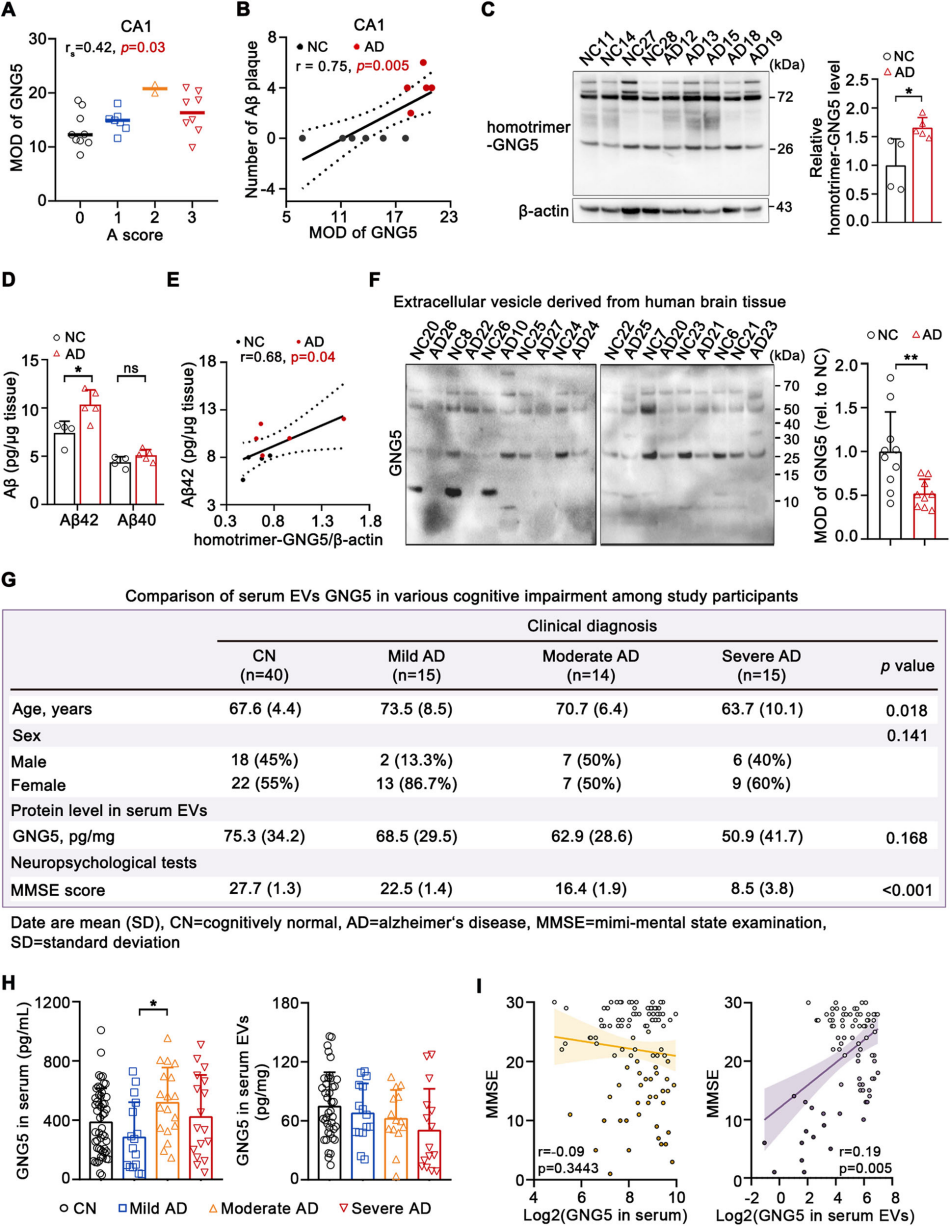
The potential clinical significance of GNG5 in neuropathologic changes and serum extracellular vesicle diagnosis. **A** Relative expression of GNG5 with different A scores in the CA1 subregion. Median values are represented by lines. Related to Fig. S3A, r_s_: Spearman correlation coefficient. A0 (*n* = 9, 81.2 ± 9.6 yr), A1 (*n* = 7, 91.3 ± 5.1 yr), A2 (*n* = 2, 88.5 ± 3.5 yr), A3 (*n* = 8, 88.8 ± 5.5 yr). **B** Linear correlation between GNG5 levels determined using IHC and the number of Aβ plaques in the CA1 subregion, related to Fig. S16B. NC (*n* = 6, 81.7 ± 10.6 yr), AD (*n* = 5, 90.3 ± 4.7 yr). **C** Western blot detection and densitometry analysis of GNG5 levels in the CA1 subregion. NC (*n* = 4, 87.3 ± 5.1 yr), AD (*n* = 5, 90.4 ± 9.5 yr). **D** ELISA quantification of Aβ42 and Aβ40 levels in radioimmunoprecipitation assay buffer-soluble proteins from human hippocampal CA1 tissues. NC (*n* = 4, 87.3 ± 5.1 yr), AD (*n* = 5, 90.4 ± 9.5 yr), the same samples as in Fig. 8C. **E** Pearson correlation analyses between trimeric GNG5 levels (**C**) and Aβ42 levels (**D**). **F** Western blot detection and densitometry analyses of GNG5 in EVs derived from the cerebral cortex. NC (*n* = 10, 82.3 ± 5.3 yr), AD (*n* = 9, 82.4 ± 5.3 yr). **G** Characteristics of study participants and the analysis of GNG5 levels in serum EVs. **H** ELISA quantification of GNG5 levels in serum (CN, *n* = 52; Mild AD, *n* = 16; Moderate AD, *n* = 18; Severe AD, *n* = 17) or serum EVs (CN, *n* = 40; Mild AD, *n* = 15; Moderate AD, *n* = 14; Severe AD, *n* = 15). **I** Scatter plots of mini-mental state examination (MMSE) and GNG5 levels in serum or in serum EVs. Data are presented as the mean ± SD. *p* values were determined using unpaired two-tailed Student’s *t*-test for two groups and one-way ANOVA with Turkey post hoc test for multiple groups. *p < 0.05, **p < 0.01, ns: not significant.

To ascertain the clinical relevance of increased GNG5 expression, our initial investigation focused on elucidating the correlation between heightened GNG5 levels in the brain tissue and the severity of neuropathological changes. Correlation analyses between GNG5 and the A, B, and C scores showed that GNG5 protein levels in the CA1 and CA4 regions positively correlated with the A score (Figs. 8A and S16A). Moreover, IHC analysis substantiated a positive correlation between GNG5 expression and the Aβ plaque content in the CA1 region (Figs. 8B and S16B). Western blot results supported increased GNG5 protein levels in the hippocampal CA1 subregion of pathological AD donors (Fig. 8C). Interestingly, we observed an ~25 kDa band, with size consistent with homotrimer–GNG5 (Fig. 8C), suggesting that GNG5 might exist in human brain tissue in a trimeric isoform, which was consistent with findings in Fig. S3B. Furthermore, the additional bands at ≥70 kDa might represent homogeneous protein complexes formed by GNG5, or heterogeneous protein complexes formed by GNG5 and other G protein subunits.

Subsequently, we quantified the levels of Aβ42 and Aβ40 in the human brain tissue samples used in Fig. 8C and performed a correlation analysis on GNG5 protein levels and the contents of Aβ42 or Aβ40. Soluble Aβ42 levels in the brain tissues of pathological AD were significantly increased, while Aβ40 levels showed no significant change (Fig. 8D). GNG5 expression positively correlated with the Aβ42 content (Fig. 8E) but demonstrated no significant correlation with the Aβ40 content (Fig. S16C). Interestingly, GNG5 levels were significantly reduced in EVs derived from the brain tissues of pathological AD compared to those in the NC group (Fig. 8F). Thus, GNG5 expression may be associated with Aβ42 production and the formation of Aβ plaques in pathophysiological conditions.

A total of 52 cognitively normal participants (aged 65.7 ± 5.6 years), 17 patients with severe AD (aged 63.8 ± 9.7 years), 18 patients with moderate AD (aged 71.2 ± 7.9 years), and 16 patients with mild AD (aged 73.3 ± 8.3 years) were included in this study, to evaluate the variation in serum GNG5 levels at different clinical stages of AD (Fig. S16D). In addition, a total of 40 cognitively normal participants (aged 67.6 ± 4.4 years), 15 patients with severe AD (aged 63.7 ± 10.1 years), 14 patients with moderate AD (aged 70.7 ± 6.4 years), and 15 patients with mild AD (aged 73.5 ± 8.5 years) were used to evaluate the variation in GNG5 level in serum EVs at different clinical stages of AD (Fig. 8G). There was no significant difference in sex composition ratio (*p* = 0.390, *p* = 0.141) among the groups. The serum GNG5 levels in mild AD (average 289.9 ± 231.6 pg/mL) were significantly lower than those in moderate AD (average 524.0 ± 231.6, *p* < 0.05), with no significant differences between the other groups. GNG5 in serum EVs showed a non-significant, gradually decreasing trend in the CN (average 75.3 ± 34.2 pg/mg), mild AD (average 68.5 ± 29.5 pg/mg), moderate AD (average 62.9 ± 28.6 pg/mg), and severe AD (average 50.9 ± 41.7 pg/mg) (Fig. 8H). To further explain the variation of GNG5 in EVs of AD patients, we enriched neuron-derived extracellular vesicles (NDEVs) from 5 cognitively normal participants (CN, aged, 86.1 ± 6.3 yr), and 5 patients with AD (aged, 88.4 ± 7.4 yr). The WB results indicate that GNG5 levels are significantly reduced in NDEVs from patients with AD (Fig. S16E). And, a significant positive correlation was observed between GNG5 levels in serum EVs and mini-mental state examination (MMSE) scores (Fig. 8I).

The above results indicated that GNG5 levels in the human brain positively correlated with Aβ pathology. Furthermore, GNG5 found in serum EVs may exhibit significant clinical relevance in distinguishing between CN and AD, potentially serving as a viable biomarker for the early diagnosis of AD.

## Discussion

To date, therapies targeting Aβ and tau proteins are the predominant treatments for AD, accounting for ~30% of AD drug development [29]. The FDA has approved six drugs for Aβ clearance. Crucially, these drugs merely alleviate symptoms without altering the disease course. Additionally, only three monoclonal drugs, aducanumab, lecanemab and donanemab, have emerged from 38 discontinued drugs because of ineffectiveness and toxic side effects; their efficacy remains under observation [30]. In contrast, γ-secretase inhibitors have been extensively researched as potential AD treatments because of their ability to inhibit Aβ production [31]. However, Eli Lilly’s small-molecule γ-secretase inhibitor, semagacestat, faced failure in Phase 3 clinical trials because of adverse events such as skin cancer and infections [4]. Meanwhile, gamma-secretase modulators, such as SGSM-36 and EVP-0962, targeting the same enzyme, successfully reduce levels of the toxic Aβ42 peptide; however, their use has also ceased in clinical trials [32, 33]. Moreover, to date, no tau-targeted treatments have shown definitive clinical efficacy in the preclinical or early stages of AD. This trend is expected to continue, as the monoclonal antibody Gosuranemab, aimed at the N-terminal tau protein in patients with early-stage AD, failed to exhibit significant results in cognitive and functional assessments [5]. Therefore, considering the limited therapeutic options for AD, there is an urgent need to identify genes that modulate the early formation of Aβ peptides, particularly Aβ42, and to inhibit oligomers derived from Aβ42.

The findings of this study indicate that GNG5 upregulation promotes the upregulation and activation of Rab5, which facilitates the production of Aβ42 by recruiting Rab5 to the membrane, leading to a significant increase in the number of early endosomes. In the in vivo experiments, reducing GNG5 decreased Aβ42 production and improved cognitive impairment in mice, suggesting that GNG5 could serve as a potential target for AD intervention. Considering the advantages of EVs with high physicochemical stability and biocompatibility, low toxicity, and immunogenicity [34], using engineered EVs to deliver siRNA targeting GNG5 may represent a novel approach for AD intervention, distinct from direct Aβ clearance and γ-secretase inhibition.

Notably, in the mechanism elucidated in this study, overactivated Rab5 can accelerate the transport and recycling of early endosomes. However, Rab GTPases are inefficient enzymes with a low intrinsic GTP hydrolysis rate and are thus dependent on GTPase activating proteins (GAPs) to hydrolyze bound GTP [35, 36]. Our data showed that GNG5 significantly promoted Rab5 activation; hence, we speculate that GNG5 may function as a GAP to accelerate the activation cycle of Rab5. The above data potentially account for the interesting outcomes in Fig. 8F, wherein the heightened use of GNG5, because of a rapid endocytosis rate and activation cycling, leads to a notable decrease in GNG5 transported by EVs to the extracellular space.

Aβ is reported to be a ligand for TREM2 in microglia [37], to the best of our knowledge, our research is the first to suggest that Aβ42 oligomers act as a ligand for CXCR2 in neuronal cells. Aβ42 binding activate CXCR2, facilitating the dissociation of G-proteins βγ from α subunits, activating downstream signaling pathways. Protein structure predictions suggest that Aβ42 can bind to both extracellular and intracellular regions of CXCR2. which worth further in-depth exploration. CXCR2 is the receptor for CXCL8. The structures prediction and sequence alignment indicate that Aβ42 and CXCL8 competitively binds to the CXCR2 extracellular pocket. Thus CXCL8 could hinder the binding of Aβ42 to CXCR2, which is consistent with previous report that CXCL8 protects human neurons from Aβ-induced neurotoxicity [38].

In addition, humans express four distinct Gα subunits (Gαo, Gαi, Gαs, and Gαq), five Gβ subunits along with their variants (β1, β2, β3, β3S, β4, β5, and β5L), and 12 Gγ proteins (Gγ1–5 and 7–13), which can potentially combine to form various heterotrimeric combinations in the cell membrane [39–42]. Gβγ essentially functions as a single protein complex. Individual Gβ or Gγ subunit is unstable, and thus require dimerization to effectively perform their physiological functions [43]. To our knowledge, this study reveals for the first time that GNG5 exists as an independent homotrimer in the brain tissue and acts in this form as a hidden regulator of Aβ42 production. The molecular weights of Gα (o, i, s, q), Gβ (1–5), and Gγ (1–5, 7–13) subunits have been explicitly stated as 34–45, 36, and 7–11 kDa, respectively [39, 41], which excludes the possibility that bands ≤34 kDa are complexes formed by GNG5 with α or β. Thus, we propose that GNG5 in the human brain can spontaneously assemble functional homo-oligomers, whose abnormal elevation leads to a significant increase in Aβ42 production, exacerbating AD pathology. This finding also implies that the β subunits and γ subunits might independently exhibit molecular biological functions. This insight provides a fresh perspective for investigating G-proteins.

We propose that GNG5 is the upstream regulator of Aβ42 in the mechanism elucidated in this study. Specifically, the elevated GNG5 levels in patients with AD lead to increased Aβ42 production. The augmented Aβ, in turn, further promote the dissociation of GNG5 from CXCR2, ultimately resulting in a vicious cycle and exacerbation of Aβ pathology. Age is the greatest risk factor for AD [44]. The data of brain tissue EVs showed that GNG5 levels increased with age in both healthy and AD groups. However, this increase is more gradual in AD, although the difference was not statistically significant because of limited sample size (Fig. S17A). This finding suggests that aging exacerbates individual differences in the EV-mediated clearance of GNG5 in the brain. This metabolic discrepancy may lead to an accumulation of GNG5 in the brains of some individuals, causing elevated Aβ42 levels. This conclusion is further substantiated by our findings in three AD mouse models: APPswe/PSEN1dE9, FAD^4T^, and 5×FAD. These models demonstrated a definite elevation in GNG5 levels within brain tissues, which was associated with more pronounced Aβ deposition and heightened cognitive deficits (Figs. 3, 4). Conversely, no significant variation in GNG5 expression was detected in the brain tissues of young adult APPswe/PSEN1dE9 mice (two-month-old) that lacked Aβ plaque formation (Fig. S17B, C). Therefore, both human brain tissue and animal model results indicate that GNG5 is the upstream regulator of Aβ42.

The reduction of GNG5 in serum-derived EVs holds potential for the early diagnosis of AD. A large body of literature supports the potential of serum-derived EVs in the diagnosis of tumors, neurodegenerative diseases, and cardiovascular diseases [45–47]. The diverse origins of serum-derived EVs from multiple organs and tissues contribute to their heterogeneity. The relatively low abundance of EVs from specific cell types further makes it challenging to isolate serum-derived EVs from distinct cellular sources and utilize them for disease diagnosis. Zhou et al. highlighted the urgent need to identify specific EV subtypes [48]. Developing well-established strategies to identify and isolate specific EV subtypes would advance EVs’ broad clinical applications. While, in this study, our data demonstrated that the reduction of GNG5 in neuron-derived EVs in AD patients (Fig. S16E) is consistent with the reduction of GNG5 observed in total serum-derived EVs in AD (Fig. 8H). This suggests that detecting the reduction of GNG5 in serum-derived EVs could distinguish AD from CN and has the potential for early AD diagnosis without the need for isolation and enrichment of neuron-derived EVs.

In conclusion, our findings reveal that GNG5 upregulates Aβ42 production by directly interacting with the PS1 subunit of γ-secretase. As a ligand of CXCR2, Aβ42 oligomers induce GNG5 to separate from CXCR2 and recruit Rab5’s upper membrane, which promotes early endosome formation and further upregulates Aβ42 production, forming a vicious cycle and aggravating Aβ pathology. Considering a significant reduction in GNG5 in brain-derived EVs and serum-derived EVs from patients with AD, we suggest that GNG5 is a novel regulator of Aβ42 and a potential early diagnosis biomarker and drug target for AD.

### Limitations of this study

The high accuracy of AlphaFold is an important milestone in the field of protein structure prediction [49]. Here, we used the AlphaFold2 dataset to predict the structures of GNG5, Aβ42, and CXCR2. We also attempted to explore the potential involvement of GNG5 in PS1-mediated cleavage of substrate C83, leading to the preferential production of Aβ42, as well as the possible molecular mechanisms through which Aβ42 feeds-back to upregulate GNG5 protein, using ZDOCK or Rosetta. Nevertheless, to confirm the conclusion that GNG5 acts as a novel regulator and to provide information for drug target research, cryo-electron microscopy analysis of the homotrimer-GNG5–γ-secretase complex structure may still be required.

## Material and methods

### Key resources table

The key resources table is attached in supplementary Table S4.

## Experimental model and patient details

### Animals

Two-month-old female APPswe/PSEN1dE9 mice carrying human APP with the Swedish mutation and human PSEN1 lacking exon 9 (dE9) were used in this study [50]. Five-month-old female 5×FAD transgenic mice carrying five human mutations, with three mutations in *APP* (Swedish: K670N, M671L; Florida: I716V; London: V717I) and two mutations in *PSEN1* (M146L and L286V), were used in this study [51]. Four-month-old female FAD^4T^ transgenic mice carrying two mutations in *APP* (Swedish: K670N, M671L; Indiana: V717F) and two mutations in *PSEN1* (M146L and L286V) were also used [46, 52]. Five-month-old C57BL6 female mice were used as the control. To investigate the effects of GNG5 on amyloid pathology and behaviors in vivo, 10 each 5×FAD mice were injected with 23 mg/kg EV^RVG^ (control), 23 mg/kg GNG5@EV^RVG^, and 6 μg of siGNG5@EV^RVG^. Similarly, 10 each FAD^4T^ mice were injected with 23 mg/kg EV^RVG^ (control), 23 mg/kg GNG5@EV^RVG^, and 6 μg siGNG5@EV^RVG^. EVs interventions were administered twice a week for two months and were all completed through tail vein injection.

All mice were housed in a 12-h/12-h light–dark cycle (light on from 10 a.m. to 10 p.m.) with *ad libitum* access to food and water. The live mice used in this study were approved by the Animal Experimental Welfare and Ethics Committee of the Chinese Academy of Medical Sciences. All mouse procedures were performed in accordance with the Chinese Academy of Medical Sciences Guide for the Care and Use of Laboratory Animals.

### Human brain tissue

All postmortem human brain tissues were obtained from the National Human Brain Bank for Development and Function, Chinese Academy of Medical Sciences and Peking Union Medical College, Beijing, China. Individuals were eligible for inclusion based on the following criteria: (1) a definitive pathological diagnosis of Alzheimer’s disease (AD) or control (NC); (2) absence of other CNS disorders except AD. Donor information for each experiment using human brain tissues is summarized in Table S4. Basic and detailed information for all the donors is provided in Table S1. All human tissue procedures were in accordance with the Standardized Operational Protocol for the National Human Brain Bank for Development and Function [53].

### Culture of primary dissociated neurons

Primary hippocampal neurons were isolated from postnatal day 1 rats. In brief, brains were harvested and placed in ice-cold Hank’s Balanced Salt Solution (Gibco), the meninges and cerebral cortices were removed, and the hippocampi were dissected. The hippocampi were digested with 0.25% trypsin (HyClone) and inactivated with fetal bovine serum (FBS). Hippocampal neurons obtained after centrifugation were seeded on poly-L-lysine (Sigma-Aldrich, P4707)-coated well plates. Cells were cultured in Neurobasal Medium (Thermo Fisher Scientific) supplemented with 1× B27 Supplement (50×; Thermo Fisher Scientific), 0.5 mM glutamine (Thermo Fisher Scientific), and 0.5% penicillin/streptomycin (Thermo Fisher Scientific) at 37 °C under a humidified atmosphere containing 5% CO_2_. One-half of the culture medium was changed every 2–3 days. After 2–3 weeks of culturing in vitro, hippocampal neurons were used for experiments including infection with lentivirus or EVs, IF staining, western blot.

### Cell lines

The cell line SH-SY5Y was cultured in Roswell Park Memorial Institute-1640 medium with 15% heat-inactivated FBS. The 293T cells were grown in Dulbecco’s modified Eagle’s medium supplemented with 10% FBS. Neuro-2a cells were cultivated in Minimum Essential Medium with Earle’s Balanced Salt Solution medium with 10% FBS. All media were purchased from HyClone. The cells were transfected with siRNA, either non-targeting negative control (siNC) or targeting GNG5 (siGNG5). Transfection was performed using Lipofectamine™ RNAiMAX (Invitrogen Technology), following the manufacturer’s instructions. SH-SY5Y and 293T cell line stably overexpressing APP was constructed using lentiviral infection. The concentrated lentiviral stocks were quantified using the Lenti-X TM qRT-PCR titration kit (Takara), lentivirus titer: 1 ± 0.3 × 10^8^ TU/mL, and transduced 1 × 10^7^ particles into SH-SY5Y and 5 × 10^5^ particles into 293T cells. Puromycin (Gibco, 3 μg/mL) was added to the cell culture media for 48 h as a selection marker to obtain successfully transfected cells. The procedure for acquiring SH-SY5Y and 293T cells transiently overexpressing GNG5 was the same as above method but without puromycin selection. The lentiviral infection method was used to generate an RVG-Lamp2b and GNG5 double-overexpression 293T cell line. First, RVG-Lamp2b was transfected into cells, followed by hygromycin (50 μg/mL) selection to obtain 293T-RVG^OE^ cells. Subsequently, GNG5 was transfected into 293T-RVG^OE^ cells, and the cells were selected using puromycin (3 μg/mL) to obtain 293T-RVG^OE^-GNG5^OE^ cells. All cell lines were maintained at 37 °C under 5% CO_2_.

## Method details

### Hippocampal dissection

Frozen hippocampal tissue was sectioned into 1-mm sections, alternating with 30-µm sections. The 30-µm sections were fixed for subsequent Nissl staining to determine the orientation of hippocampal subregions CA1, CA2, CA3, CA4, and EC for dissection. Since we could not accurately differentiate CA4 from DG, we combined both the tissues to obtain CA4/DG samples, which contained the pyramidal, polymorphic, and granular layers enclosed within the dentate gyrus.

### TMT labeling and MS data analysis

The hippocampal tissues were homogenized in ice-cold lysis buffer [8 M urea in phosphate-buffered saline (PBS)] with proteinase inhibitors. Soluble proteins were obtained by centrifugation at 13,400 × *g* for 15 min at 4 °C. Eight donors were divided into the NC and AD groups (four cases each). The CA1, CA2, CA3, CA4, and EC subregions were included for each donor. An equal mass of protein from the four donors in each group was pooled for digestion and MS analysis.

Proteins were treated with 10 mM dithiothreitol (DTT; Sigma-Aldrich) for 30 min at room temperature. After incubation with 25 mM iodoacetamide (IAA; Sigma-Aldrich) for 30 min at room temperature in the dark, protein digestion was completed with trypsin/Lys-C mix (Thermo Fisher Scientific) overnight at 37 °C. Subsequently, the peptides were acidified, desalted, and dried, and finally dissolved in 200 mM triethylammonium bicarbonate buffer (Sigma-Aldrich) for TMT labeling. The prepared TMT reagents were added to peptide solutions and incubated for 1 h. Two sets of TMT labeling and two technical repeats of CA1 were performed: SET 1 (6-plex): CA1_NC (TMT-126), CA2_NC (TMT-127), CA3_NC (TMT-128), CA1_AD (TMT-129), CA2_AD (TMT-130), and CA3_AD (TMT-131); SET 2 (6-plex): CA1_NC (TMT-126), CA4_NC (TMT-127), EC_NC (TMT-128), CA1_AD (TMT-129), CA4_AD (TMT-130), and EC_AD (TMT-131). The reactions were terminated by adding 5% hydroxylamine for 15 min. The labeled peptides in each set were mixed, desalted, and dried for high-performance liquid chromatography (HPLC) fractionation. The procedures for HPLC and liquid chromatography (LC)-MS/MS were as described previously [54].

The MS data were obtained and searched against the reviewed human protein FASTA database downloaded from UniProt using Proteome Discoverer 2.4 (Thermo Fisher Scientific). The search was performed using the SEQUEST-HT algorithm. A maximum of two missed trypsin/Lys-C cleavages was permitted. The mass tolerance for fragment and precursor ions was set to 20 ppm and 0.02 Da, respectively. Protein identification was considered valid when at least one peptide was detected with a false discovery rate of <1%. Relative protein intensities were quantified in accordance with the reporter ion intensities of the corresponding peptide.

The proteomic data have been uploaded to the ProteomeXchange Consortium (http://proteomecentral.proteomexchange.org) by our previous research team, and the identifier is PXD027380.

### Transcriptome sequencing and analysis

The transcriptome sequencing procedures were as described previously [55]. In brief, total RNA was extracted from human hippocampal tissues using an RNeasy Mini Kit (Qiagen). High-throughput RNA-Seq was performed using a VAHTS Total RNA-Seq Library PrepKit for Illumina (Vazyme). The reads were mapped to the human genome (assembly GRCh38) using Bowtie2 version 2.1.0, and the gene expression levels were evaluated using RSEM v1.2.15.

### Bioinformatic analysis

Venn diagrams and UpSet plots were constructed using the online Venn diagram software (http://bioinformatics.psb.ugent.be/webtools/Venn/). Heatmaps were constructed using the Hierarchical Clustering Explorer (HCE 3.5), to assess variations in protein abundance. Scatter diagrams were generated using GraphPad Prism 8 software. The cut-off value of DEPs was calculated using JMP Pro 13. Protein–protein interaction (PPI) analyses for DEPs were performed using the STRING (Search Tool for the Retrieval of Interacting Genes/Proteins) database (https://string-db.org/), and PPI networks were visualized in Cytoscape 3.6.1 software.

### IHC

Paraffin-embedded human brain tissue sections were subjected to roasting (60 °C, 20 min), deparaffinized in xylene, and dehydrated in a concentration gradient of ethanol solutions. Antigen retrieval was performed in heated citrate buffer solution for 10 min. After blocking with endogenous peroxidase blocker, the sections were incubated with anti-GNG5 primary antibody (1:200, Abcam) for 16 h at 4 °C. Further, the sections were treated with reaction enhancer reagent and goat anti-rabbit immunoglobulin (Ig) G polymer (ZSGB-BIO). Finally, the signal was detected using diaminobenzidine. The slides were rinsed with PBS three times before incubation with each reagent. After dehydration, transparency, and mounting, images of the sections were acquired. All images were quantified using Image-Pro Plus 6 software, to determine the average optical density of positively stained cells based on at least three visual fields per section.

### Dot-blotting

Human hippocampal CA1 or EC tissue homogenates were loaded on a nitrocellulose membrane. The membranes were incubated for 1 h at room temperature to ensure the blots dried before subsequent processing. Stain the membrane with Ponceau for 15 min and record the staining results, then wash off the Ponceau with 0.1 M PBS. After blocking with 5% milk for 1 h and washing three times with Tris-buffered saline with Tween 20 (TBST), the samples were incubated with anti-GNG5 (Bioworld) or anti-β-actin (GeneTex) antibodies for 1 h, followed by incubation with secondary antibody at room temperature. The blot signals were detected using ECL chemiluminescence (Millipore).

### Western blot (WB)

All cells were lysed in ice-cold radioimmunoprecipitation assay (RIPA) buffer (Beyotime) for 30 min, followed by 6 min of sonication and 20 min of centrifugation at 16,000 × *g*. After 10 min of boiling at 100 °C, proteins >10 kDa were electrophoresed on 10 or 15% Tris–glycine SDS-PAGE and transferred to 0.45-μm polyvinylidene fluoride (PVDF) membranes (Millipore). Small proteins ( < 10 kDa) were heated for 10 min at 70 °C, separated using 4–12% Bis-Tris NuPAGE (Invitrogen), and transferred on 0.22-μm PVDF membranes. The membranes were blocked with 5% non-fat milk, incubated with primary antibodies overnight at 4 °C, followed by incubation with secondary antibodies for 1 h at room temperature. After final washing with 1× TBST three times, the signal was detected using the ECL WB substrate.

Human and mouse brain tissue samples were sectioned and lysed in ice-cold RIPA buffer (Solarbio). The subsequent procedures for WB were the same as those for cell samples.

All experiments were independently repeated three times. Full and uncropped western blots are shown in Supplemental Material.

### ELISA

Aβ42 and Aβ40 concentrations were determined using commercially available Aβ ELISA kits (R & D, DAB140B for Aβ40, DAB142 for Aβ42; CUSABIO, CSB-E08299h for Aβ40, CSB-E10684h for Aβ42), according to the manufacturer’s instructions. The procedures for obtaining the lysates were the same as those described for WB. The concentrations of GNG5 in human serum and serum EVs were determined using a commercial assay kit (Nova LifeTech, ELI-47374h for human GNG5). Serum EVs were extracted using a Total Exosome Isolation (from serum) Kit (Invitrogen).

### Quantitative real-time PCR (qRT-PCR)

Total RNA was extracted from cells using the TRIzol reagent (Invitrogen). Reverse transcription and cDNA amplification reactions were performed using the One Step TB Green® PrimeScript™ PLUS RT-PCR Kit (Takara). *GAPDH* was used as the internal control. The primer sequences for the genes detected in this study are listed in the Reagent and Resource Table. The relative changes in all detected genes compared with *GAPDH* mRNA were calculated using the 2^−ΔΔCt^ method.

### Protein purification

For the production of recombinant GNG5, γ-secretase substrate C99, γ-secretase catalytic subunit PS1, and PS1 truncation (251–390), transformed *Escherichia coli* BL21 (DE3) cells were grown at 37 °C to a density of OD_600 nm_ ~ 0.5, induced with 0.2 mM isopropyl-β-D-thiogalactopyranoside, and incubated for 16 h at 22 °C. The cells were then collected, resuspended in buffer containing 200 mM Tris, 500 mM NaCl, 5 mM imidazole, and 5% glycerol, and disrupted using sonication. Following centrifugation at 27,000 × *g* for 10 min, the supernatant was subjected to ultracentrifugation at 150,000 × *g* for 1 h. The membrane fractions were resuspended in the same buffer as described above supplemented with 1.5% *n*-dodecyl-β-D-maltopyranoside (Solarbio) for 1 h at 4 °C. The suspension was centrifuged again at 150,000 × *g* for 30 min and the supernatant was loaded onto a HisTrap HP column (GE Life Science). After washing with 15 column volumes of buffer, the target proteins were eluted using a buffer containing 200 mM Tris, 500 mM NaCl, 600 mM imidazole, and 5% glycerol. The proteins were concentrated and desalted using PD-10 columns (GE Healthcare) into buffer containing 25 mM HEPES, pH 7.4, 150 mM NaCl, and 0.5% CHAPSO (Sigma-Aldrich). WB was used to visualize the purified proteins.

### Extraction of γ-secretase

Cultured cells were lysed in ice-cold buffer containing 25 mM HEPES, pH 7.4, 150 mM NaCl, and 1× cocktail (protease inhibitor) for 30 min and sonicated. The samples were centrifuged at low speed to remove intact cells, nuclei, and cell debris. The supernatant was subjected to ultracentrifugation at 150,000 × *g* for 1 h. The membrane protein fraction was then resuspended in the same buffer as described above and quantified using bicinchoninic acid. The membrane protein was diluted with the same buffer supplemented with 1% CHAPSO to a final concentration of 2.5 mg/mL and centrifuged again at 150,000 × g for 1 h. The supernatant was defined as γ-secretase. All operations were performed at low temperature.

### γ-Secretase cleavage activity assays

γ-Secretase derived from 293T-WT cells (0.2 μg/μL) was incubated with 50 nM substrate C99 in a reaction buffer containing 25 mM HEPES, pH 7.4, 150 mM NaCl, and 0.2% CHAPSO. Purified GNG5, PS1, or PS1 truncation were also added to the reaction as required. Each cleavage reaction was performed at 37 °C for 12 h. The cleaved product of the substrate (AICD) was detected using a monoclonal antibody against C1/6.1 (to detect *C*-terminal 20 amino acids of AICD, BioLegend). Aβ42 and Aβ40 production was confirmed using ELISA.

Cytoplasmic protein of Neuro-2a-APP^OE^ cells was extracted using a commercially available NE-PER^TM^ Nuclear and Cytoplasmic Extraction Reagents Kit (Thermo Fisher Scientific), per the manufacturer’s instructions. Cytoplasmic protein (1 μg/μL) was incubated with 0.2 μg/μL γ-secretase derived from 293T-NC^OE^ or 293T-GNG5^OE^ cells. The reaction conditions and Aβ detection were the same as described above.

### Extracellular vesicles (EVs) loading

Procedures for loading siRNA into EVs were as described previously [56, 57]. EVs at a total protein concentration of 6 µg (measured by NanoDrop) and 6 µg of siRNA (for in vivo injections) were mixed in 400 µL of electroporation buffer (120 mM potassium chloride, 0.15 mM calcium chloride, 10 mM potassium phosphate, 25 mM HEPES, 2 mM EGTA and 5 mM magnesium chloride, pH 7.6) and electroporated using Lonza Amaxa 4D-Nucleofector. For in vivo injection, electroporation was performed in 400 µL and pooled for subsequent ultracentrifugation before resuspension in PBS.

### Morris water maze (MWM) test

The test was performed in a circular tank filled with opaque water, and the water maze was divided into four quadrants. During the acquisition phase, all test mice were trained twice daily for five consecutive days to locate the hidden platform in the second quadrant. During this time, all mice were placed at one of the four random points in the maze and allowed to search for the hidden platform. If a mouse failed to locate the platform within 90 s, it was guided to the platform and allowed to rest for 30 s. In the probe test, the platform was removed, and time spent in the platform-located sector, number of crossings, distance traveled in the platform sector, or the proportion of distance in platform quadrant were measured to assess spatial memory.

### Step-down test

The one-trial test was conducted to measure inhibitory avoidance and memory, which included 5 min of training, followed by a 5 min test after 24 h. Briefly, the test was conducted in a chamber (~30 (h) × 12 (w) × 12 (d) cm), featuring a floor composed of an electrified grid of parallel copper bars. During the training, mice were subjected to a mild shock upon touching the electrified grid with their front paws, leading them to instinctively exhibit a tendency to jump onto the platform to avoid the shock. In the testing phase, the equipment was carefully cleaned to minimize potential odor interference.

### Open-field test

This task was used to assess the locomotor activity and exploratory behavior of the test mice. All mice were individually placed in a 30 (h) × 60 (w) × 60 (d) cm arena for 5 min individually. The arena was divided into central and peripheral zones. Total number of grid crossings was recorded to evaluate the movement ability of mice, and the time spent in the central zone was measured to assess anxiety of the mice. All counts were performed using a double-blind method.

### Co-immunoprecipitation (co-IP)

Membrane proteins extracted from 293T cells stably transfected with GNG5 were incubated with anti-FLAG (Sigma-Aldrich) or anti-IgG (ZSGB-BIO) antibody for 1 h at 4 °C, pulled-down with Protein A/G PLUS-Agarose (Santa Cruz), washed four times for 5 min each in co-IP buffer (20 mM HEPES, pH 7.5, 50 mM KCl, 2 mM EGTA, 0.25% CHAPSO, 1× protease inhibitor cocktail), and collected as the co-IP products. The harvested samples were analyzed.

### In-gel digestion followed by MS

The above co-IP samples were resolved into six fractions on 15% SDS-PAGE gel (Tris–glycine gel). The gel fractions were cut into pieces and processed for in-gel digestion. Briefly, gel pieces were washed with acetonitrile and ammonium bicarbonate. After destaining and shrinking, the pieces were treated with 25 mM DTT for reduction, followed by 25 mM IAA for alkylation. The in-gel protein digestion was performed with trypsin/Lys-C at 37 °C overnight, and the digested proteins were then extracted for MS analysis. Protein identification was performed using the Protein Discoverer 2.2 software.

### Plasmids and siRNAs

The following DNA constructs were used in this study: LvCP06-empty (Era Biotech), LvCP06-GNG5-FLAG (Era Biotech), and pcDNA GNSTM-3-RVG-10-LAMP2b-HA (Addgene).

The following pre-designed siRNAs were used in this study: control non-targeting siRNA, GNG5 Smart Silencers, GRK6 Smart Silencers, CXCR2 Smart Silencers, GNAI1 Smart Silencers, and GNAO1 Smart Silencers. All siRNAs were obtained from RIBOBIO.

### Transfection

Lipofectamine™ RNAiMAX (Invitrogen) was used for siRNA transfections, according to the manufacturer’s instructions. Transfection was performed in six-well plates. For silencing experiments, cells were transfected with a single round of 15 nM siRNA for 48 h and then collected for analysis.

### Lentivirus production

Lentivirus particles were produced and transduced according to the following protocols. Briefly, 293T cells growing in 10-cm dishes were transfected with a mix of 10 μg DNA (5 μg targeting DNA, 2.5 μg GAG, 1 μg REV, and 1.5 μg pVSVG). Lipofectamine 2000 (Invitrogen) was used as the DNA transfection reagent. After transfection for 48 h, cell culture medium was collected and replaced by new medium. Cell culture medium was collected again 24 h later. Virus preparations were then concentrated with PEG 8000. Lentivirus particles were obtained by centrifugation at 4000 × *g* for 20 min at 4 °C.

### IF and confocal microscopy analyses

To analyze Aβ42 and Aβ40 signals in the hippocampus and cortex of the mouse models, 12-μm left brain sections were excised using a cryostat and processed for IF staining. Tissues were washed in PBS five times to remove Optimal Cutting Temperature (O.C.T.) compound and processed for 15 min with 0.3% Triton X-100. After blocking for 1 h [PBS supplemented with 0.1% Triton X-100 and 5% bovine serum albumin (BSA)], sections were incubated with primary antibodies overnight at 4 °C. The sections were washed three times and incubated with secondary antibody for 30 min at room temperature. Imaging was performed using a STED super-resolution confocal microscope (Leica TCS SP8 STED 3X) after 4′,6-diamidino-2-phenylindole (DAPI) staining and mounting.

Hippocampal primary neurons cultured for 20 days in vitro or other cells were fixed with paraformaldehyde, then treated with 0.3% Triton X-100 for 5 min and blocked with 5% BSA for 30 min. Next, the cells were incubated with primary antibodies overnight at 4 °C, followed by fluorescent secondary antibody incubation. After nuclear staining with DAPI and mounting, image acquisition was performed with a confocal microscope. Images in Figs. 4M, 4O, and 4P were deconvolved using Huygens Essential and 3D rendered in Imaris and quantified using Fiji (ImageJ).

### IF of paraffin-embedded human brain tissue

Paraffin-embedded human brain tissue sections were subjected to roasting (60 °C, 20 min), deparaffinized in xylene, and dehydrated in a concentration gradient of ethanol solutions. After the slides were permeabilized with 0.3% Triton X-100 for 30 min, antigen retrieval was performed in heated citrate buffer solution for 10 min. Next, slices were incubated with 0.5% sodium borohydride for 10 min and goat serum for 30-min blocking. The slides were incubated with anti-GNG5 (1:50, Abcam), PS1 (1:50, Invitrogen), CXCR2 (1:50, Abcam) and Aβ42 (1:50, CST) for 16 h at 4 °C. Incubations with fluorescent secondary antibody and Sudan black were performed for 30 and 10 min, respectively, and the slice images were obtained after sealing. The slides were rinsed with PBS three times before incubation with each reagent.

### Isolation of EVs from human brain tissues

Human frontal brain tissue was used to extract EVs. The tissue samples were incubated with dispase (Roche) at 37 °C for 1 h, followed by the addition of DNase (Solarbio) and cocktail (protease inhibitor, Roche). After differential centrifugation, the supernatant was subjected to ultrafiltration. The obtained sample was loaded onto a qEVoriginal/70 nm Gen 2 column (IZON) and EVs were harvested using PBS solution, according to the manufacturer’s instructions. Ultrafiltration was performed to obtain EVs in a final volume of ~100 μL.

### Extraction of EVs from human serum

Frozen serum samples were thawed in a water bath at 25 °C and centrifuged at 2000 × *g* for 30 min at room temperature; 0.2 volumes of Exosome Isolation Reagent (Invitrogen) were added to the serum, which was incubated on ice for 30 min. The EVs pellet was collected after centrifugation at 10,000 × *g* for 10 min at room temperature. Informed consent was obtained from all the patients, the study was performed with the approval of the Ethics Committee of PUMCH.

### Neuron-derived EVs (NEDVs) isolation

The EVs from human serum pellet was resuspended in 350 µL of ultra-pure distilled water supplemented with protease/phosphatase inhibitors overnight with gentle rotation mixing at 4 °C. EVs was incubated for 30 min at RT with 2 µg of biotinylated anti-human L1CAM antibody (clone 5G3) (cat. no. 13-1719-82; Thermo Fisher Scientific) to derive NDEVs. The EV-antibody complexes were then incubated with 10 µL of Pierce™ Streptavidin Magnetic Beads (cat. no. 88816; Thermo Fisher Scientific) for 30 min at RT. After centrifugation at 600 × *g* for 10 min at 4 °C and removal of supernatant, NDEVs was eluted with 100 µL of 0.1 M glycine (stock solution at 1 M, pH = 2.7; cat. no. 24074-500; Polysciences, Inc.). Beads were sedimented by centrifugation at 4000 × *g* for 10 min at 4 °C, and supernatant containing immunoprecipitated EVs was transferred to a clean tube, where pH was immediately neutralized with 10 µL of 1 M tris hydrochloride (Tris-HCL, pH = 8; cat. no. CAS1185-53–1; Fisher Scientific). EVs was lysed in ice-cold radioimmunoprecipitation assay (RIPA) buffer (Beyotime) for 30 min, followed by 6 min of sonication and 20 min of centrifugation at 16,000 × *g*.

### Isolation of EVs from cell supernatant

The 293T-RVG^OE^ and 293T-RVG^OE^-GNG5^OE^ cells were used to produce EV^RVG^ and GNG5@EV^RVG^, respectively. The culture media were collected and centrifuged several times until no significant pellet was observed. The supernatant samples and Exosome Concentration Solution (Umibio) were mixed at a volume ratio of 4:1 and allowed to stand for 12 h, followed by centrifugation to obtain EV particles. The EVs were resuspended in PBS and centrifuged several times to collect the supernatant, which was then loaded on an Exosome Purification Filter (Umibio) and centrifuged to obtain purified EVs. Quantification of proteins was performed using a Pierce™ BCA Protein Assay Kit (Thermo Fisher Scientific). Imaging of EVs was performed using transmission electron microscopy. In nanoparticle tracking analysis, Zetaview (ParticleMetrix) was used to analyze the size distribution and concentration of EVs.

### Fluoro-Jade C staining

Neurodegeneration was assessed using Fluoro-Jade C staining of the brain slices from EV^RVG^- and GNG5@EV^RVG^-treated mice, followed by laser scanning confocal microscopy, as described previously [58]. The sections were dried at 55 °C for 1 h before staining. Slides were immersed in sodium hydroxide for 5 min, then for 2 min in 70% alcohol, and 2 min in distilled water. Subsequently, the slices were transferred to a potassium permanganate solution for 10 min and rinsed in distilled water for 2 min. After 10 min in the staining solution, the slices were washed three times (1 min each) in redistilled water. The slides were dried at 55 °C and then collected for image acquisition after mounting.

### Molecular docking

The structures of the γ-secretase–C83 complex (Protein Data Bank [PDB] code 6IYC) and the CXCL8–CXCR2–Gαi–Gβγ complex (PDB code 6LFO) were obtained from the RCSB Protein Data Bank. Prediction of protein and peptide structures in this paper was performed using AlphaFold2. Rigid-body protein–protein docking was completed using ZDOCK, which uses the Fast Fourier Transform algorithm to enable an efficient global docking search on a 3D grid, followed by scoring [59]. RosettaDock, based on the Monte Carlo algorithm, was used to search the rigid-body and side-chain conformational space of two interacting proteins and to find minimum free-energy complex structures [60]; structures with lower energies are considered to be better than those with higher energies. The three best-scoring structures in rank order by energy were selected for further analysis. The docking results were visualized using the molecular graphics system, PyMOL.

### Quantification and statistical analyses

Statistical analyses in this study were performed using SPSS software v21 and GraphPad Prism v8. Data are presented as the mean value ± SD. Two-tailed unpaired Student’s *t*-tests for two groups and one-way ANOVA with Turkey post hoc test for multiple groups were used for statistical analyses. Pearson’s and Spearman’s correlation coefficients were calculated to evaluate correlations of continuous and categorical variables, respectively. The Kruskal–Wallis test was used for non-normally distributed variables. Statistical significance was defined as *p* < 0.05.

## Supplementary information

Supplementary Figure and Table Legends

Supplementary Figure 1

Supplementary Figure 2

Supplementary Figure 3

Supplementary Figure 4

Supplementary Figure 5

Supplementary Figure 6

Supplementary Figure 7

Supplementary Figure 8

Supplementary Figure 9

Supplementary Figure 10

Supplementary Figure 11

Supplementary Figure 12

Supplementary Figure 13

Supplementary Figure 14

Supplementary Figure 15

Supplementary Figure 16

Supplementary Figure 17

Supplementary Table 1

Supplementary Table 2

Supplementary Table 3

Supplementary Table 4

Original data files

Statistical analysis

aj-checklist

## Data Availability

The datasets analysed during the current study are available in the ProteomeXchange Consortium repository, http://proteomecentral.proteomexchange.org, PXD027380.
