# Supplementary material for: GNG5 is a novel regulator of Aβ42 production in Alzheimer’s disease
Source: Cell Death Dis. 2024 Nov 11;15(11):815. doi: 10.1038/s41419-024-07218-z (PMC11554683; doi:10.1038/s41419-024-07218-z)
Supplement: Supplementary file 1 — Supplementary Figure and Table Legends [file 41419_2024_7218_MOESM1_ESM.docx]

**Supplementary Figure legends**

**Fig. S1 A schematic representation of the workflow used to study the proteomic and transcriptomic changes in the hippocampal–entorhinal region (CA1,** **CA2, CA3, CA4, and EC) from donors with cognitively normal controls (NC) and with pathological AD.** CA, cornu ammonis; EC, entorhinal cortex; TMT, tandem mas tag; HPLC, high-performance liquid chromatography; LC-MS/MS, liquid chromatography-tandem mass spectrometry.

**Fig. S2 Screening of DEPs in proteomic analysis and transcriptional alterations of *GNG12* and *GNB4* mRNA levels in the hippocampal–entorhinal regions.**

**A** Venn diagram of the confident proteins identified in two SETs of proteomic technical replicates (SET1 and SET2) of the hippocampal CA1 subregion.

**B** Correlation analysis of protein abundance ratios (AD/NC) in SET1 and SET2.

**C** Prediction of cut-off values for DEPs in CA1, CA2, CA3, CA4, and EC using JMP13.

**D** The number and proportion of DEPs in CA1, CA2, CA3, CA4, and EC.

**E, F** Counts per million (CPM) of *GNG12* and *GNB4* in transcriptomic analysis of CA1, CA2, CA3, CA4, and EC subregions from donors with neuropathological assessment N, L, I, or H. N, *n* = 13-15; L, *n* = 5-6; I, *n* = 17; H, *n* = 14. one-way ANOVA with Turkey post hoc test was used for multiple groups. **p* < 0.05, ***p* < 0.01.

**Fig. S3 Verification of GNG5 expression.**

**A** Immunohistochemical staining with anti-GNG5 antibody (Abcam, ab238835) in postmortem hippocampal–entorhinal subregions from NC (*n* = 13) and pathological AD (*n* = 13) donors. Scale bar, 50 µm.

**B** Western blot detection of recombinant GNG5 with anti-GNG5 antibody (Bioworld Technology, BS61200). The red arrow indicates trimeric GNG5.

**C** Dot blot and densitometry analyses of GNG5 levels (Bioworld Technology, BS61200) in CA1 and EC from NC and pathological AD donors. Total amount of each sample was quantified by ponceau staining and as loading control. For CA1, NC (*n* = 29, 85.9 ± 8.4 yr), AD (*n* = 26, 84.2 ± 6.4 yr). For EC, NC (*n* = 45, 88.6 ± 7.8 yr), AD (*n* = 41, 87.4 ± 6.8 yr). Data are presented as mean value ± SD. Two-tailed unpaired Student’s *t*-test was used for two groups. ****p* < 0.001.

**Fig. S4 Effect of GNG5 on APP and PS1 mRNA and protein levels.**

**A** Relative mRNA levels of *APP* detected using qRT-PCR in SH-SY5Y and 293T cells transfected with control vector or APP expression vector.

**B** Relative mRNA levels of *GNG5*, *APP*, and *PS1* detected using qRT-PCR in SH-SY5Y-APP^OE^-NC^OE^ and SH-SY5Y-APP^OE^-GNG5^OE^ cells.

**C** Relative mRNA levels of *GNG5*, *APP*, and *PS1* detected using qRT-PCR in 293T-APP^OE^-NC^OE^ and 293T-APP^OE^-GNG5^OE^ cells.

**D** Relative mRNA levels of *GNG5*, *APP*, and *PS1* detected using qRT-PCR in SH-SY5Y-APP^OE^ cells transfected with non-targeting negative control siNC or siGNG5.

**E** Relative mRNA levels of *GNG5*, *APP*, and *PS1* detected using qRT-PCR in 293T-APP^OE^ cells transfected with siNC or siGNG5.

**F, G** Knockdown of GNG5 in Neuro-2a using siNC or siGNG5. (**F**) Relative mRNA levels of *GNG5* detected using qRT-PCR. (**G**) Western blot detection of PS1, APP, and p-Tau (S396).

**H** Coomassie brilliant blue staining of total γ-secretase complex extracts was used as reference control.

**I** Western blot verification of recombinant C99, full length PS1, and PS1 Ala_251_–Ser_390_ fragment with anti-His antibody. Data are presented as the mean ± SD. *p* values were determined using unpaired two-tailed Student's *t*-test for two groups and one-way ANOVA with Turkey post hoc test for multiple groups. **p* < 0.05, ***p* < 0.01, ****p* < 0.001, ns: not significant.

**Fig. S5 Efficient delivery of GNG5@EV^RVG^ and siGNG5@EV^RVG^ into 5×FAD and FAD^4T^ mice brains.**

**A** qRT-PCR and Western blot detection of *Lamp2b* mRNA and protein levels in 293T and 293T-RVG^OE^ cells.

**B** Western blot and qRT-PCR detection of GNG5, and EV markers (LAMP2b, CD63, ALIX, and TSG101) in 293T-RVG^OE^ and 293T-RVG^OE^-GNG5^OE^ cells.

**C** Relative mRNA levels of *GNG5* detected using qRT-PCR in constructed EV^RVG^ and GNG5@EV^RVG^.

**D** Particle diameter distribution and concentration of EV^RVG^, GNG5@EV^RVG^, and siGNG5@EV^RVG^, analyzed using ZetaView.

**E** *In vivo* tracking of PKH-26-labeled siGNG5@EV^RVG^ and GNG5@EV^RVG^.

**F** Verification of GNG5 overexpression and knockdown in the hippocampus of 5×FAD and FAD^4T^ mice by ELISA. *n* = 10 mice per group.

**G–H** Representative fluorescence micrographs and quantification of amyloid plaques (anti-6E10) and Aβ42^+^ plaques (anti-Aβ42) in the hippocampus of indicated mice. *n* = 10 mice per group. Data are presented as the mean ± SD. *p* values were determined using one-way ANOVA with Turkey post hoc test for multiple groups. **p* < 0.05, ***p* < 0.01, ****p* < 0.001, ns: not significant.

**Fig. S6 Immunoprecipitation/mass spectrometry (IP/MS) detection of GNG5-interacting membrane proteins.**

**A** Schematic representation of the IP/MS approach to identify GNG5-interacting membrane proteins, using anti-Flag tag fused at GNG5 *C*-termini followed by SDS-PAGE separation, in-gel digestion, and peptides identification using Orbitrap Exploris 480 (Thermo).

**B** Western blot verification of GNG5 in FLAG-IP products from 293T-GNG5^OE^ membrane proteins. The red arrow indicates the bands for endogenous GNG5.

**C** SDS-PAGE separation of immunoprecipitated GNG5-interacting proteins into 6 fractions.

**D** Classification of representative proteins co-interacted with GNG5 identified via IP-MS detection.

**E** Gene Ontology (GO) enrichment and pathway analyses of GNG5-interacting proteins using the FunRich software.

**Fig. S7 Double immunofluorescence staining of GNG5 (red) and PS1 (green) in postmortem brains.**

**A** Representative image from eight brain regions related to the A score (hippocampus, precentral gyrus, visual cortex, inferior parietal lobule, midbrain, superior temporal gyrus, cerebellum, and basal nucleus) of donor AD12. Scale bar, 20 µm.

**B** Representative images in hippocampus from 6 NC and 2 pathological AD donors. Scale bar, 20 µm.

**Fig. S8 Chemical structures of the γ-secretase inhibitors E2012, Semagacestat, and Avagacestat.**

**Fig. S9** **Representative confocal images of RAB5 co-localization with GNG5 and PS1 in 293T cells.**

**Fig. S10 GNG5 regulates protein expression and activation levels of Rab5.**

**A** Relative mRNA levels of *Rab5* in 293T-NC^OE^ and 293T-GNG5^OE^ cells.

**B** Relative mRNA levels of *GNG5* in rat primary hippocampal neurons transfected with control vector or GNG5 expression vector.

**C** IF staining detection of RAB5 protein levels in WT and GNG5-overexpressing primary hippocampal neurons, including a GNG5 lentivirus transfection group, incubation with human brain-derived EV group (NC8_EV, which have been confirmed to contain a high GNG5 protein levels), and treatment with the GNG5@EV^RVG^ group. Statistical analyses for RAB5 intensity, puncta size, and area fraction are shown.

**D** Confocal microscopic staining of Rab5 in SH-SY5Y-NC^OE^ and SH-SY5Y-GNG5^OE^ cells. Statistical analyses for RAB5 intensity, puncta size, and area fraction are shown.

**E** Extraction of Golgi apparatus from 293T-NC^OE^ and 293T-GNG5^OE^ cells using commercial kits, followed by quantification for Aβ42 and Aβ40 between the two groups using ELISA.

**F, G** qRT-PCR quantification (**F**) and western blot detection (**G**) of Rab5 mRNA and protein levels in 293T cells transfected with non-targeting negative control siRNA or GNG5-targeting siRNA.

**H** Confocal microscopic staining of Rab5 in 293T-GNG5^OE^ cells transfected with non-targeting negative control siRNA or GNG5-targeting siRNA. Data are presented as the mean ± SD. *p* values were determined using unpaired two-tailed Student's *t*-test for two group comparison and one-way ANOVA with Turkey post hoc test for multiple group comparison. * *p* < 0.05, ** *p* < 0.01.

**Fig. S11 Expression changes of GNG5 in Aβ-stimulated cell models.**

**A** Relative mRNA levels of *GNG5* in oAβ42- or oAβ40-stimulated (0, 0.01, 0.1, 10, 100, or 1000 nM) primary hippocampal neuron model.

**B, C** Relative mRNA levels (**B**) and western blot analysis (**C**) of GNG5 in oAβ42- or oAβ40-stimulated (0, 0.01, 0.1, 10, 100, and 1000 nM) SH-SY5Y cell model.

**D, E** Relative mRNA levels (**E**) and western blot analysis (**D**) of GNG5 in oAβ42- or oAβ40-stimulated (0, 0.01, 0.1, 10, 100, or 1000 nM) 293T cell model.

**F** Relative mRNA levels and western blot analysis of GNG5 in monomeric Aβ42 (mAβ42, 5 or 10 μM)-stimulated 293T. Data are presented as the mean ± SD. *p* values were determined using unpaired two-tailed Student's *t*-test for two groups and one-way ANOVA with Turkey post hoc test for multiple groups. ns: not significant.

**Fig. S12 GNG5 regulates the protein levels and localization of Rab5 in Aβ-stimulated cell models.**

**A, B** Representative fluorescence micrographs and quantification of Rab5 in oAβ42- or oAβ40-stimulated 293T and SH-SY5Y cell models. Scale bar, 10 μm.

**C, D** Representative confocal images and quantification of Rab5 in oAβ42-stimulated 293T and SH-SY5Y cell model transfected with non-targeting negative control siRNA or GNG5-targeting siRNA. Scale bar, 10 μm. Data are presented as the mean ± SD. *p* values were determined using one-way ANOVA with Turkey post hoc test for multiple groups. **p* < 0.05, ns: not significant.

**Fig. S13 CXCR2 and Aβ42 co-localize in neurons in eight brain regions associated with the A score of AD pathology.**

**A** Confocal fluorescence images of CXCR2 (red) and Aβ42 (green) co-localization in 8 brain regions (precentral gyrus, hippocampus, superior temporal gyrus, inferior parietal lobule, visual cortex, basal nucleus, cerebellum, and midbrain) related to the A score from AD12. Apparent co-localization between CXCR2 and Aβ42 in yellow. Scale bar, 20 μm.

**B** Representative confocal images of CXCR2 (green) and Aβ42 (red) co-localization in hippocampal neurons from 6 NC and 2 AD samples. Apparent co-localization between CXCR2 and Aβ42 in yellow. Scale bar, 20 μm. anti-CXCR2 (Proteintech, 20634-1-AP), anti-Aβ42 (Cell Signaling Technology, 14974).

**Fig. S14 Molecular docking of predicted CXCR2 with Aβ42 and GNG5 molecules.**

**A** Sequence alignment between GNG5 and GNG2.

**B** Structural alignment of predicted GNG5 (Gγ5, G protein γ subunit 5) using AlphaFold2 with the solved structure of active CXCR2–Gαi–Gβ–Gγ2.

**C** Molecular docking of CXCR2 with one or three Aβ42 molecules using AlphaFold2.

**Fig. S15 Effects of CXCR2-related regulatory proteins on GNG5 protein and mRNA levels.**

**A, B** qRT-PCR detection of *GNAI1* and *GNG5* mRNA in 293T-GNG5^OE^ cells with GNAI1 knockdown.

**C** qRT-PCR detection of *GNAO1* in 293T-GNG5^OE^ cells with GNAO1 knockdown.

**D** Western blot detection of GNG5 in 293T-GNG5^OE^ cells with GNAO1 knockdown.

**E, R** qRT-PCR detection of *CXCR2* and *GNG5* mRNA in 293T-GNG5^OE^ cells with CXCR2 knockdown.

**G** Representative confocal images of Aβ42 in 293T-APP^OE^, 293T-APP^OE^-GNG5^OE^, and 293T-APP^OE^-GNG5^OE^ cells with CXCR2 knockdown.

**H** Western blot of CXCR2 in EVs derived from the cerebral cortex of 9 AD and 10 NC brain donors. NC: 82.3 ± 5.3 yr; AD: 82.4 ± 5.3 yr. Detailed information about the donors is presented in Table S1. Data are presented as the mean ± SD. *p* values were determined using unpaired two-tailed Student's *t*-test for two groups and one-way ANOVA with Turkey post hoc test for multiple groups. ***p* < 0.01, ns: not significant.

**Fig. S16 Analysis of GNG5 expression with AD pathological and clinical indicators.**

**A** Relative expression of GNG5 with A scores in the CA2, CA3, CA4, and EC subregions. Median values are represented by lines. r_s_: Spearman correlation coefficient. A0 (*n* = 9, 81.2 ± 9.6 yr), A1 (*n* = 7, 91.3 ± 5.1 yr), A2 (*n* = 2, 88.5 ± 3.5 yr), A3 (*n* = 8, 88.8 ± 5.5 yr).

**B** Representative IHC images of GNG5 and Aβ plaques with anti-GNG5 and anti-6E10 (to detect Aβ) antibodies in the CA1 subregion. NC (*n* = 6, 81.7 ± 10.6 yr), AD (*n* = 5, 90.3 ± 4.7 yr). Black arrows point to Aβ plaques. Scale bar, 20 μm.

**C** Pearson correlation analyses between trimeric GNG5 levels (**Fig. 8C**) and Aβ40 levels (**Fig. 8D**).

**D** Characteristics of study participants and serum GNG5 levels. Data are presented as the mean ± SD. *p* values were determined using one-way ANOVA with Turkey post hoc test for multiple groups.

**E** Western blot detection and densitometry analysis of GNG5 levels in the NDEVs. CN (*n* = 5, 86.1 ± 6.3 yr), AD (*n* = 5, 88.4 ± 7.4 yr).

**Fig. S17. GNG5 is a potential upstream regulator of Aβ42.**

**A** Comparison of the correlation analysis between GNG5 and age in EVs derived from control and AD brain tissues, related to Fig. 8F. Kruskal–Wallis test was used for non-normally distributed variables, IntDen, Integrated Density.

**B** ELISA measurement of GNG5 in the brain tissues of WT and APP/PS1 mice (*n* = 10 mice group, 2-month-old). Unpaired two-tailed Student's t-test.

**C** IF micrographs of amyloid plaques with 6E10 antibody in APP/PS1 mice brain (*n* = 10 mice per group, 2-month-old). ns: not significant.

**Supplementary Table legends**

**Table S1.** Detailed information about the donors used in this study.

**Table S2.** Confident proteins identified in proteomic analysis of hippocampal-entorhinal subregions.

**Table S3.** CPM of *GNG5* in transcriptomic analysis of hippocampal-entorhinal subregions.

**Table S4.** Key Resource Table.
