# Supplementary material for: GNG5 is a novel regulator of Aβ42 production in Alzheimer’s disease
Source: Cell Death Dis. 2024 Nov 11;15(11):815. doi: 10.1038/s41419-024-07218-z (PMC11554683; doi:10.1038/s41419-024-07218-z)
Supplement: Supplementary file 23 — Original data files [file 41419_2024_7218_MOESM23_ESM.pdf]

Figure 2A

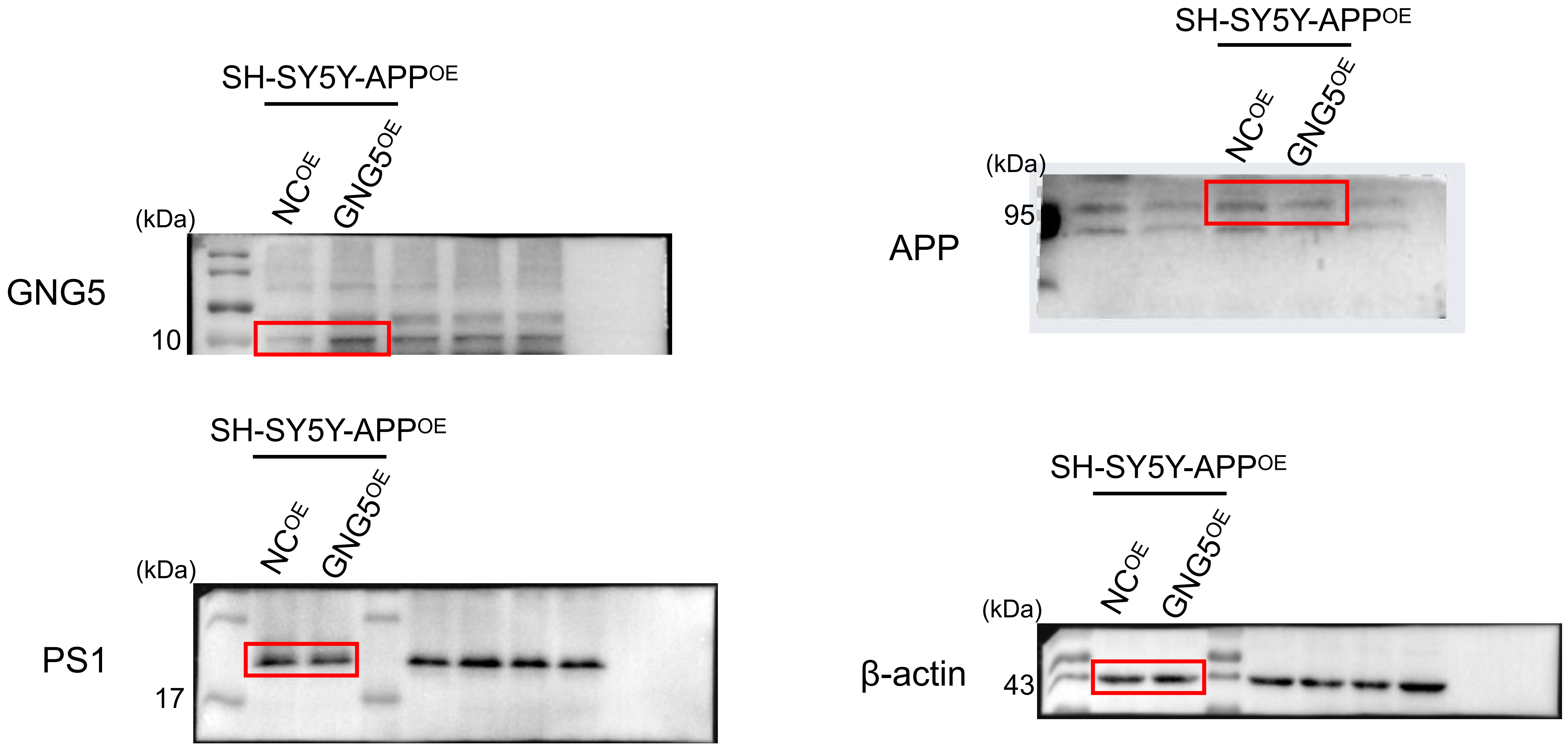

SH-SY5Y-APPOE
NCOE
GNG5OE
(kDa)
GNG5
10
SH-SY5Y-APPOE
NCOE
GNG5OE
(kDa)
APP
95
SH-SY5Y-APPOE
NCOE
GNG5OE
(kDa)
PS1
17
SH-SY5Y-APPOE
NCOE
GNG5OE
(kDa)
β-actin
43

Figure 2C

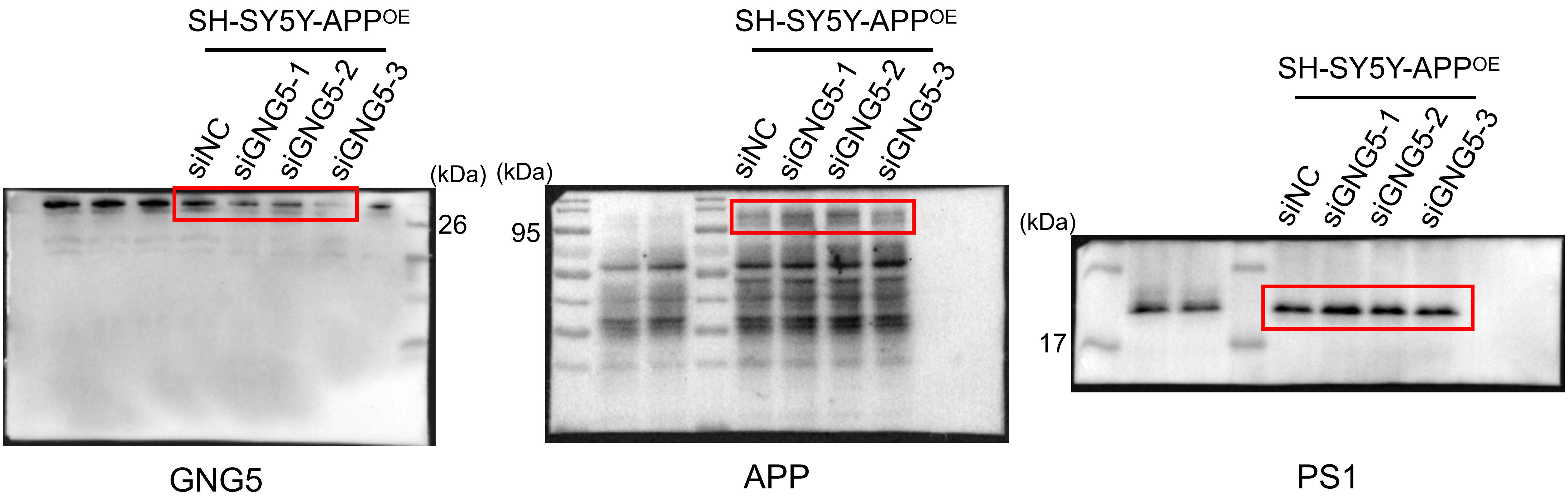
SH-SY5Y-APPOE
siNC
siGNG5-1
siGNG5-2
siGNG5-3
(kDa)
26
GNG5
SH-SY5Y-APPOE
siNC
siGNG5-1
siGNG5-2
siGNG5-3
(kDa)
95
APP
SH-SY5Y-APPOE
siNC
siGNG5-1
siGNG5-2
siGNG5-3
(kDa)
17
PS1

Figure 2C

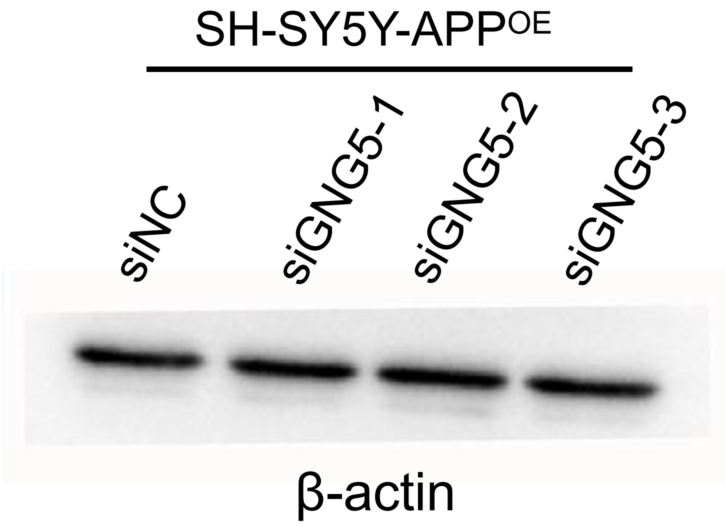
SH-SY5Y-APPOE
siNC
siGNG5-1
siGNG5-2
siGNG5-3
β-actin

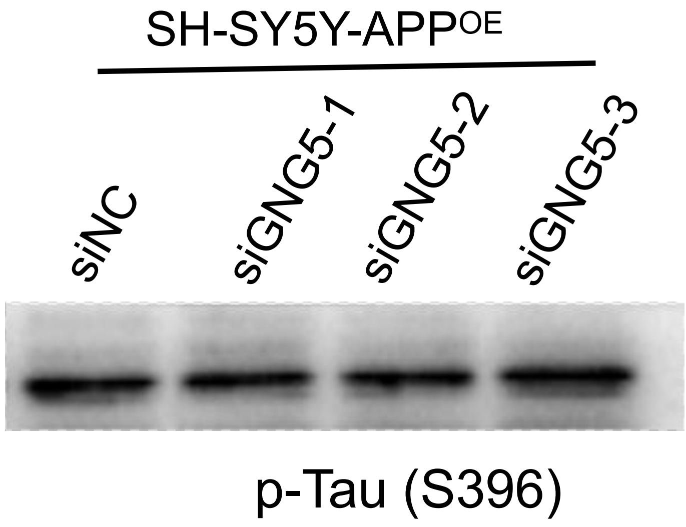
SH-SY5Y-APPOE
siNC
siGNG5-1
siGNG5-2
siGNG5-3
p-Tau (S396)

Figure 2E

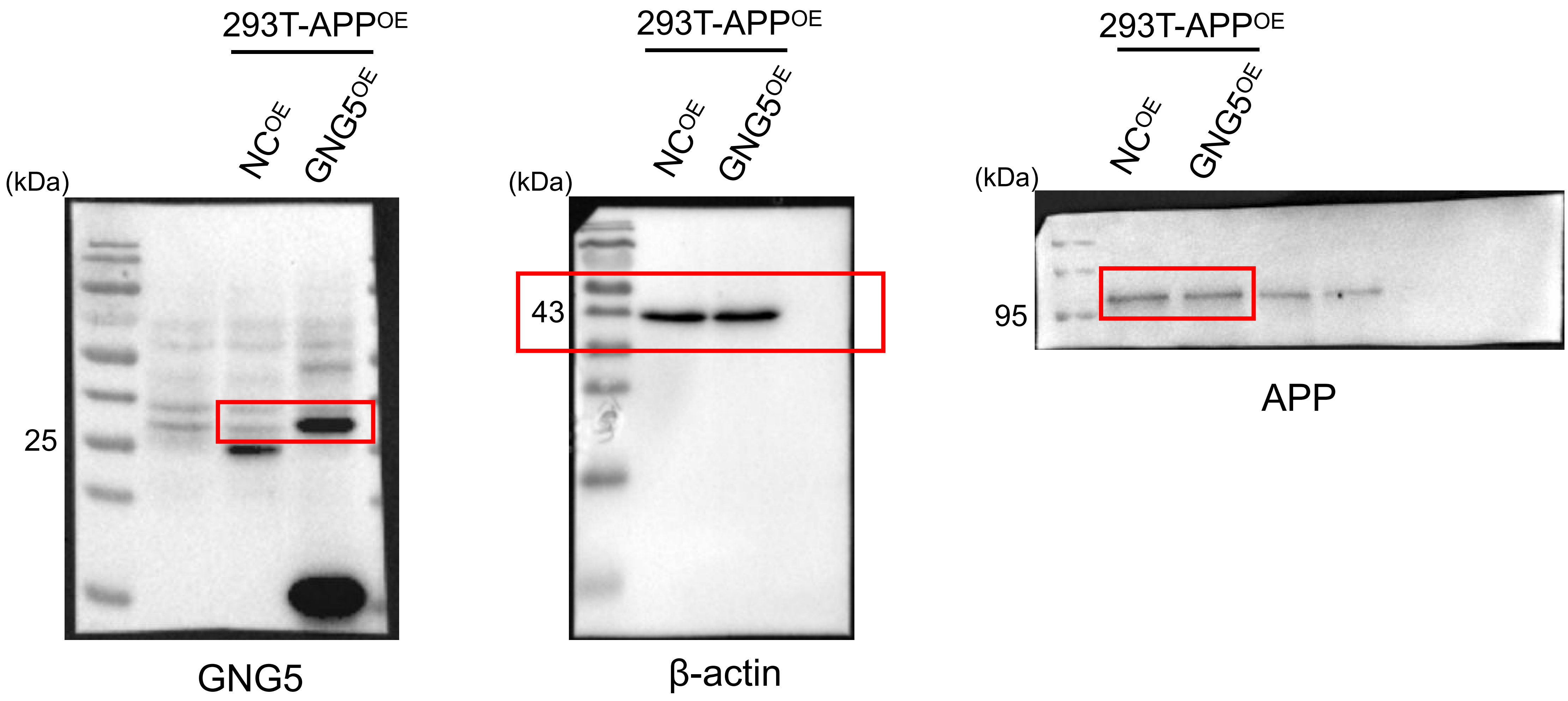
293T-APPOE
NCOE
GNG5OE
(kDa)
25
GNG5
293T-APPOE
NCOE
GNG5OE
(kDa)
43
β-actin
293T-APPOE
NCOE
GNG5OE
(kDa)
95
APP

Figure 2E

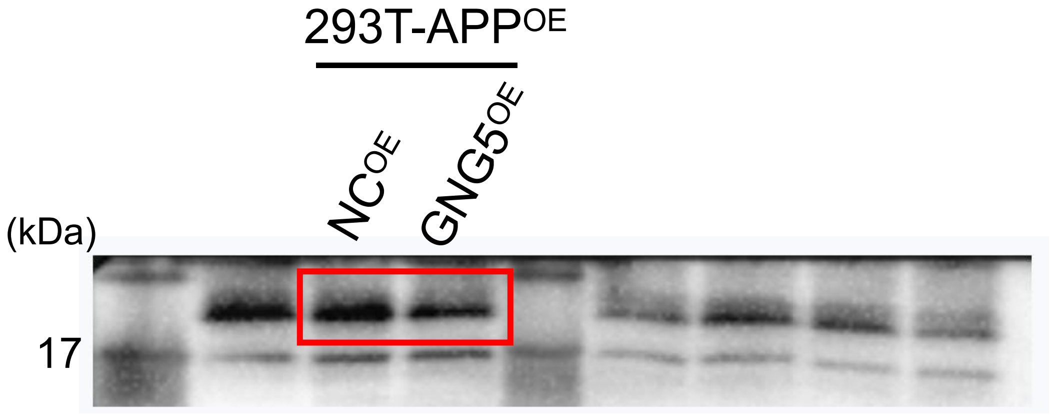

PS1

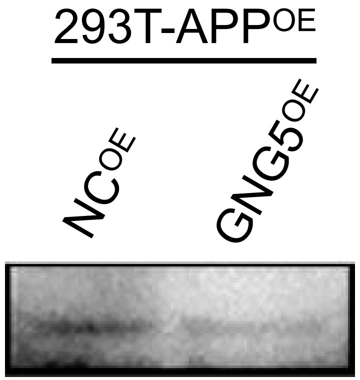

p-Tau (AT8)

Figure 2G

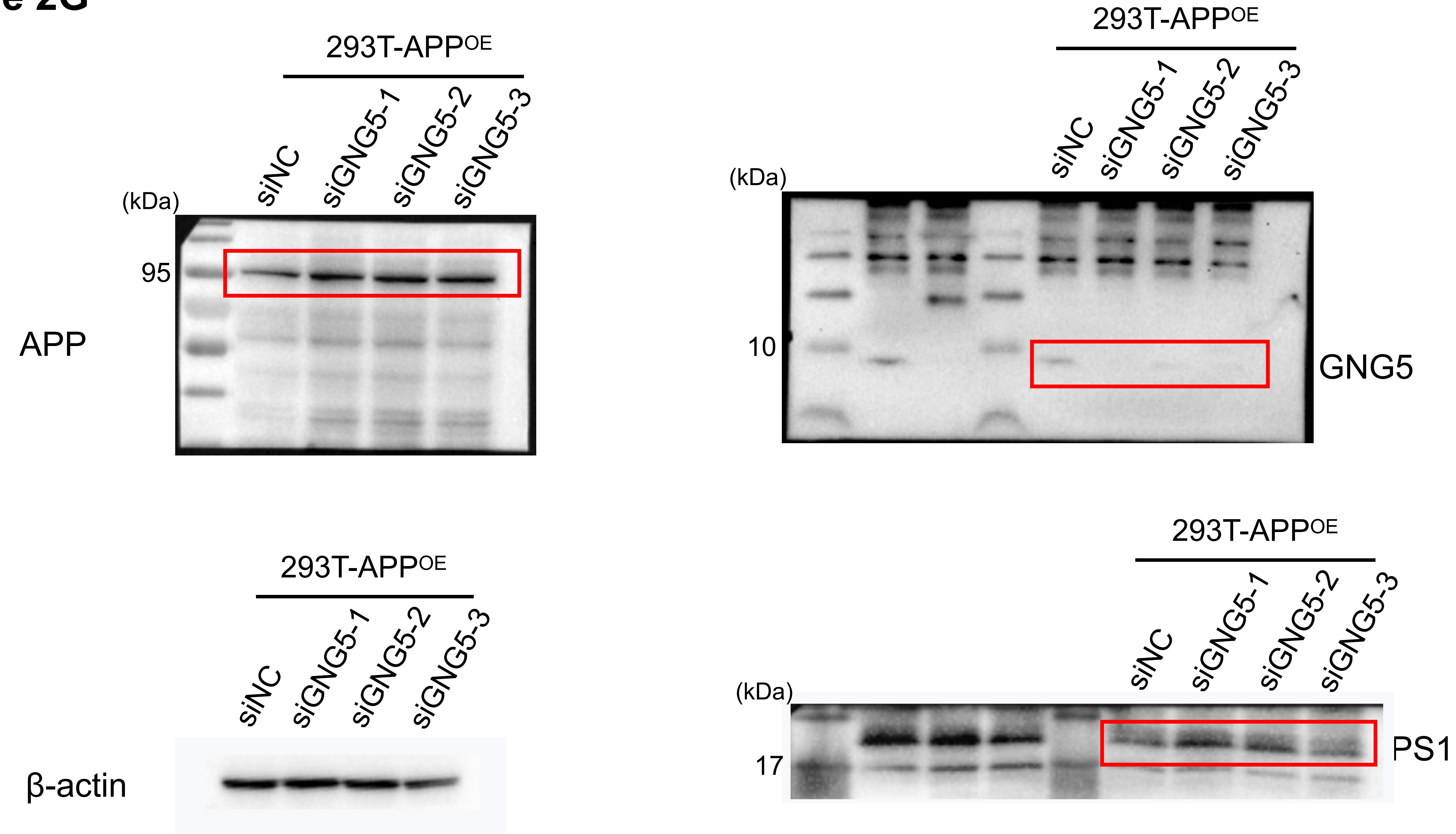

293T-APP^{OE}
siNC
siGNG5-1
siGNG5-2
siGNG5-3
(kDa)
95
APP
293T-APP^{OE}
siNC
siGNG5-1
siGNG5-2
siGNG5-3
(kDa)
10
GNG5
293T-APP^{OE}
siNC
siGNG5-1
siGNG5-2
siGNG5-3
β-actin
293T-APP^{OE}
siNC
siGNG5-1
siGNG5-2
siGNG5-3
(kDa)
17
PS1

Figure 2I

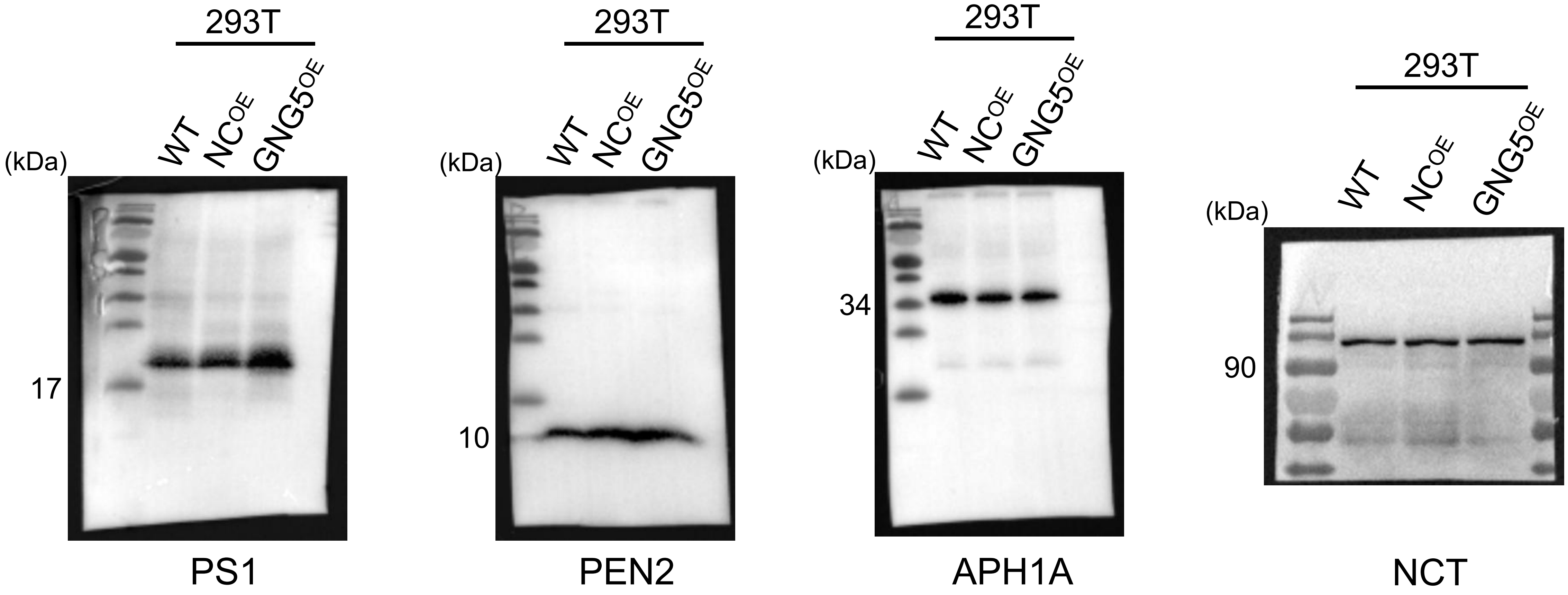
293T
WT
NCOE
GNG5OE
(kDa)
17
PS1
293T
WT
NCOE
GNG5OE
(kDa)
10
PEN2
293T
WT
NCOE
GNG5OE
(kDa)
34
APH1A
293T
WT
NCOE
GNG5OE
(kDa)
90
NCT

Figure 2I

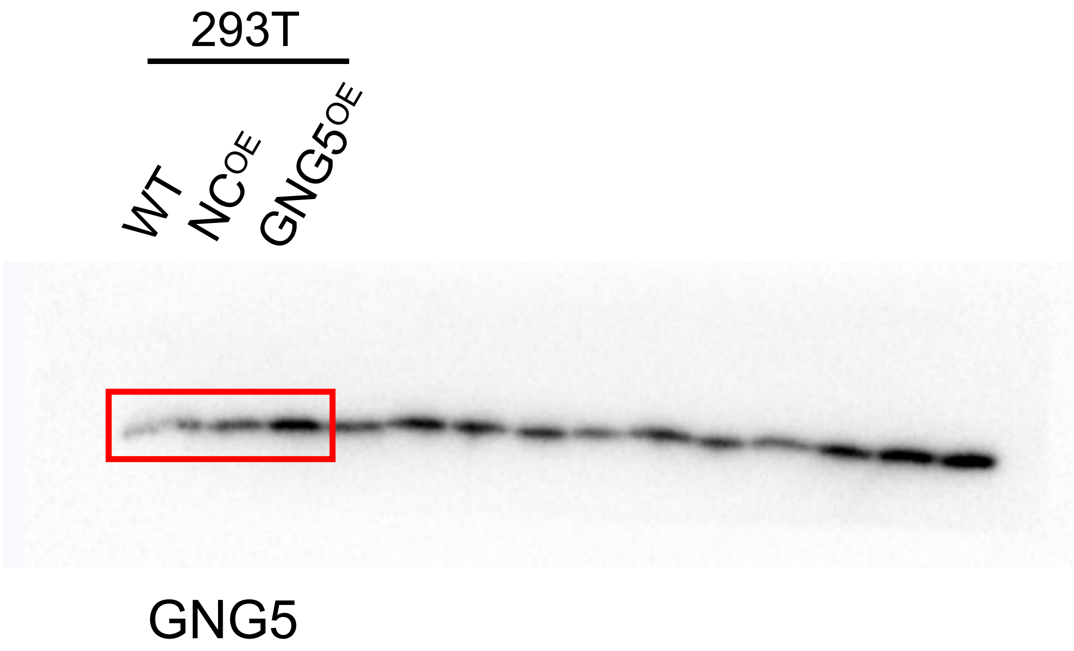

Figure 2K

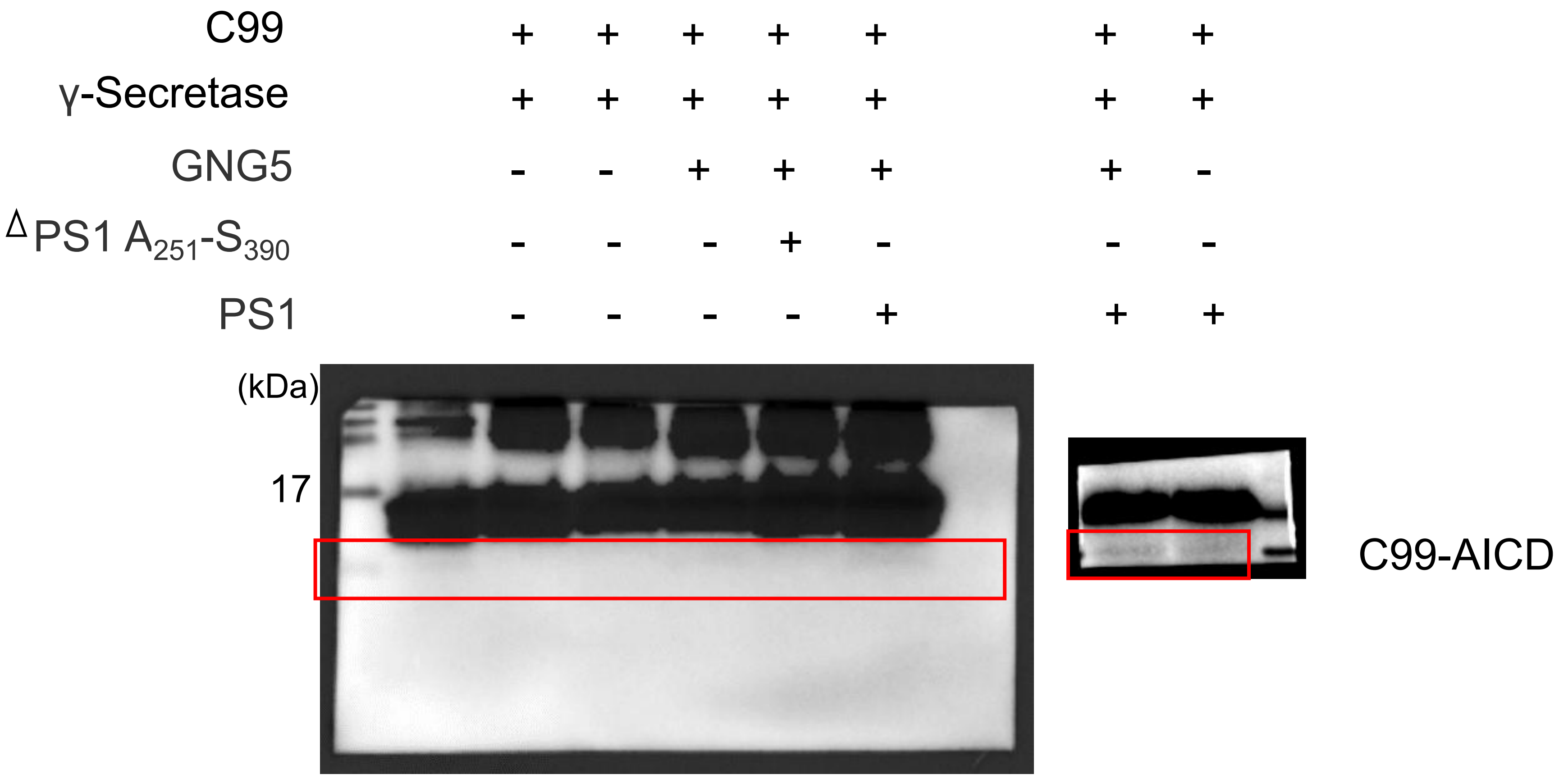

Figure 3B

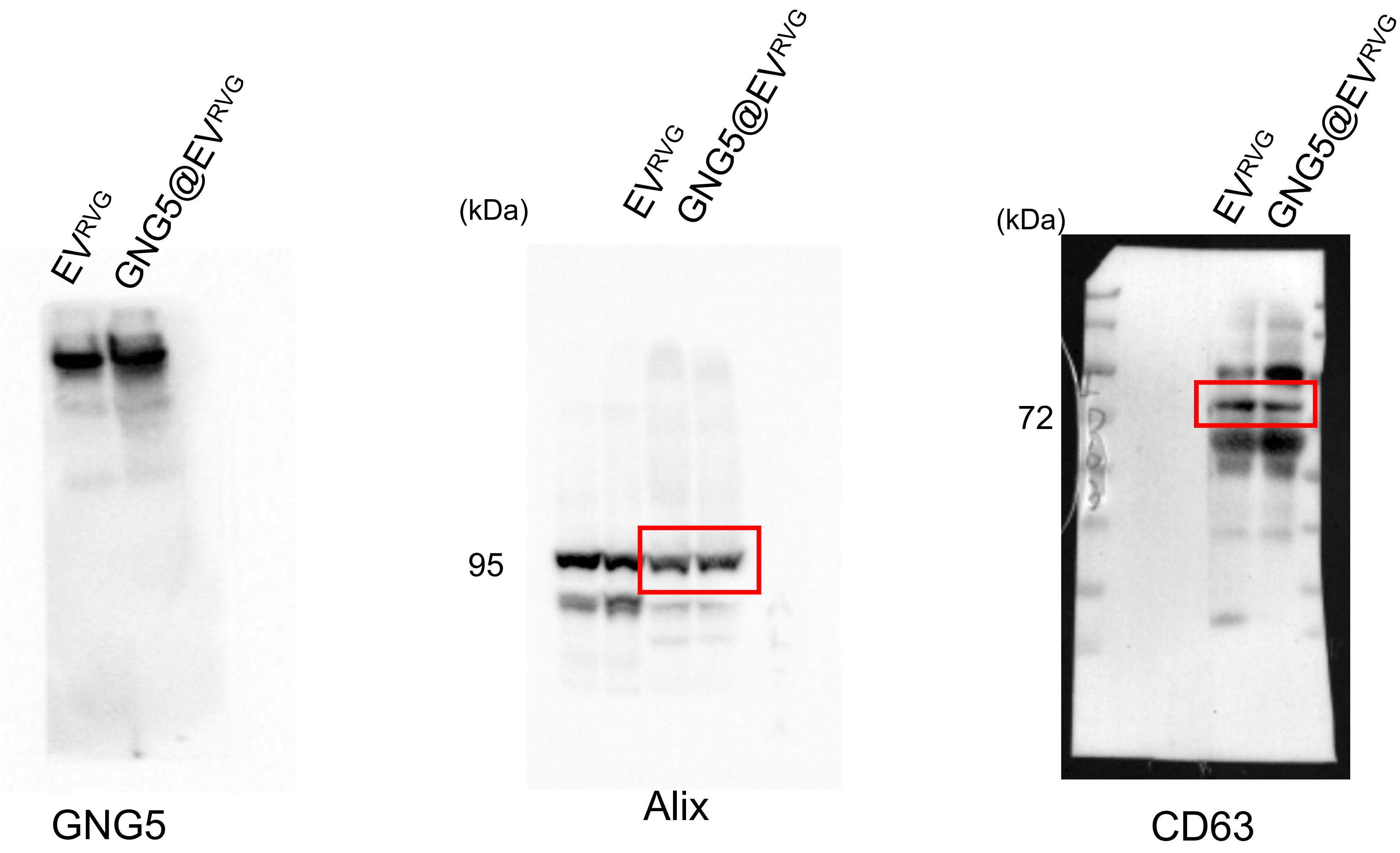

Figure 3B

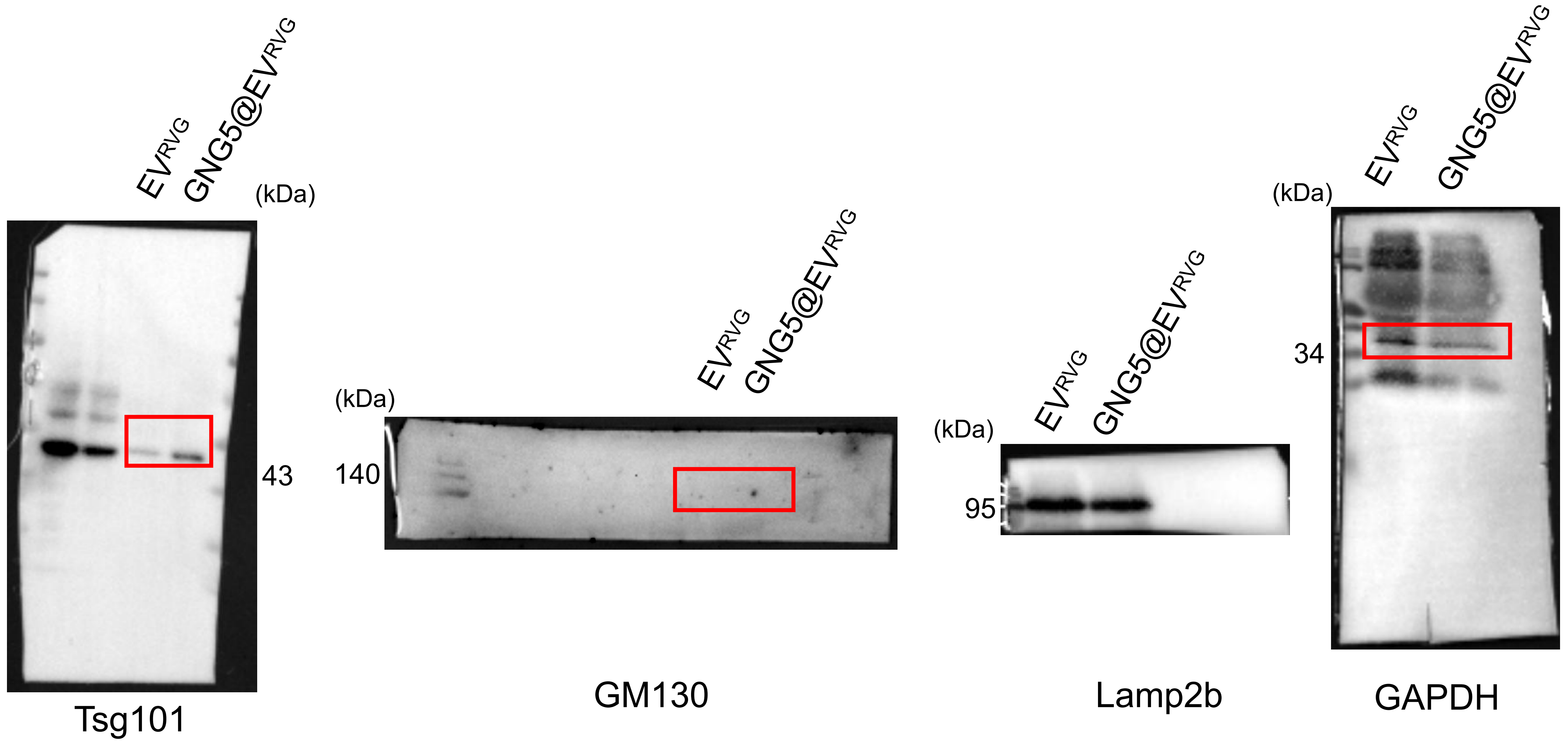

Figure 5B

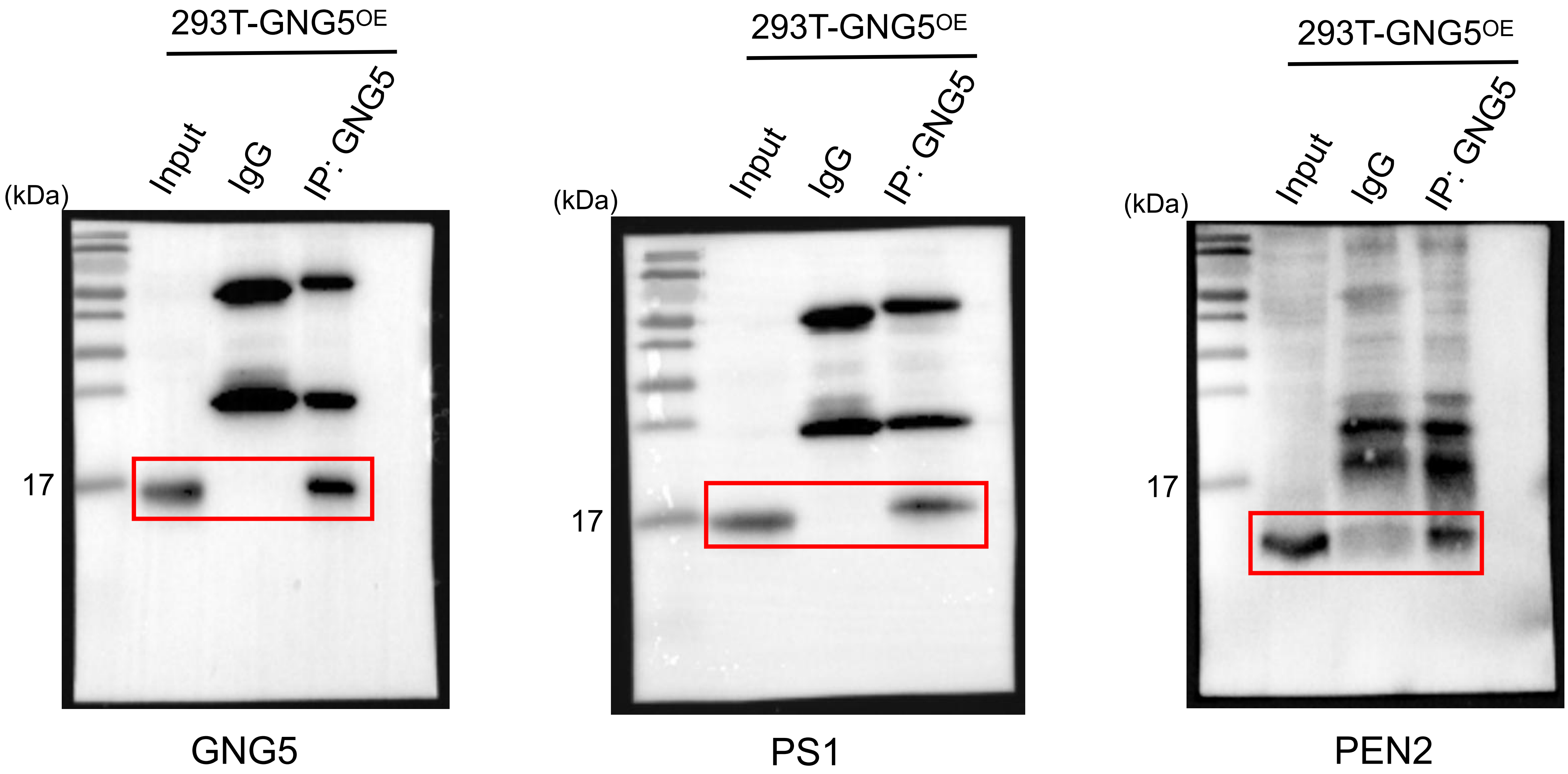

293T-GNG5^{OE}
Input
IgG
IP: GNG5
(kDa)
17
GNG5
293T-GNG5^{OE}
Input
IgG
IP: GNG5
(kDa)
17
PS1
293T-GNG5^{OE}
Input
IgG
IP: GNG5
(kDa)
17
PEN2

Figure 5B

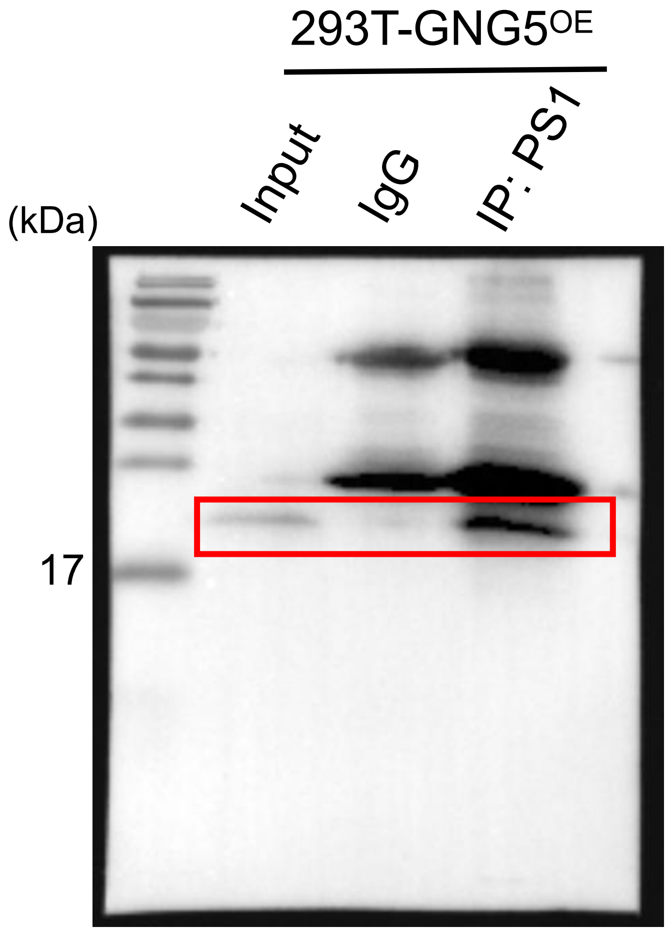

PS1

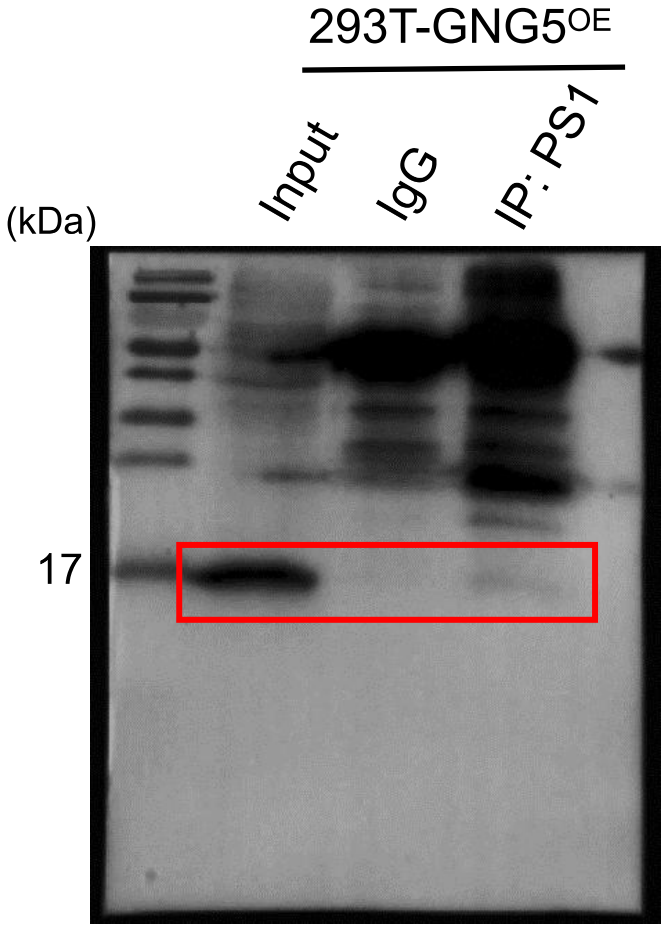

GNG5

Figure 6B

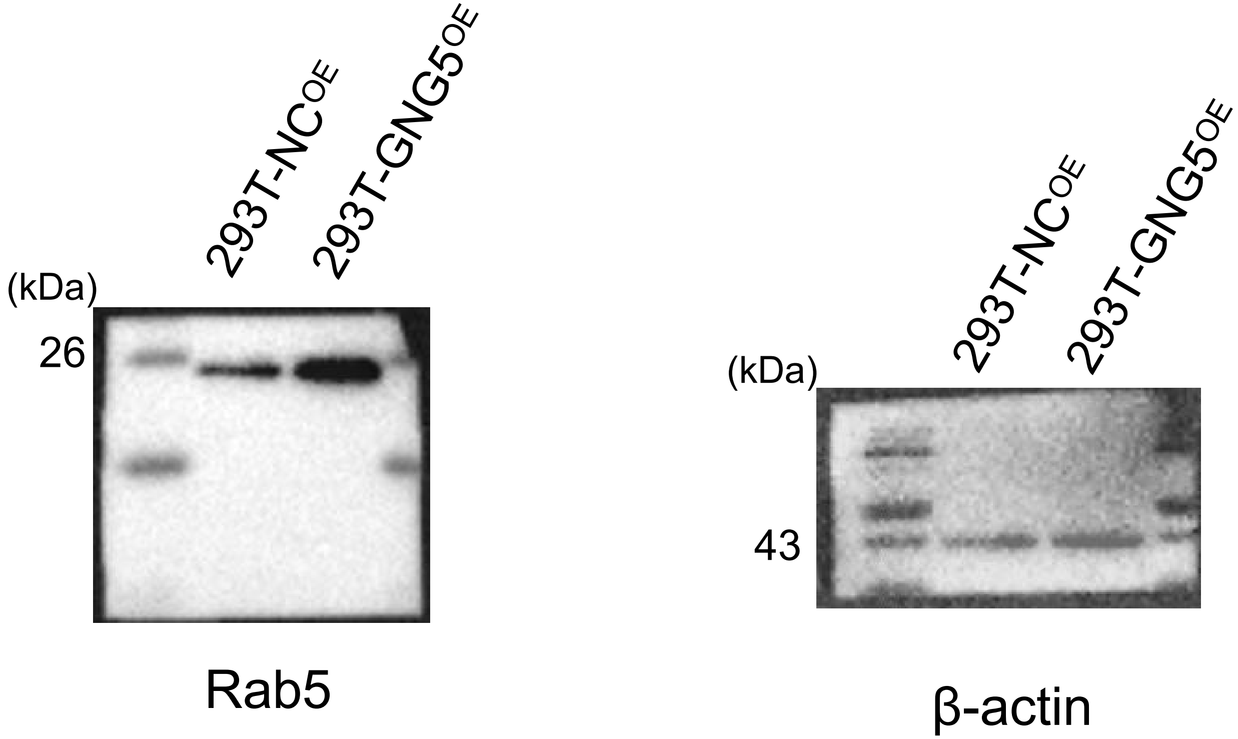

293T-NC^OE
293T-GNG5^OE
(kDa)
26
Rab5
293T-NC^OE
293T-GNG5^OE
(kDa)
43
β-actin

Figure 6D

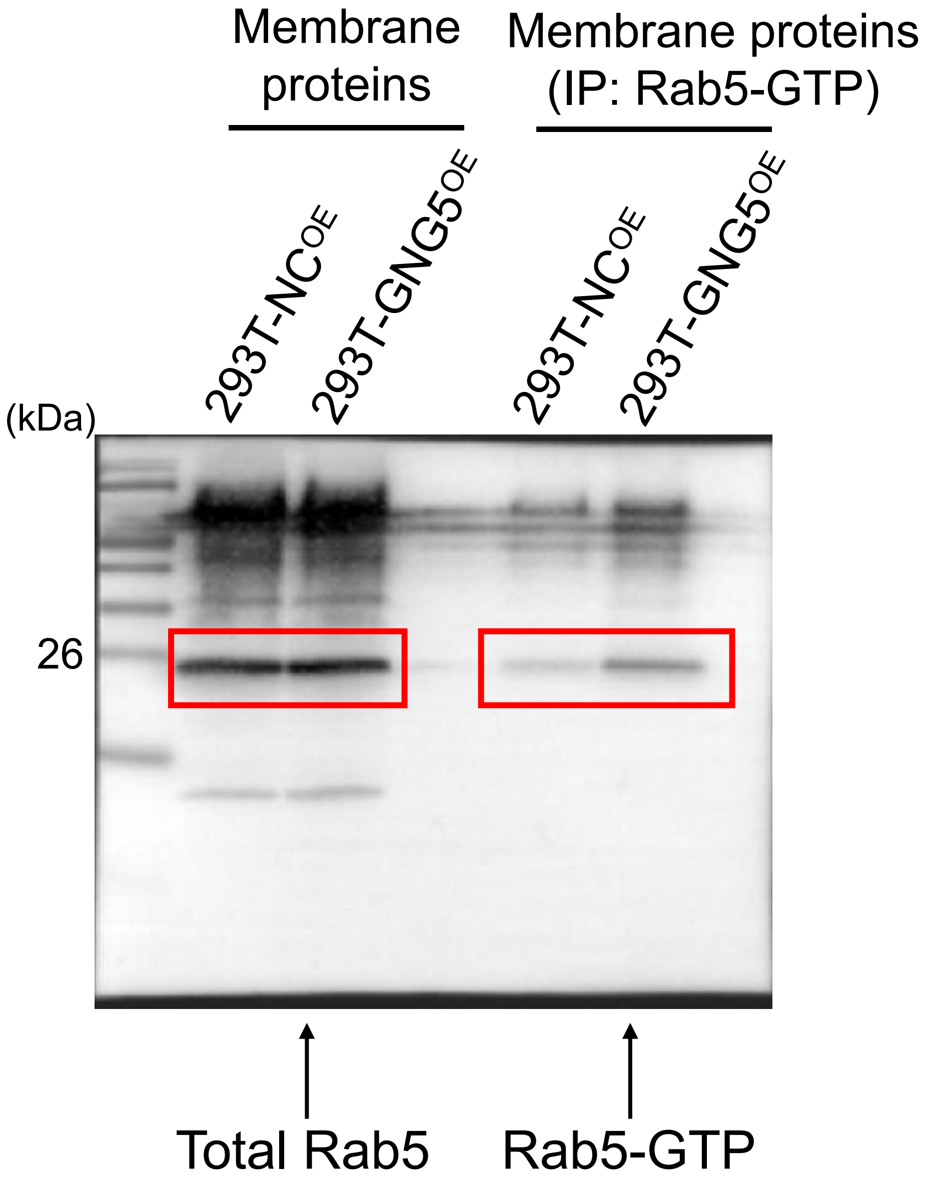
Membrane proteins
Membrane proteins (IP: Rab5-GTP)
293T-NCOE
293T-GNG5OE
293T-NCOE
293T-GNG5OE
(kDa)
26
Total Rab5
Rab5-GTP

Figure 6F

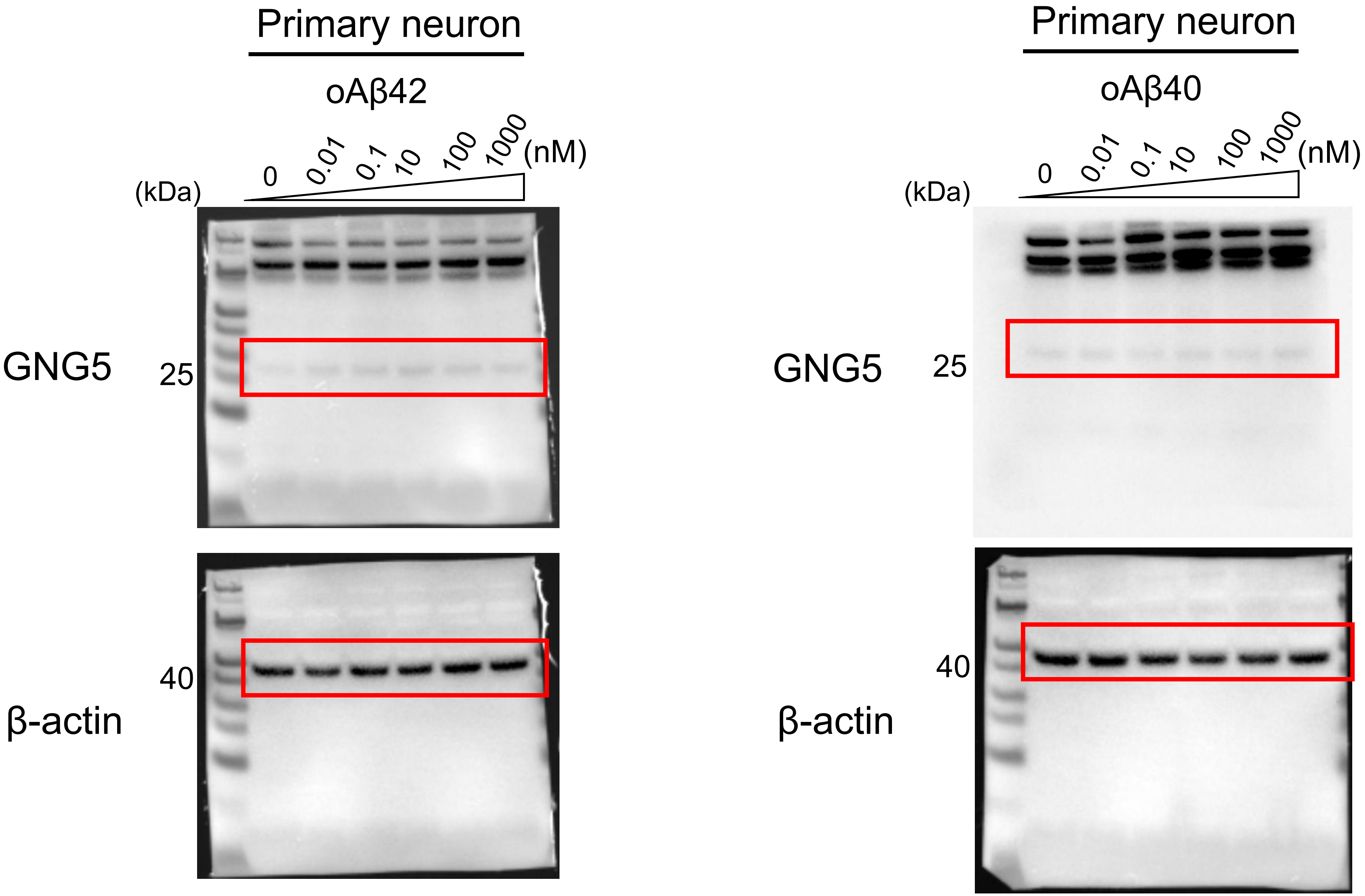
Primary neuron
oAβ42
0
0.01
0.1
10
100
1000
(nM)
(kDa)
GNG5
25
β-actin
40
Primary neuron
oAβ40
0
0.01
0.1
10
100
1000
(nM)
(kDa)
GNG5
25
β-actin
40

Figure 7A

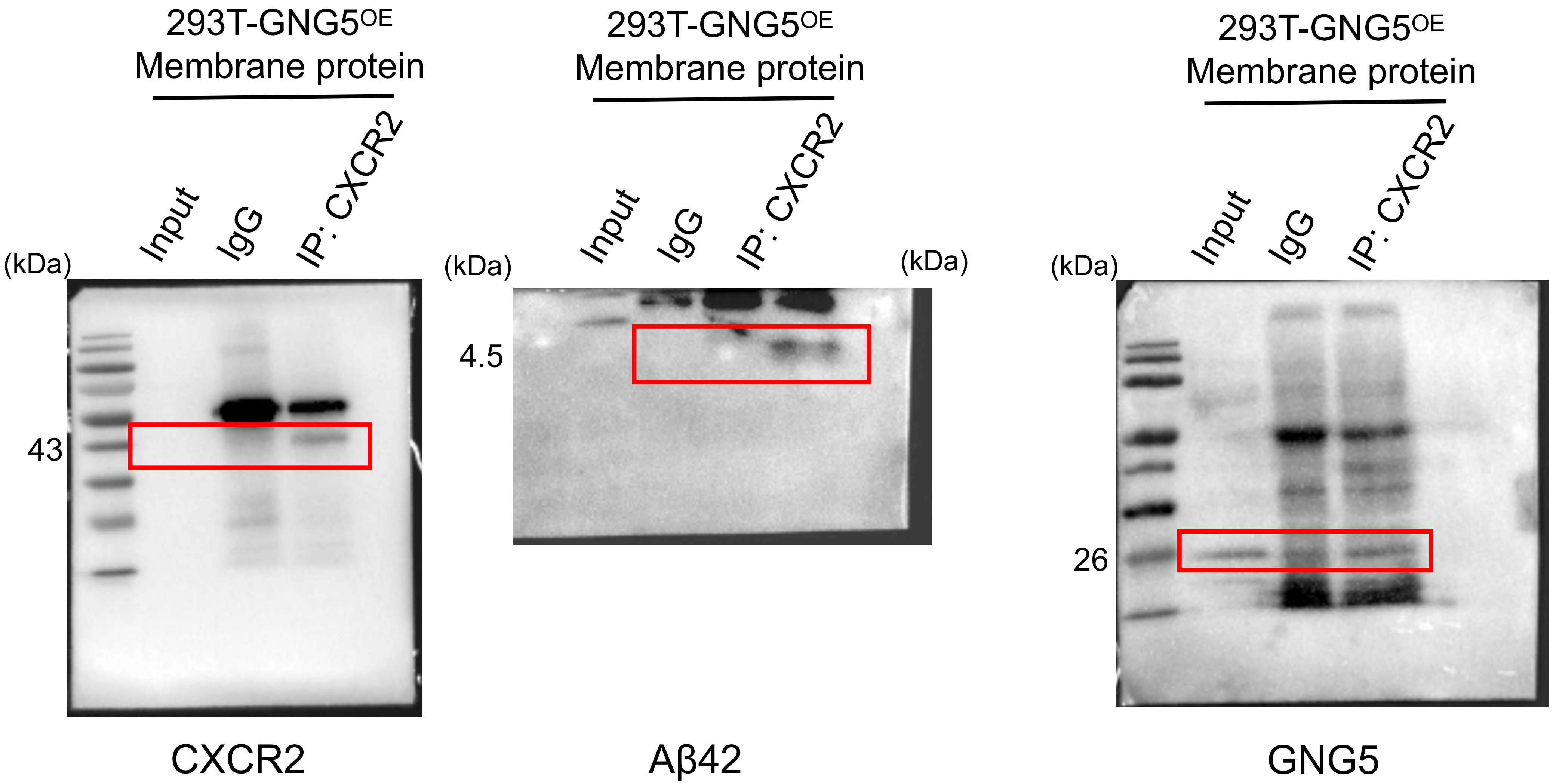
293T-GNG5OE
Membrane protein
Input
IgG
IP: CXCR2
(kDa)
43
CXCR2
293T-GNG5OE
Membrane protein
Input
IgG
IP: CXCR2
(kDa)
(kDa)
4.5
Aβ42
293T-GNG5OE
Membrane protein
Input
IgG
IP: CXCR2
(kDa)
26
GNG5

Figure 7C

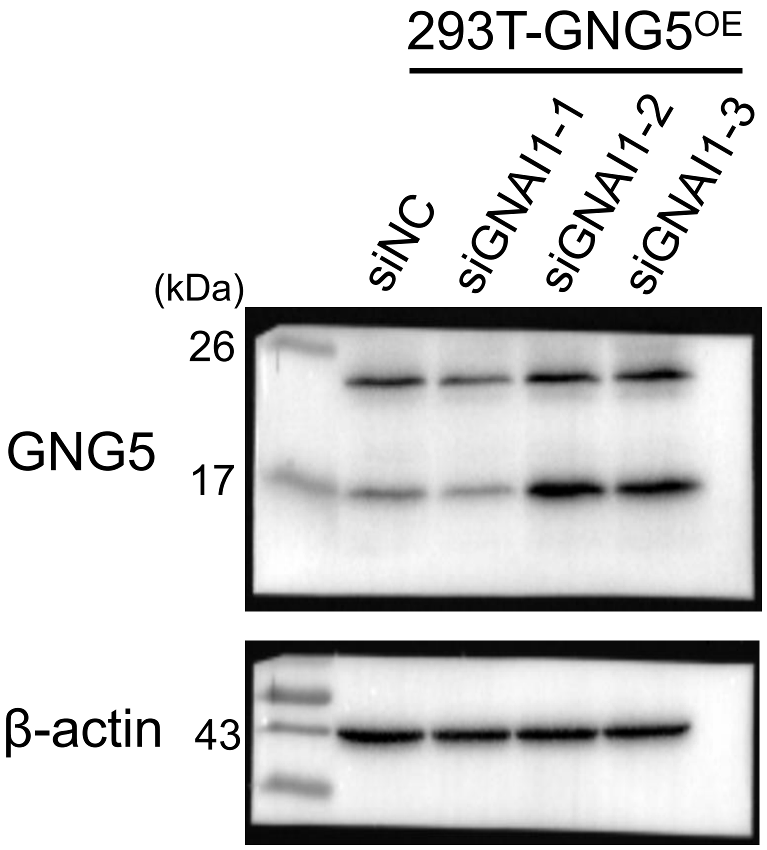
293T-GNG5OE
siNC
siGNAI1-1
siGNAI1-2
siGNAI1-3
(kDa)
26
GNG5
17
β-actin
43

Figure 7E

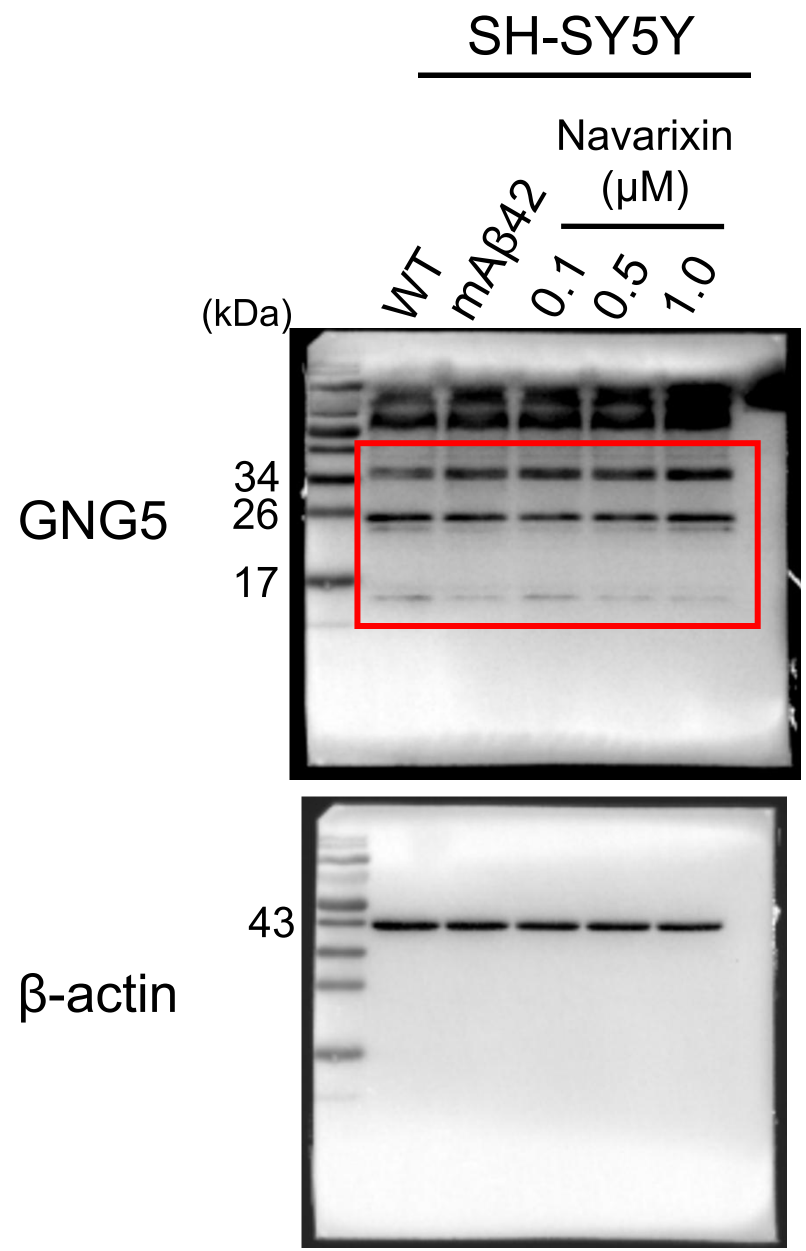
SH-SY5Y
Navarixin (μM)
WT
mAβ42
0.1
0.5
1.0
(kDa)
GNG5
34
26
17
β-actin
43

Figure 7H

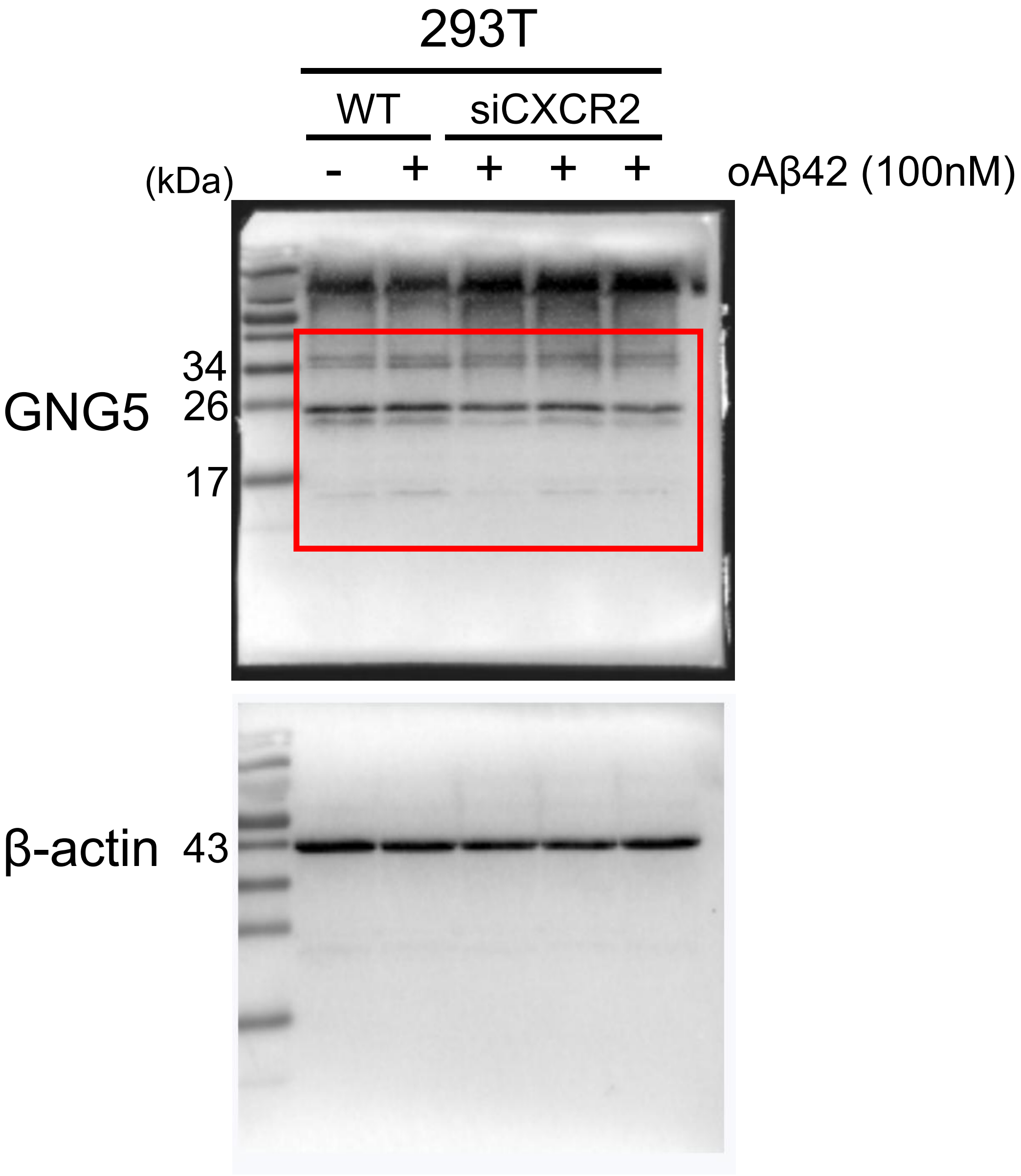

Figure 8C

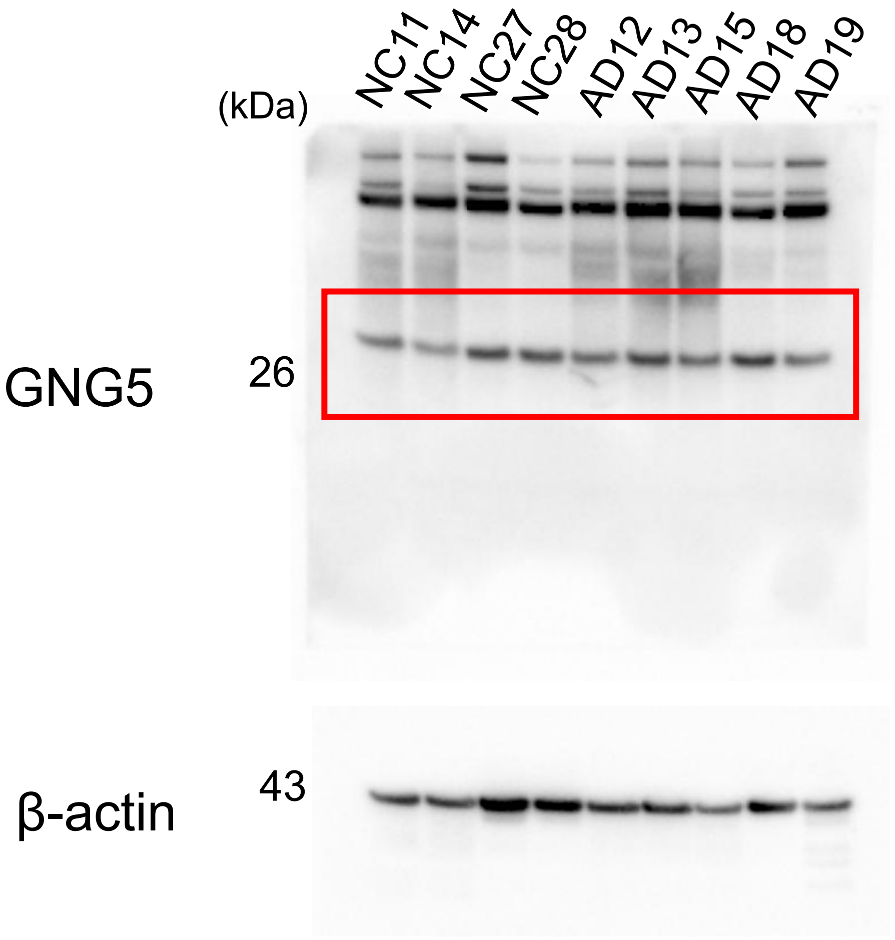

(kDa)
NC11
NC14
NC27
NC28
AD12
AD13
AD15
AD18
AD19
GNG5
26
β-actin
43

Figure 8F

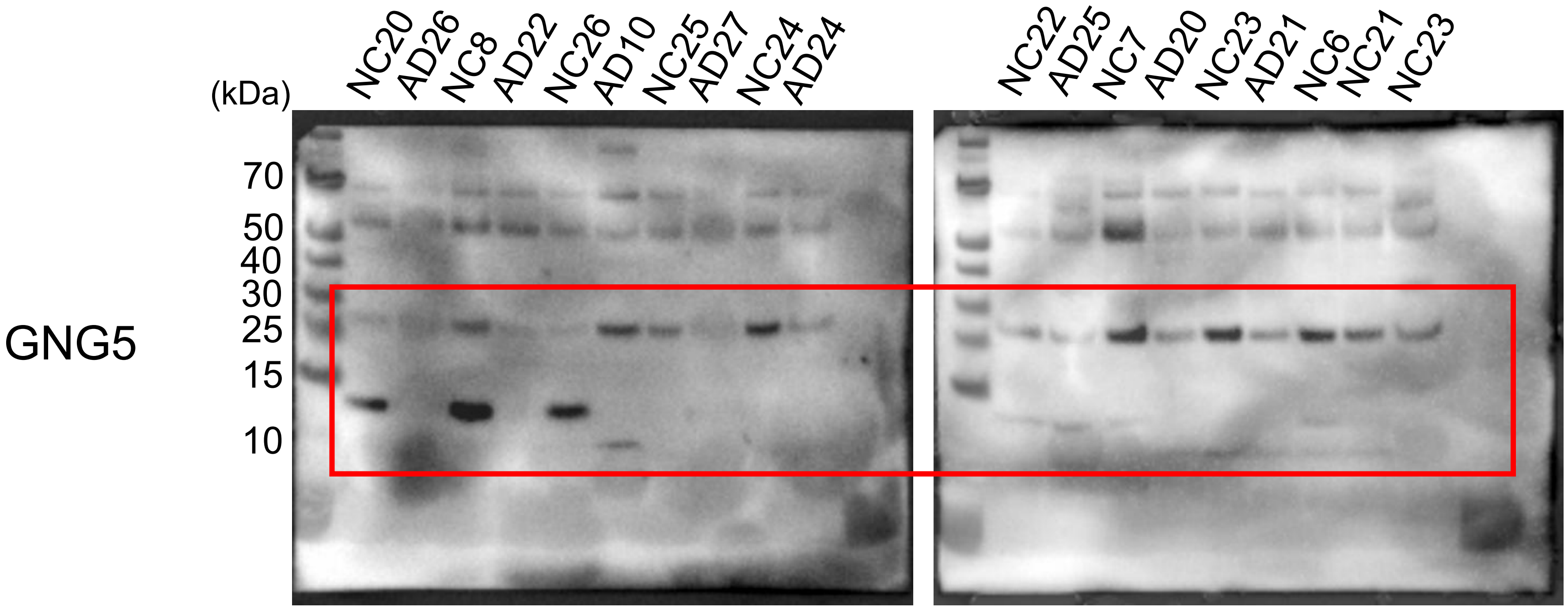

NC20
AD26
NC8
AD22
NC26
AD10
NC25
AD27
NC24
AD24
NC22
AD25
NC7
AD20
NC23
AD21
NC6
NC21
NC23
(kDa)
70
50
40
30
25
15
10
GNG5

Figure S3B

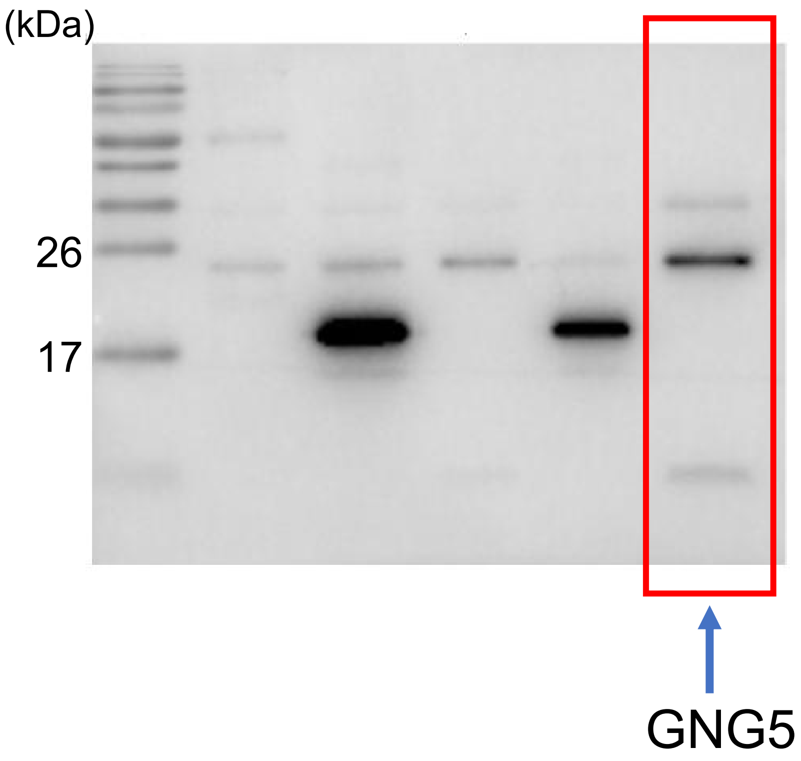

Figure S4G

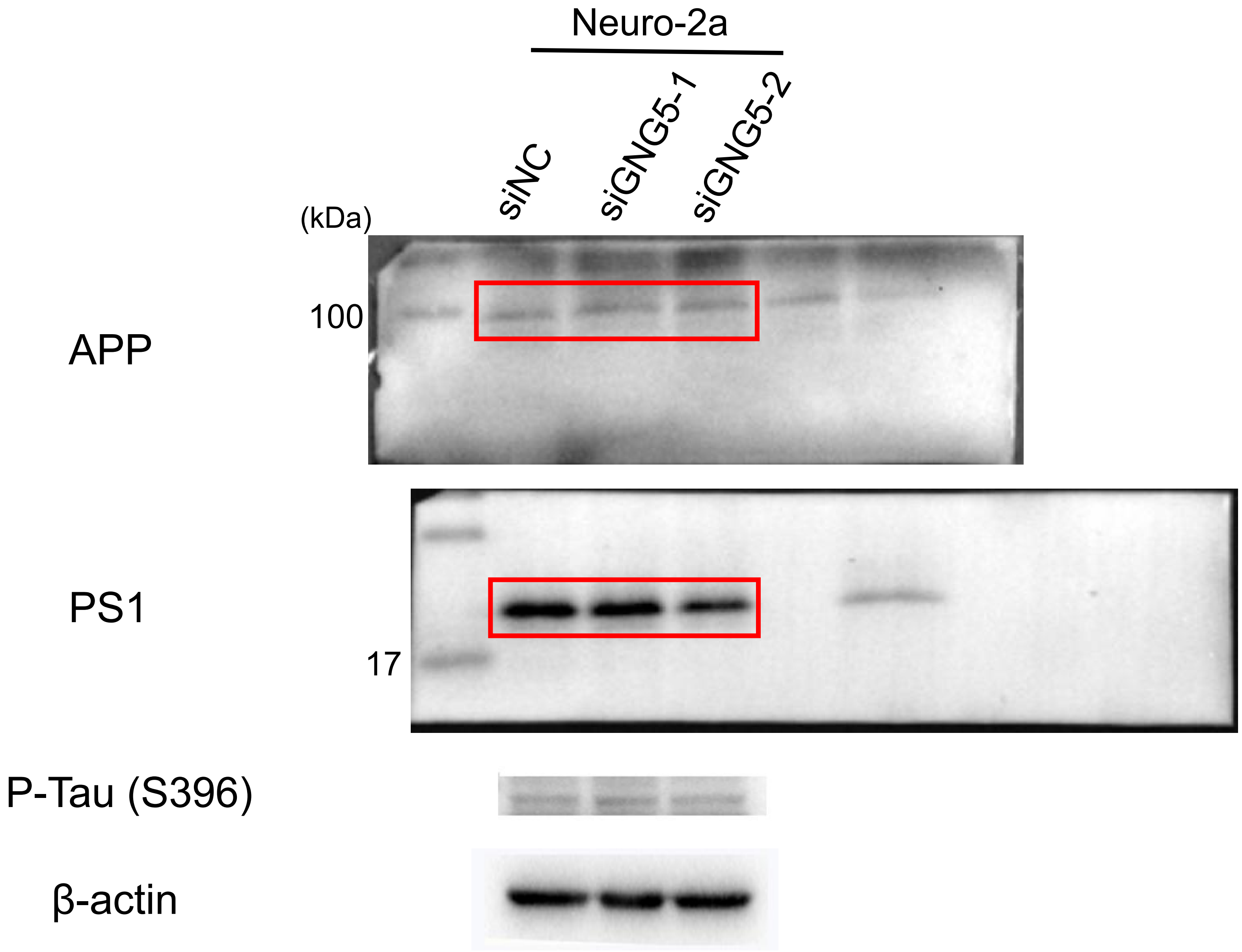

Neuro-2a
siNC
siGNG5-1
siGNG5-2
(kDa)
100
APP
PS1
17
P-Tau (S396)
β-actin

Figure S4I

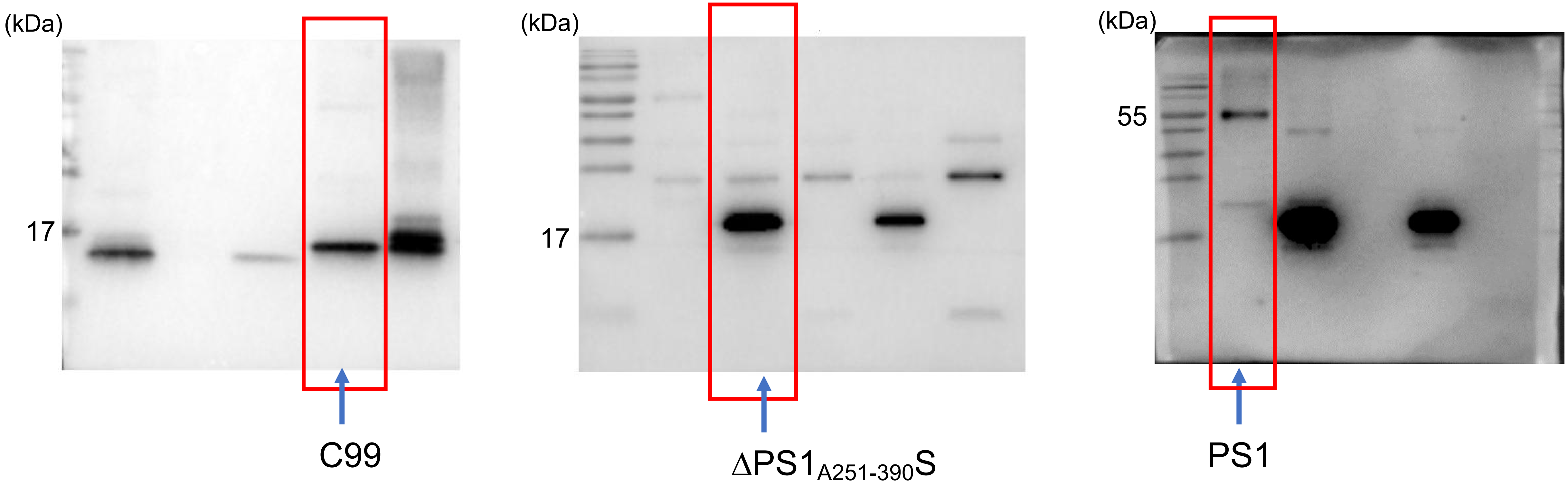

Figure S5A

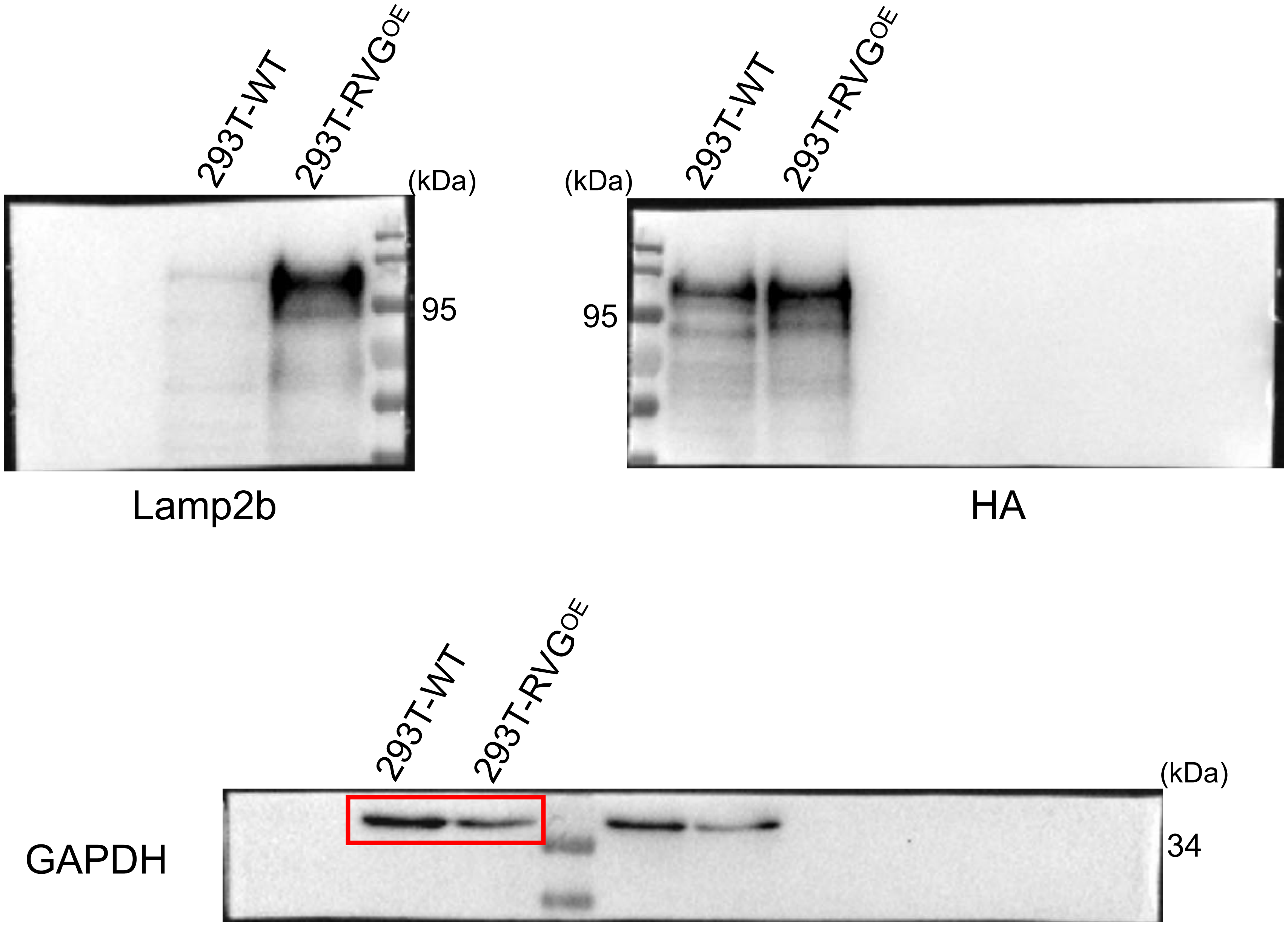
293T-WT
293T-RVGOE
(kDa)
95
Lamp2b
(kDa)
293T-WT
293T-RVGOE
95
HA
293T-WT
293T-RVGOE
(kDa)
GAPDH
34

Figure S5B

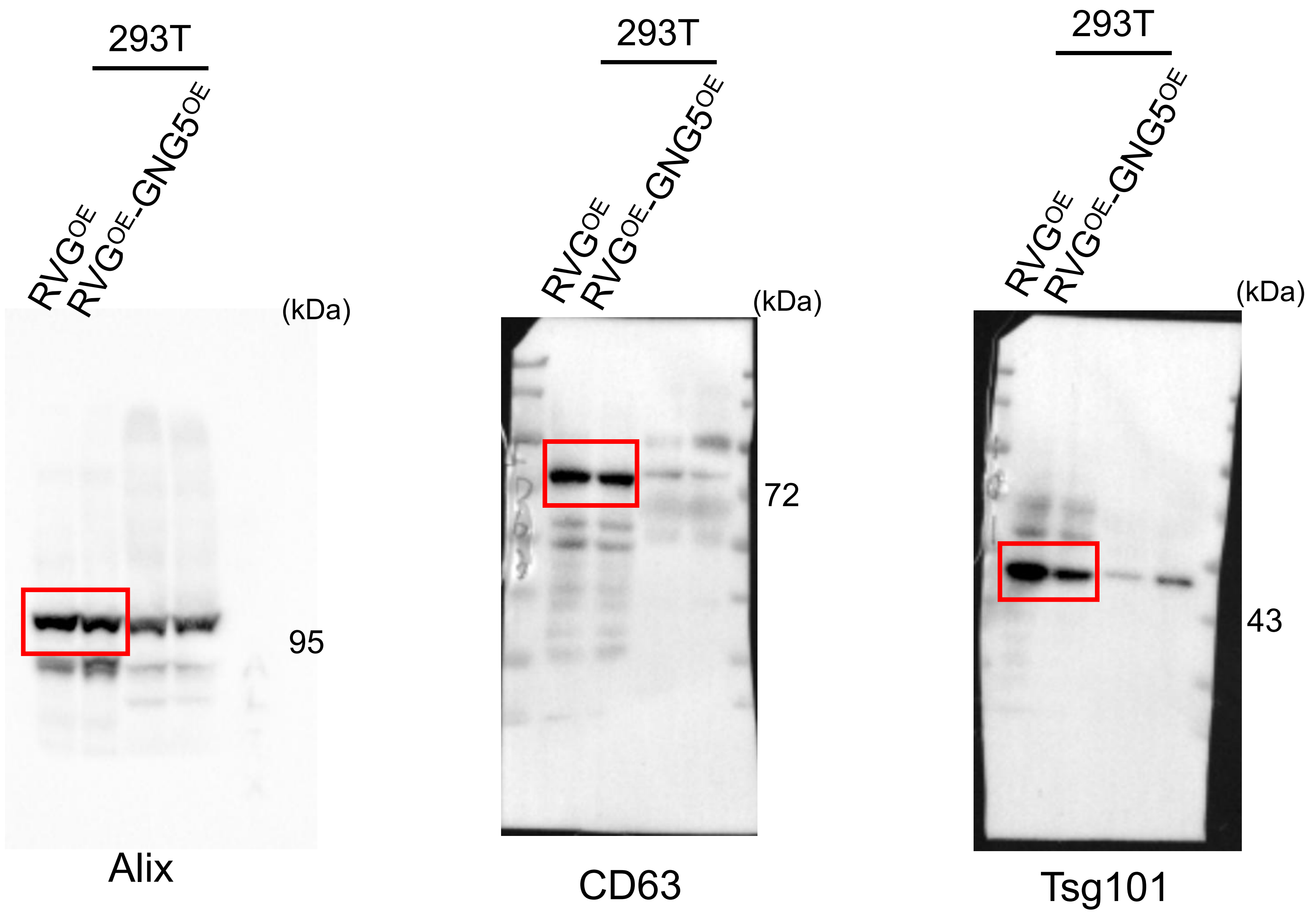

293T
RVGOE
RVGOE-GNG5OE
(kDa)
95
Alix
293T
RVGOE
RVGOE-GNG5OE
(kDa)
72
CD63
293T
RVGOE
RVGOE-GNG5OE
(kDa)
43
Tsg101

Figure S5B

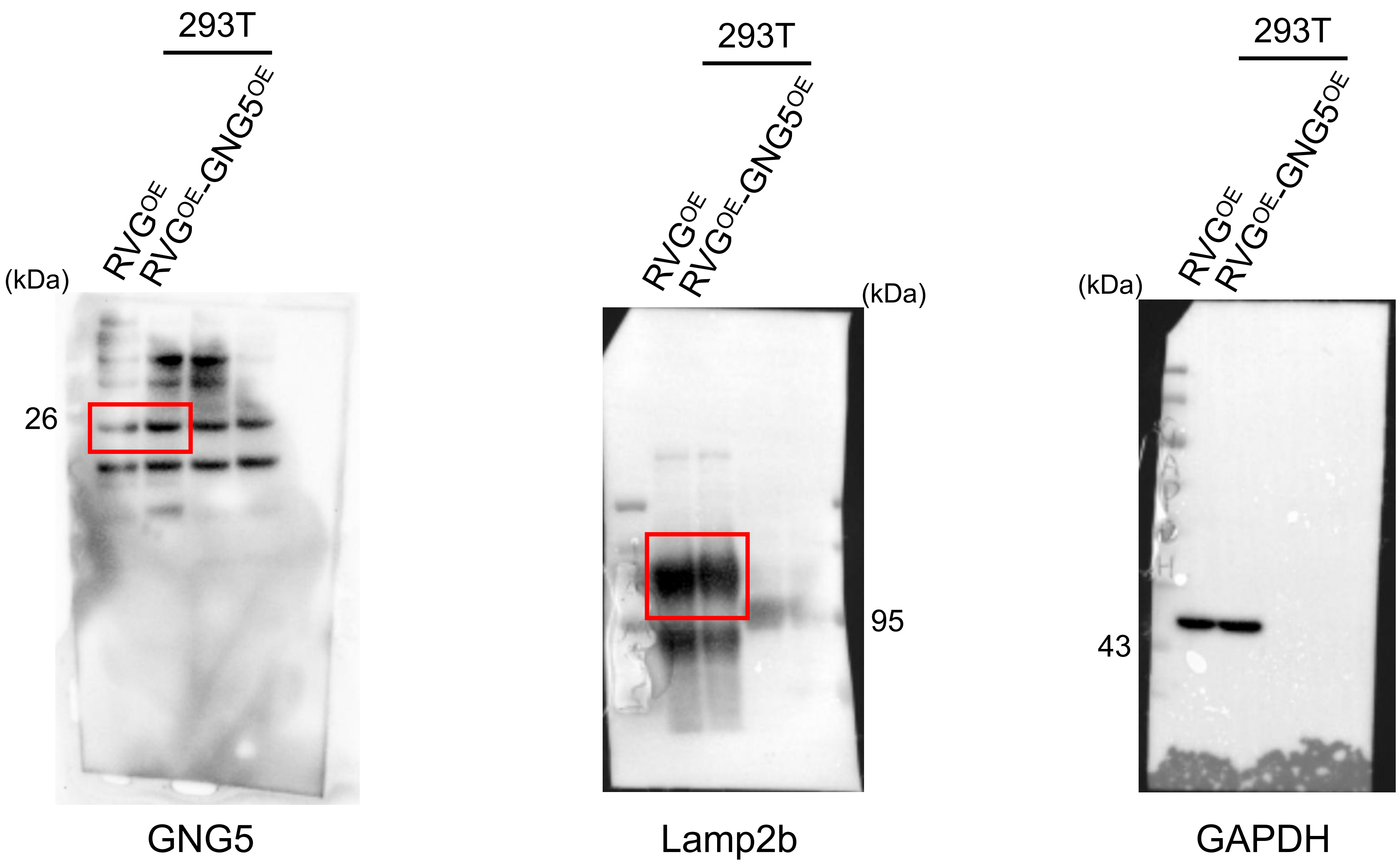
293T
RVGOE
RVGOE-GNG5OE
(kDa)
26
GNG5
293T
RVGOE
RVGOE-GNG5OE
(kDa)
95
Lamp2b
293T
RVGOE
RVGOE-GNG5OE
(kDa)
43
GAPDH

Figure S6B

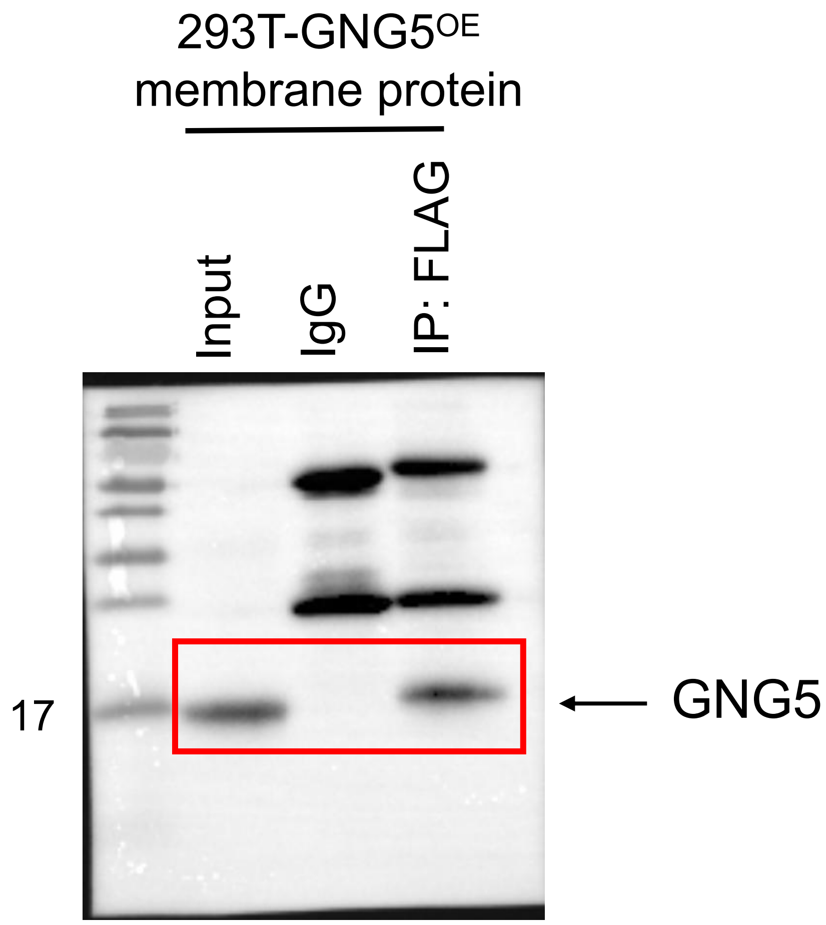

Figure S10G

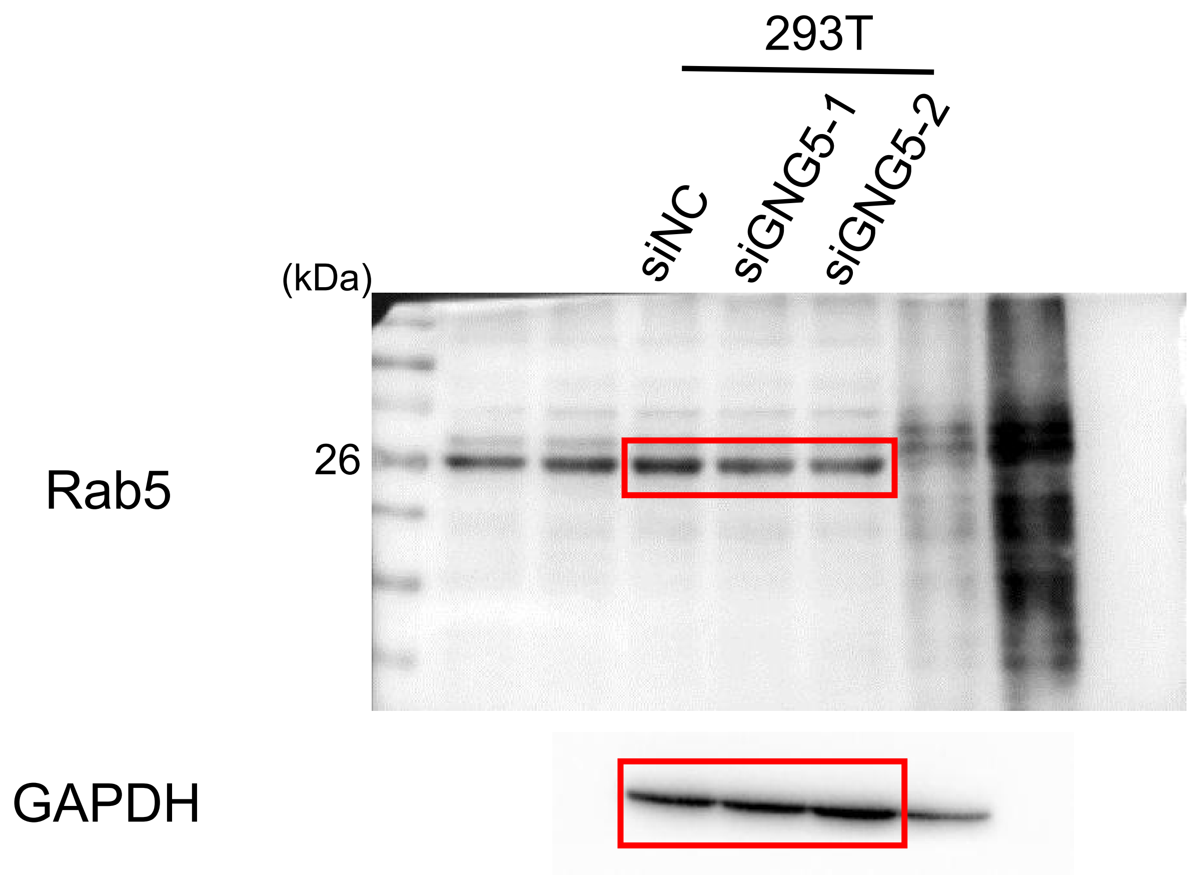

Figure S11C

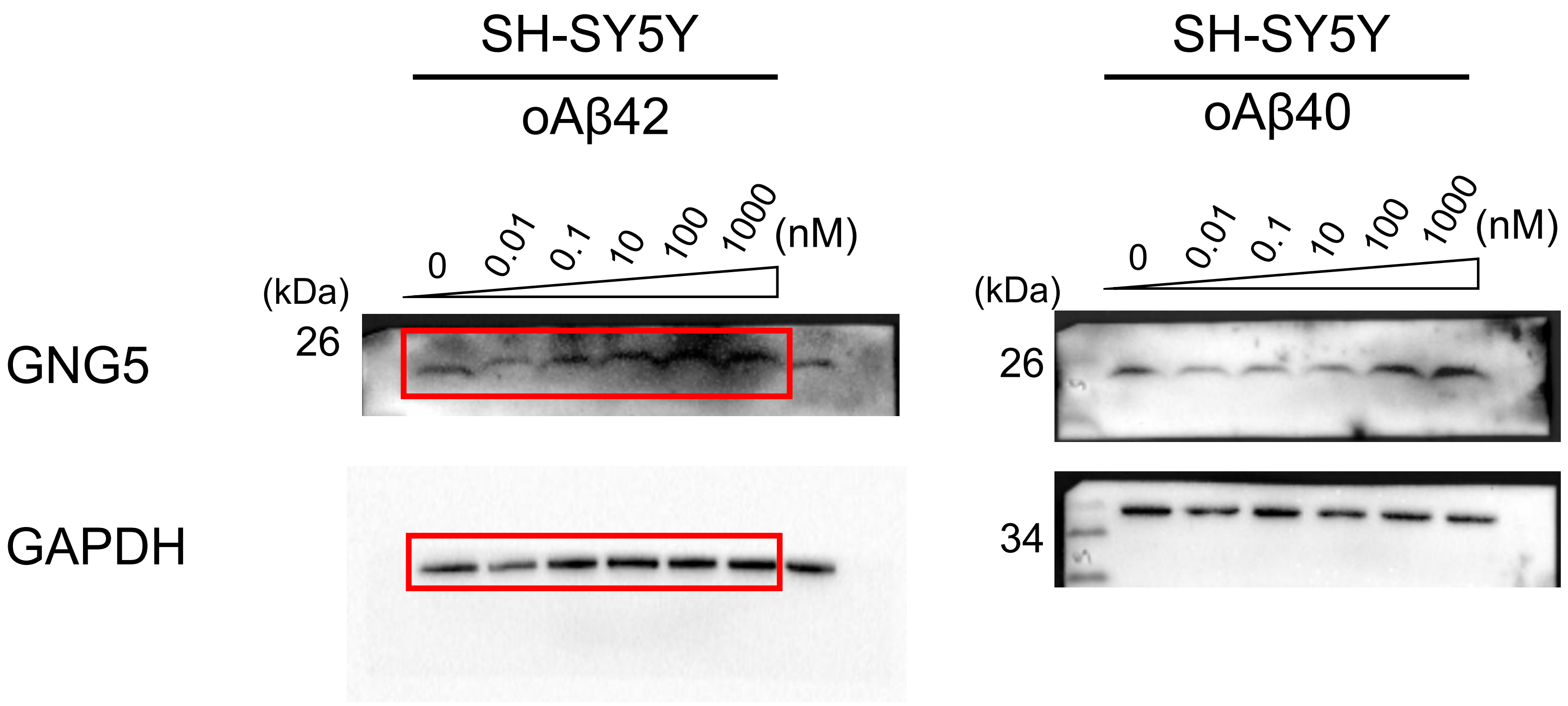
SH-SY5Y
oAβ42
0
0.01
0.1
10
100
1000
(nM)
(kDa)
26
GNG5
GAPDH
SH-SY5Y
oAβ40
0
0.01
0.1
10
100
1000
(nM)
(kDa)
26
34

Figure S11D

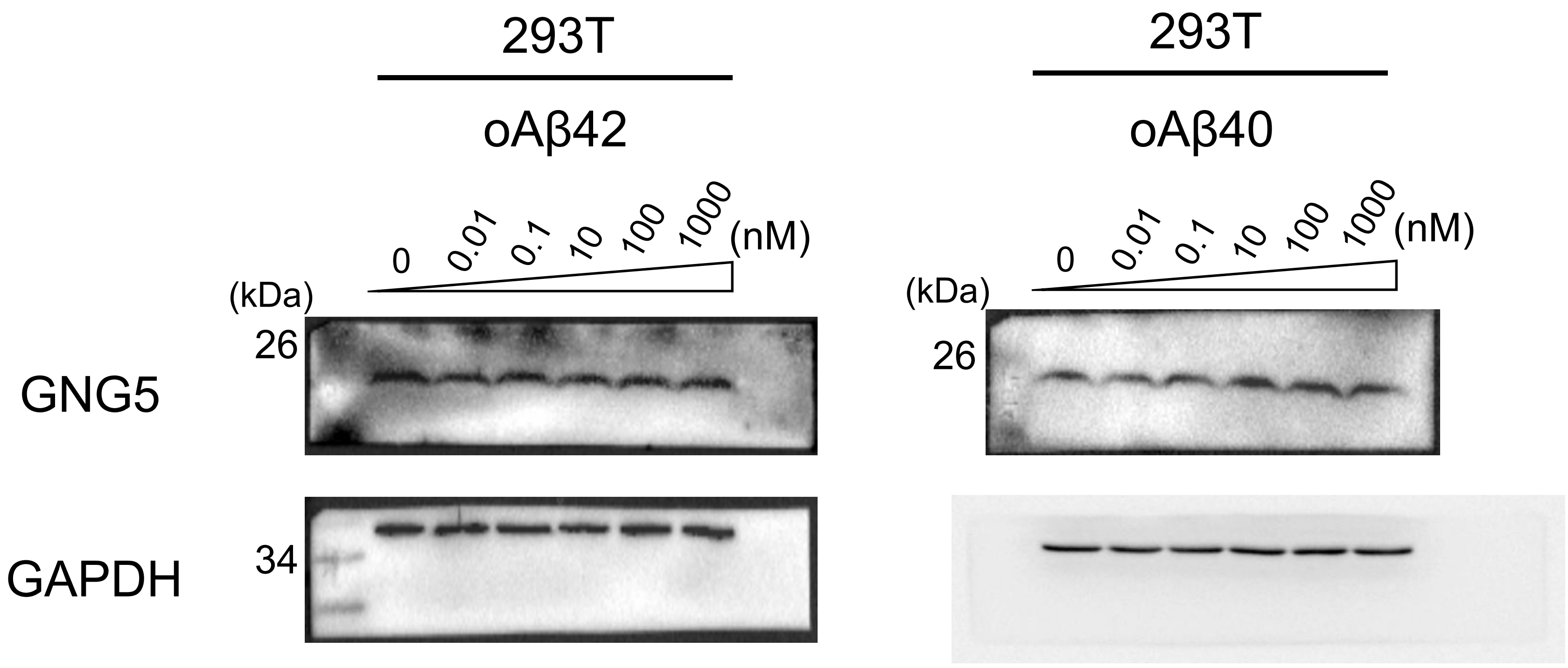
293T
oAβ42
0
0.01
0.1
10
100
1000
(nM)
(kDa)
26
GNG5
34
GAPDH
293T
oAβ40
0
0.01
0.1
10
100
1000
(nM)
(kDa)
26

Figure S11F

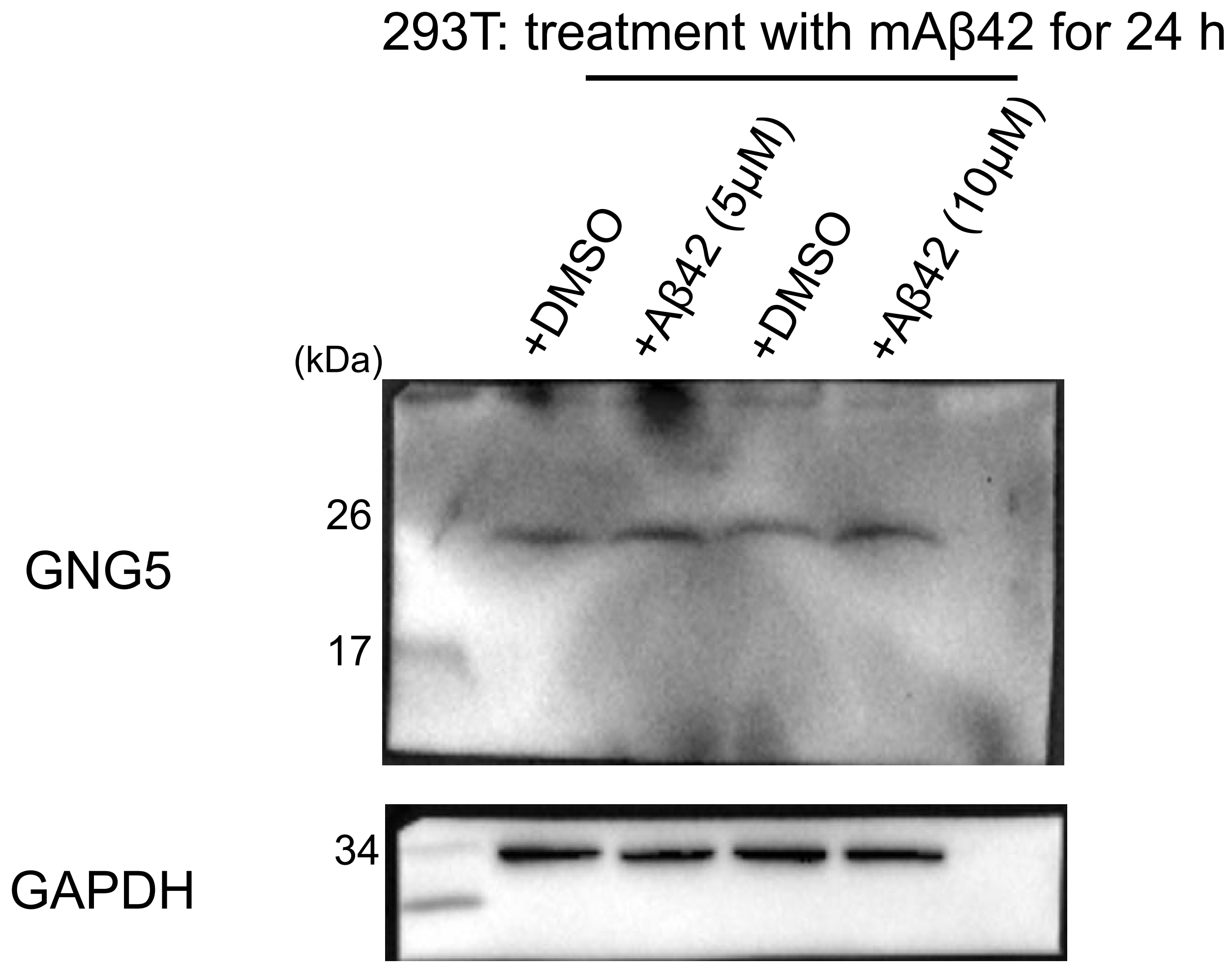
293T: treatment with mAβ42 for 24 h
+DMSO
+Aβ42 (5μM)
+DMSO
+Aβ42 (10μM)
(kDa)
26
17
GNG5
34
GAPDH

Figure S15D

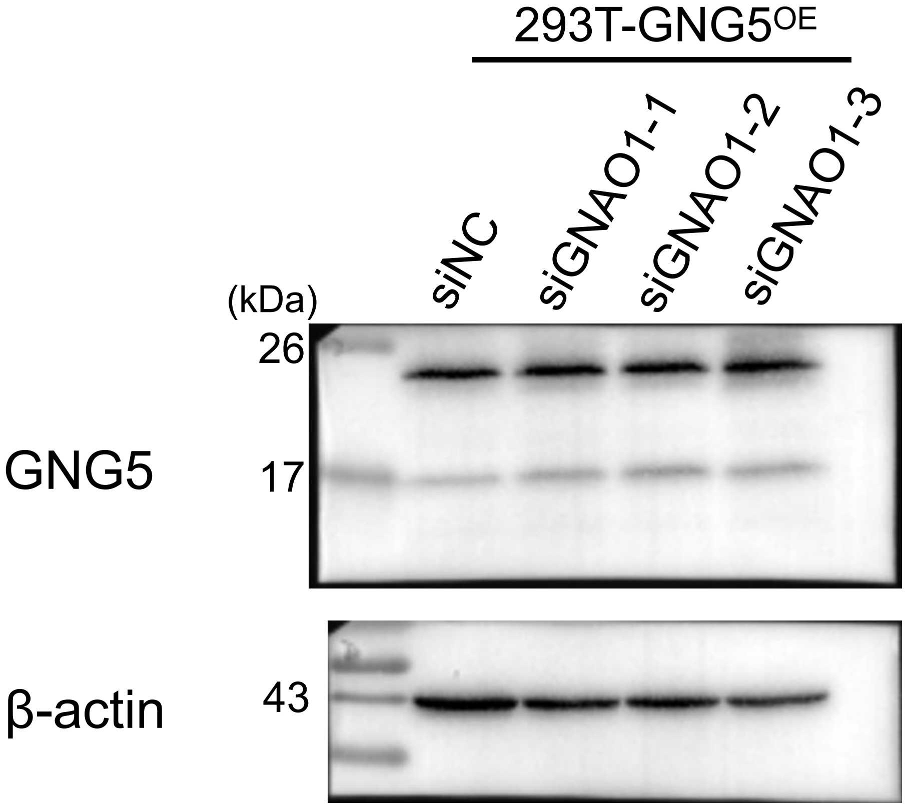

293T-GNG5OE
siNC
siGNAO1-1
siGNAO1-2
siGNAO1-3
(kDa)
26
GNG5
17
β-actin
43

Figure S15J

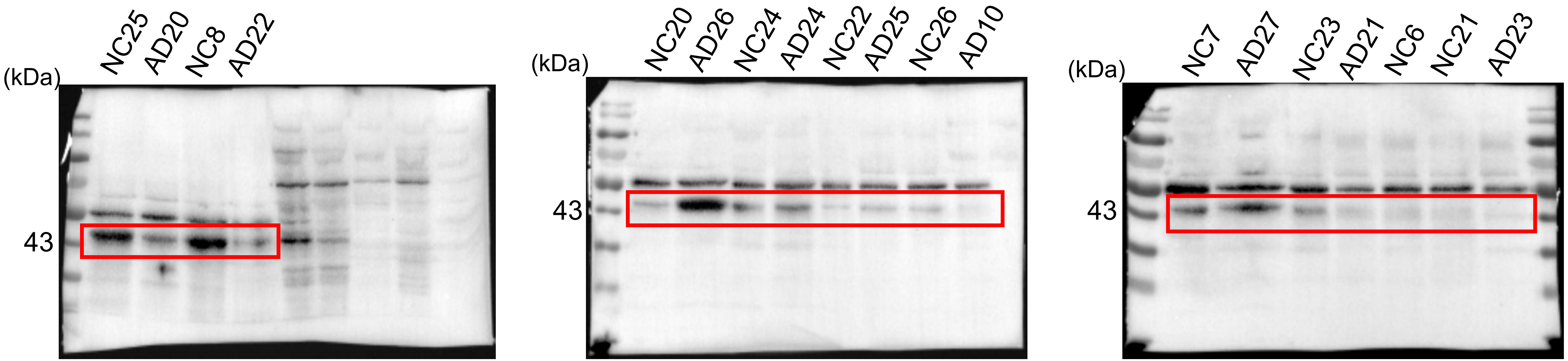

(kDa)
NC25
AD20
NC8
AD22
43
(kDa)
NC20
AD26
NC24
AD24
NC22
AD25
NC26
AD10
43
(kDa)
NC7
AD27
NC23
AD21
NC6
NC21
AD23
43

Figure S16E

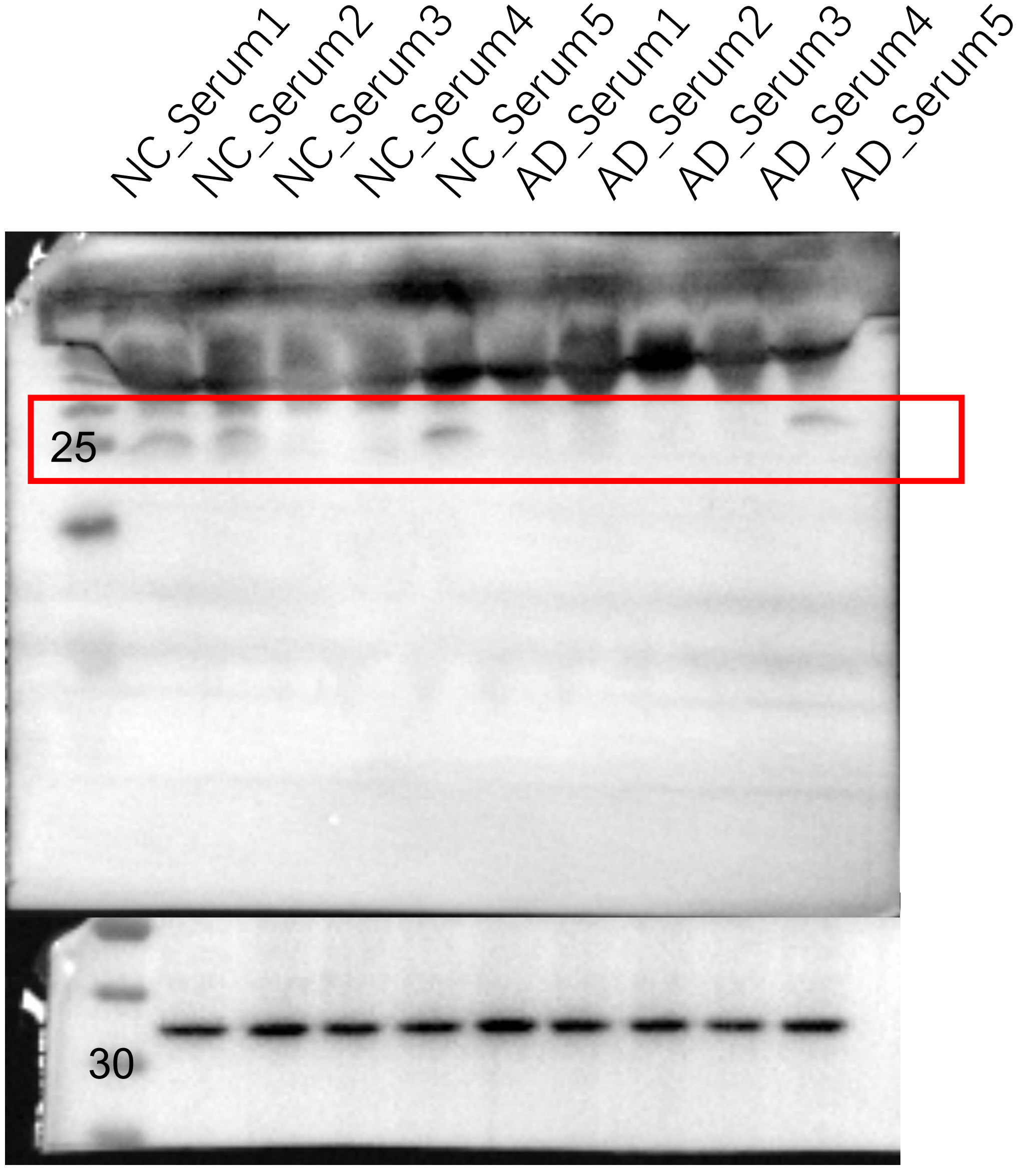

NC_Serum1
NC_Serum2
NC_Serum3
NC_Serum4
NC_Serum5
AD_Serum1
AD_Serum2
AD_Serum3
AD_Serum4
AD_Serum5
25
30
